# Photoredox Unmasking of Aromatic C–H Bonds in Living Environments Enabled by Thianthrenium Salts

**DOI:** 10.1021/jacs.6c00530

**Published:** 2026-02-07

**Authors:** Mauro Mato, Adrián Rivas-Saborido, Alba Casas-Pais, María Tomás-Gamasa, José L. Mascareñas

**Affiliations:** Centro Singular de Investigación en Química Biolóxica e Materiais Moleculares (CiQUS) and Departamento de Química Orgánica, 16780 Universidade de Santiago de Compostela, Santiago de Compostela 15705, Spain

## Abstract

Prodrug strategies traditionally rely on masking polar functional groups of bioactive molecules with protecting units that can be removed by specific stimuli in biological settings. Here, we introduce an alternative uncaging approach that bypasses the need for heteroatom handles, based on reversible masking of aromatic C–H bonds with thianthrenium groups. Unmasking is triggered by low-energy photoredox activation, which generates aryl radicals that are rapidly reduced by endogenous bioreductants to restore the native C–H bond. Beyond establishing the feasibility of photoredox radical chemistry in living cells, we demonstrate a proof-of-concept application of this strategy for the modulation of activity of antifungal agents.

## Graphical abstract

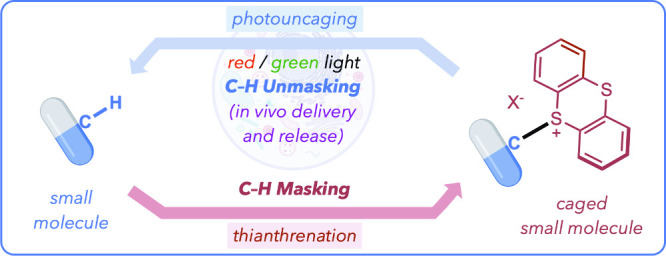

The prodrug concept, based on temporarily caging key functional groups in bioactive molecules, is central in chemical biology and medicine.^1^ The transient caging of pharmacophores not only enables external control of bioactivity, but also affects solubility, pharmacokinetics or transport.^2^ Many approaches exploit exogenous stimuli to release active *uncaged* molecules,^3^ being especially appealing those based on light-responsive systems, due to their ability to confer spatiotemporal control.^4^ While many of these methods rely on direct photochemical cleavage,^5^ *photocatalytic* approaches are gaining relevance, offering milder conditions and superior spatial resolution.^6^ The increasing impact of photocatalysis has facilitated diverse biological applications,^7^ including targeted biomolecule labeling,^8^ the direct induction of cancer-cell death (photodynamic therapy),^9^ or the intracellular synthesis of small molecules.^10^

Virtually all existing prodrug strategies, including photocatalytic uncaging, focus on protecting polar functional groups such as alcohols or amines in native drug structures (Figure 1A).^3h,7d,10b,11^ While powerful, this dependence on heteroatom handles limits the scope of molecules that can be derivatized, and the structural space of prodrugs that can be modified. We envisioned that this limitation could be addressed through alternative uncaging strategies based on reversible masking of specific C–H bonds (Figure 1B). However, identifying suitable groups that can be selectively installed at defined C–H positions and later removed under mild, biocompatible conditions represents a significant challenge.

**Figure 1:**
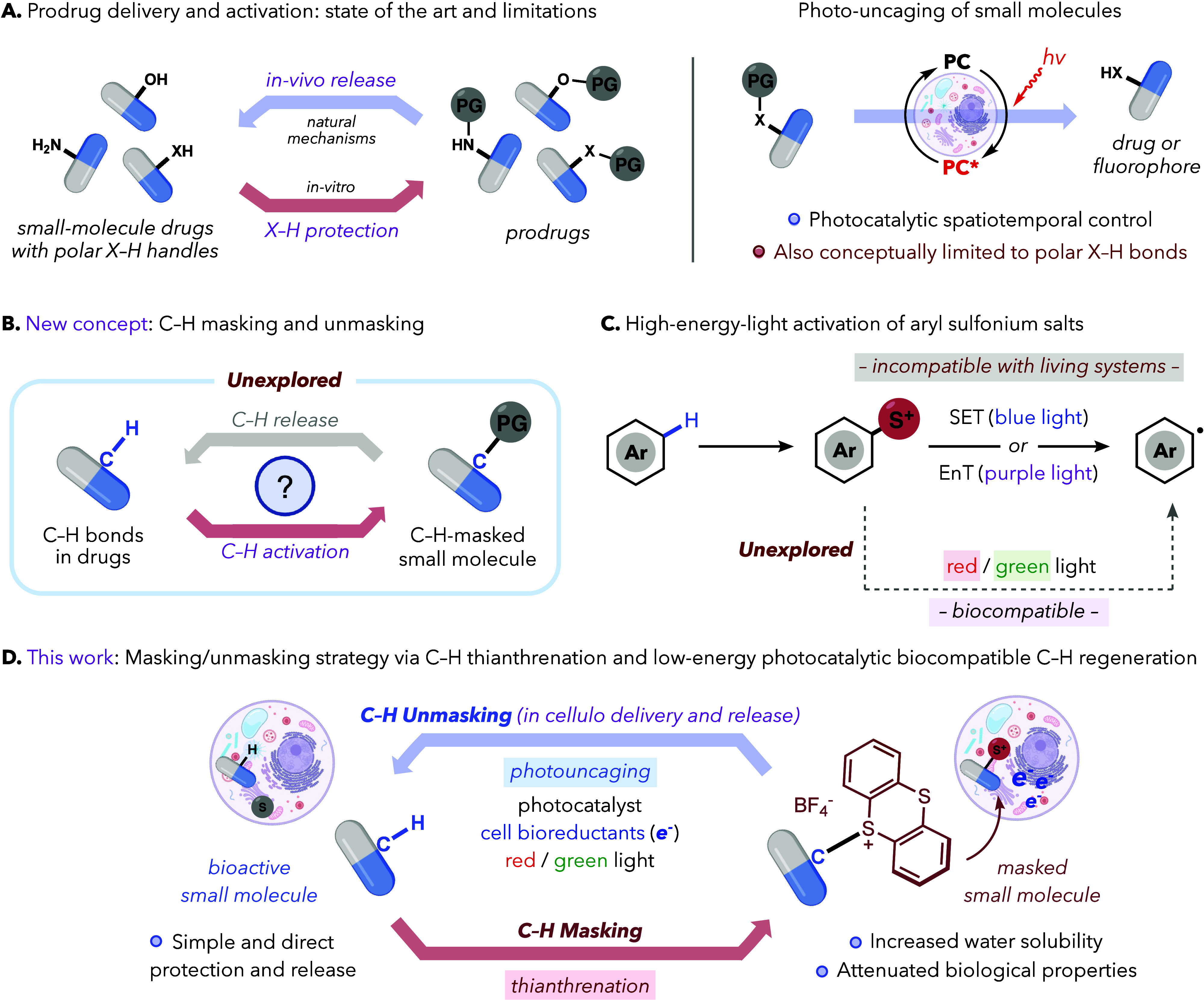
(**A**) Current prodrug strategies based on masking polar functional groups and light-triggered uncaging. (**B**) Our strategy: protection and release of C–H bonds. (**C**) Previous photoactivation of aryl-thianthrenium salts and limitations. (**D**) C–H masking/unmasking based on the photoredox activation of aryl-sulfonium prodrugs. PG = protecting group; PC = photocatalyst; S^+^ = thianthrenium; EnT = energy transfer.

Inspired by recent advances in aromatic C–H thianthrenation reactions in the context of organic synthesis,^12^ pioneered by the groups of Ritter,^13^ Alcarazo,^14^ and Procter,^15^ among others (Figure 1C),^16^ we hypothesized that anchoring this type of bulky, positively charged sulfonium group at selected aromatic positions in drugs could attenuate their biological activity while enhancing water solubility. More importantly, the resulting aryl-thianthrenium salts might be readily reverted to the parent aromatic precursors by photoredox catalysis under appropriate reducing conditions.^17^ Unfortunately, photocatalytic activation of aryl thianthrenium salts has so far been limited to organic solvents and high-energy light irradiation, precluding its use in biological contexts.^16,18^

Herein we show that these sulfonium salts can also be activated by low-energy visible light under aqueous, biorelevant conditions, by leveraging endogenous bioreductants, such as NADH, which enable reductive-quenching photoredox manifolds.^4,10b,19^ Importantly, the reactions, entailing aryl radicals, can even be performed inside living mammalian cells (Figure 1D). Moreover, we have explored the potential of this C–H masking/unmasking strategy, based on thianthrenation and photoredox uncaging, for the controlled activation of antifungal compounds.

The photoredox uncaging was first studied in the model sulfonium salt **2a**, accessible as a single regioisomer by C–H thianthrenation of pyriproxyfen (**1a**).^13,20^ Despite the relatively negative reduction potential of these substrates (*E*_*p/2*_ ≈ −1.0 V vs SCE),^21^ a range of green- and red-light-harvesting photocatalysts efficiently promoted the reaction under open-air conditions in a DMSO/water mixture (Figure 2A). Irradiation of **2a** with green (525 nm) or red (660 nm) LEDs in the presence of DIPEA and photocatalysts like Eosin Y (**3**), ZnTPP (**4**) or methylene blue (MB, **5**) led to the quantitative release of the parent pyriproxyfen and the thianthrene unit (TT) (Entries 1 and 5). Control experiments without photocatalyst, reductant or light showed only traces of product with the green-light-based system, and no reactivity with the red-light manifold (Entries 2 and 6, respectively). Kinetic evaluation indicated completion within 30–60 min for both systems (Entries 3 and 7). The uncaging also proceeded using BnAH (**6**) or, importantly, the endogenous reductant NADH, instead of DIPEA (Entries 4, 8 and 9). In terms of bioorthogonality, the reaction can be successfully performed in a mixture of DMSO and DMEM (Dulbecco’s Modified Eagle’s Medium), a cell-culture medium containing a variety of biomolecules and reductants (Entry 10). These results prompt well for the potential use of this photoredox chemistry in biorelevant contexts, and eventually inside live cells, which are the more demanding environments in terms of biomolecular complexity (see below).

**Figure 2:**
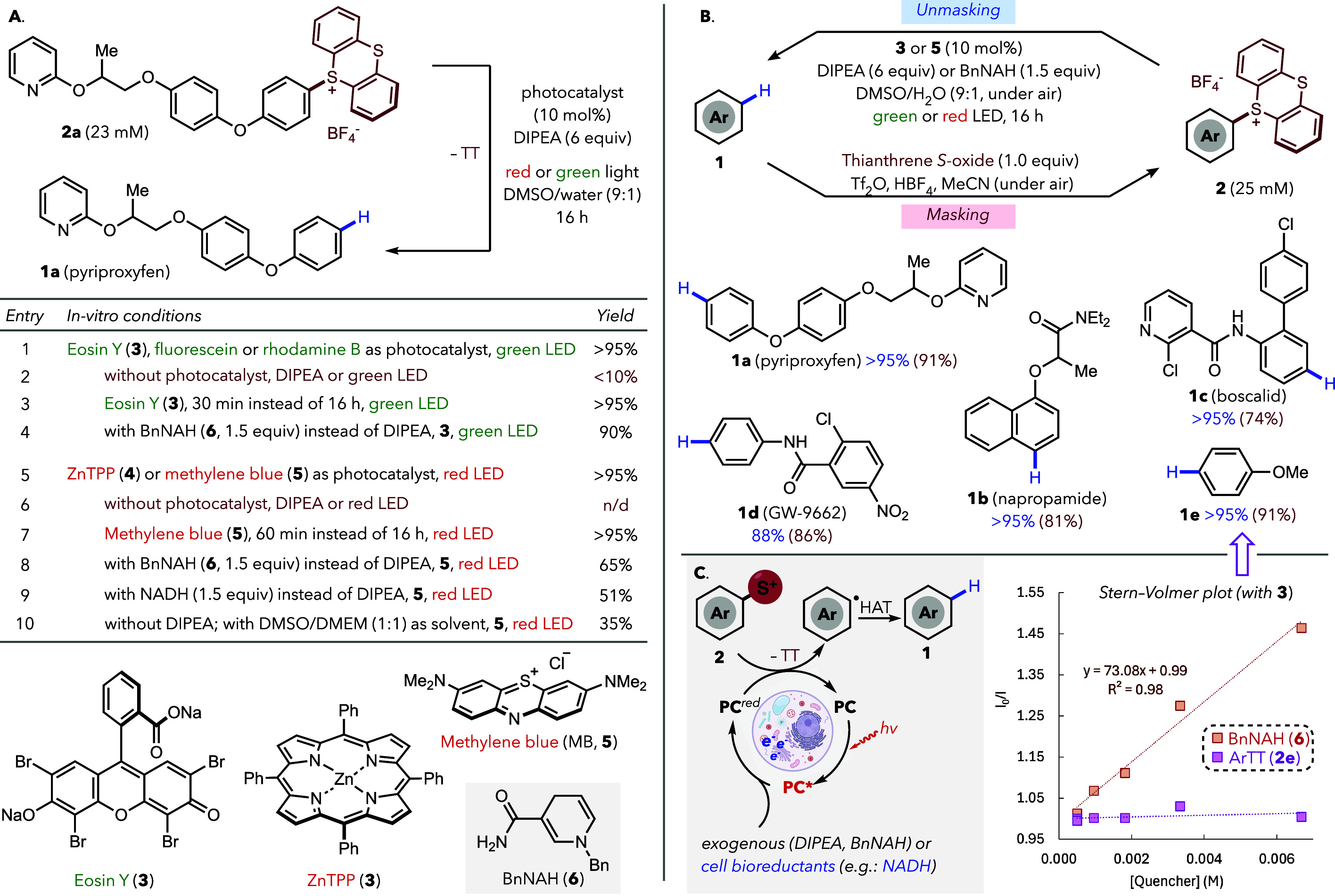
Low-energy light photoredox C–H unmasking. (**A**) *In vitro* development of the reaction. (**B**) Representative masked/unmasked bioactive molecules. ^1^H NMR yields in blue for unmasking (isolated yields for the masking step in red). (**C**) Mechanistic rationale and fluorescence quenching studies with Eosin Y (**3**). Irradiation with Kessil LEDs (525 or 660 nm), 44 W, Hepatochem PhotoRedOx Box.

The masking/unmasking sequence can be successfully applied to several bioactive molecules containing different functional groups (Figure 2B), including pyriproxyfen (pesticide), napropamide (herbicide), boscalid (fungicide), GW-9662 (anticancer), or lidocaine (anesthetic).^20,21^ Mechanistically, the reaction likely proceeds through a reductive-quenching photoredox manifold in which the excited photocatalyst accepts an electron from the (bio)­reductant and then promotes another SET to the masked substrate **2**, releasing thianthrene and an aryl radical. The latter is reduced by formal hydrogen-atom transfer (HAT) to furnish the parent compound **1** (Figure 2C, left). This is supported by Stern–Volmer studies with **Eosin Y (3)**, which showed quenched emission in the presence of reductant **6** (BnNAH), but was unaffected by thianthrenium salt **2e** (Figure 2C).^18,21^

Interestingly, the red-light photoredox conditions can also be used for synthetically relevant bond-forming reactions previously described under high-energy photocatalysis (Figure 3).^16^ For example, sulfonium **2f** can be coupled with *N*-methylpyrrole (**7**), using DIPEA and MB (**5**), to give the arylation-product **8** in good yield, after red-light irradiation in a 9:1 DMSO/water mixture, under air. Furthermore, we found that **2f** reacts with piperidine (**9**) with catalytic NiCl_2_·6H_2_O and ZnTPP (**4**), upon red-LED irradiation in DMA (under N_2_), delivering the C–N cross-coupling product **10** in 61% yield.^22^ To our knowledge, these constitute the first examples of red-light-promoted photoredox and metallaphotoredox catalytic reactions of aryl-sulfonium salts.^16,23^

**Figure 3:**
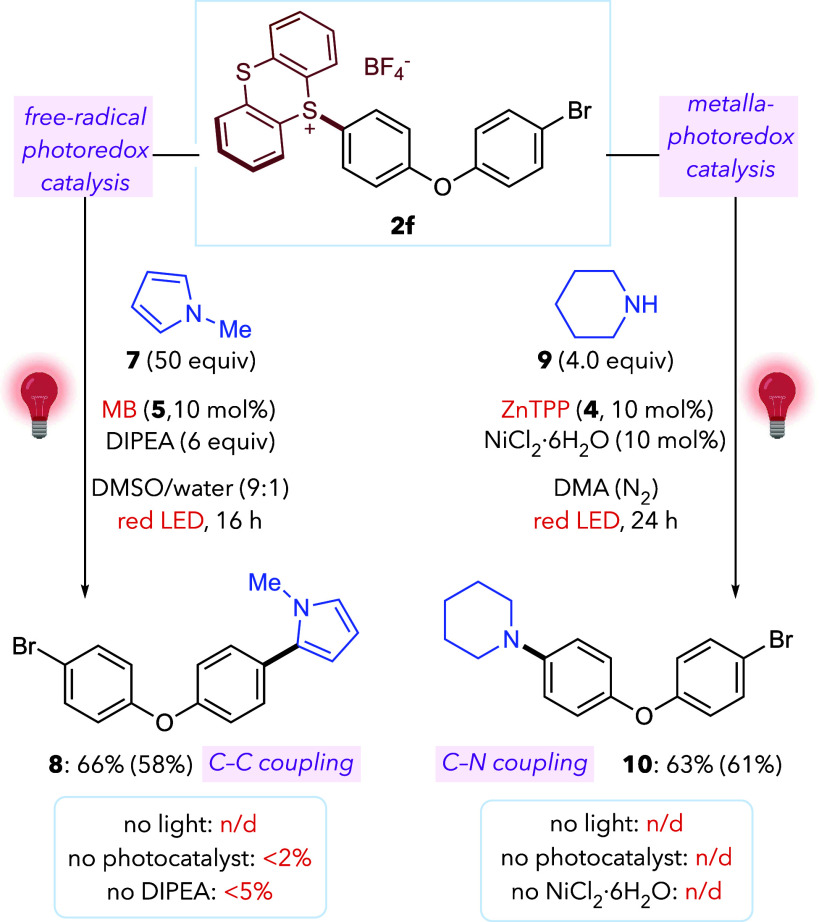
Red-light photoredox and metallaphotoredox activation of aryl-sulfonium salts for synthetic C–C and C–N bond formation. Yields by ^1^H NMR (isolated yields in parentheses).

With a view toward translating the above masking/unmasking strategy to challenging intracellular settings, we first examined the impact of the thianthrenium moiety on aqueous solubility and cellular permeability. To this end, we prepared salt **2g** (TPE–TT), a thianthrenated derivative of the otherwise water-insoluble dye TPE (**1g**).^24^ Thus, cultures of HeLa cells, *Bacillus thuringiensis* and *Staphylococcus aureus* were incubated for 15 min with either TPE (**1g**, 5–50 μM) or the sulfonium derivative TPE–TT (**2g**, 5–50 μM). After washing, fluorescence microscopy revealed significant intracellular accumulation and aggregation of AIE probe **2g** in all cases (Figure 4, A2, B2, C2). In contrast, insoluble free TPE **1g** did not enter cells, producing negligible intracellular emission (bacterial suspensions, Figure 4, B1, C1) with only extracellular deposits observed in adherent HeLa monolayers (Figure 4, A1). These results confirm that thianthrenation increases aqueous solubility and enables cellular uptake of compounds like TPE.

**Figure 4:**
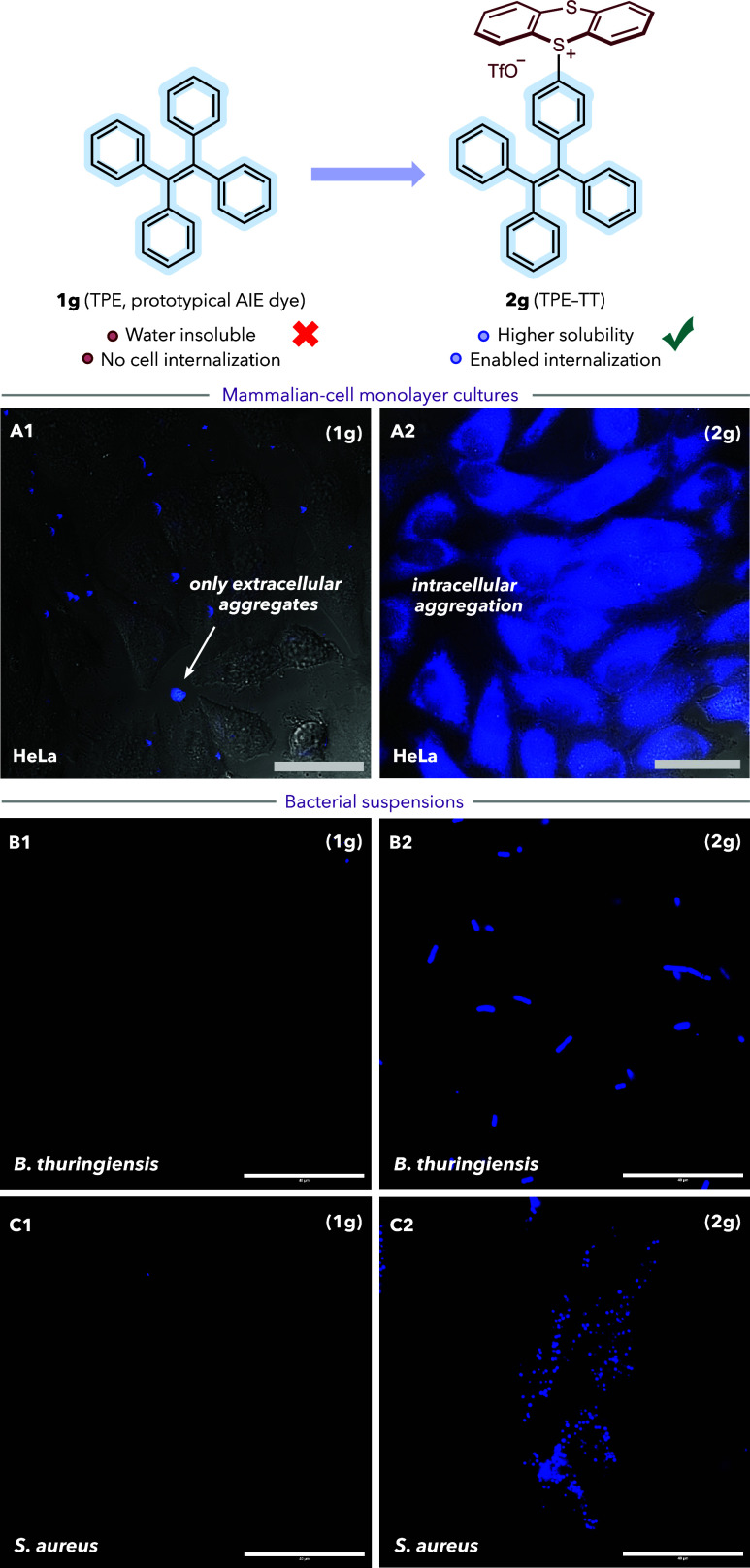
Internalization of TPE–TT in different organisms. Micrographies of cells after incubation with TPE (**1g**, A1–C1) or TPE–TT (**2g**, A2–C2) for 15 min, followed by washing (DMEM for HeLa cells and PBS for bacteria). Concentrations used: 5 μM for HeLa cells, and 50 μM for bacteria. λ_exc_ = 385 nm, λ_em_ = 430 nm. Scale bars = 40 μm.

Since TPE aggregation and insolubility may complicate quantification of its photoredox reactivity, we assessed the viability of an intracellular photoredox C–H unmasking with boscalid (**1c**), a SDHI fungicide that had been suggested to display some toxicity in hepatic human cell lines.^25^ Following the workflow outlined in Figure 5A,^10a^ HepG2 cell cultures were incubated with **2c** (100 μM) and Eosin Y (15 μM,) for 15 min in DMEM, followed by two washing steps with PBS to eliminate noninternalized material, and hence ensure the product is only formed inside cells. After adding fresh DMEM, culture plates were submitted to green-light irradiation for 60 min and finally, after washing with PBS, the cellular content was extracted with acetonitrile and analyzed by HPLC–MS (Figures 5A–B). Under these conditions, we observed the desired unmasked product **1c** (1.5·10^–2^ nmol/10^6^ cells, average of three biological replicates) in the intracellular extracts, along with unreacted masked precursor **2c** (34·10^–2^ nmol/10^6^ cells). No significant amounts of the unmasked product were detected in the extracellular milieu. Notably, we found a correlation between Eosin Y loading and amount of product formed (Figure 5B, right): indeed, with 25 μM of Eosin Y, the amount of boscalid increased up to 2.5·10^–2^ nmol/10^6^ cells. Whereas these conditions afforded the best intracellular reactivity, we also examined other variables, including photocatalyst, light source, and cell confluency (Figures S41–42). Importantly, control experiments without photocatalyst or light did not produce uncaged product, resulting only in the recovery of **2c**. MTT assays showed no significant toxicity for protected derivative **2c** toward HepG2 cells up to 200 μM (Figure 5C, left), even in the presence of Eosin Y and upon green-light irradiation, while the parent boscalid (**1c**) exhibited only marginally higher toxicity (Figure 5C, right).^25b^

**Figure 5:**
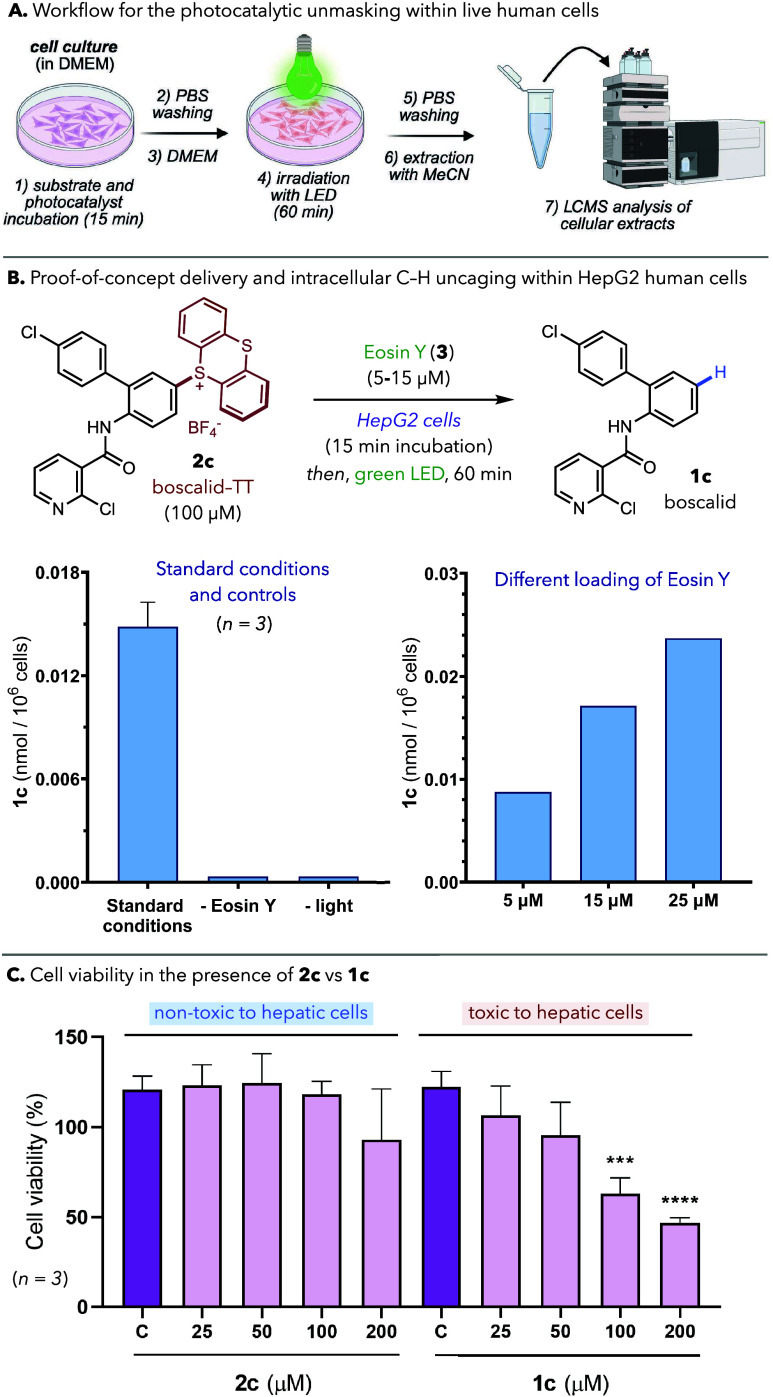
(**A**) Intracellular uncaging workflow: HepG2 cells were incubated with **2c** (100 μM) and **3** (15 μM) for 15 min, irradiated for 1 h with green light (Kessil 525 nm LED, 44 W, ∼15 cm above the plate, 60 mW cm^–1^) and analyzed by HPLC–MS. (**B**) Quantification of C–H uncaged product (boscalid, **1c**) in cell extracts by HPLC–MS. A very low-intensity peak was detected in both controls, yet its signal was near the noise threshold and outside the calibrated range. (**C**) Cytotoxicity profiles of **2c** and **1c** in HepG2 cells. Cells were treated for 24 h with increasing concentrations of **2c** and **1c**, or DMSO as a vehicle control. Cell viability was measured using MTT assay. Statistical significance of data was determined with one-way ANOVA statistical test (*p < 0.05; **p < 0.01; ***p < 0.001; ****p < 0.0001).

Overall, these results provide a proof-of-concept demonstration of in-cell abiotic chemistry involving carbon-radical intermediates, and suggest the feasibility of performing photocatalytic SET-based C–H unmasking reactions inside living cells.^17b,26^

Although the above data suggest that C–H thianthrenation can potentially influence biological activity, the low cytotoxicity of the parent boscalid hampers the observation of detectable changes after the C–H unmasking. Yet boscalid is a potent fungicide, and therefore we decided to check whether the masking/unmasking strategy could be used to control its antifungal properties. Therefore, 50 and 100 μg of boscalid (**1c**) or its masked derivative (**2c**) were added to blank antimicrobial paper discs, which were placed on potato dextrose agar (PDA) plates. After that, 10 mm plugs of 7-day-old *Botrytis cinerea* mycelium were placed in the center of the plates.^27^ Plates were incubated at 20 °C, and growth inhibition was evaluated after 10 days, revealing a sharp difference between the parent and the caged compound (Figure 6A). While free boscalid (**1c**) caused strong fungal growth inhibition (Figure 6, A3), the same quantities of the TT derivative **2c** (Figure 6, A1) behaved like solvent controls, showing essentially no inhibitory effect.

**Figure 6:**
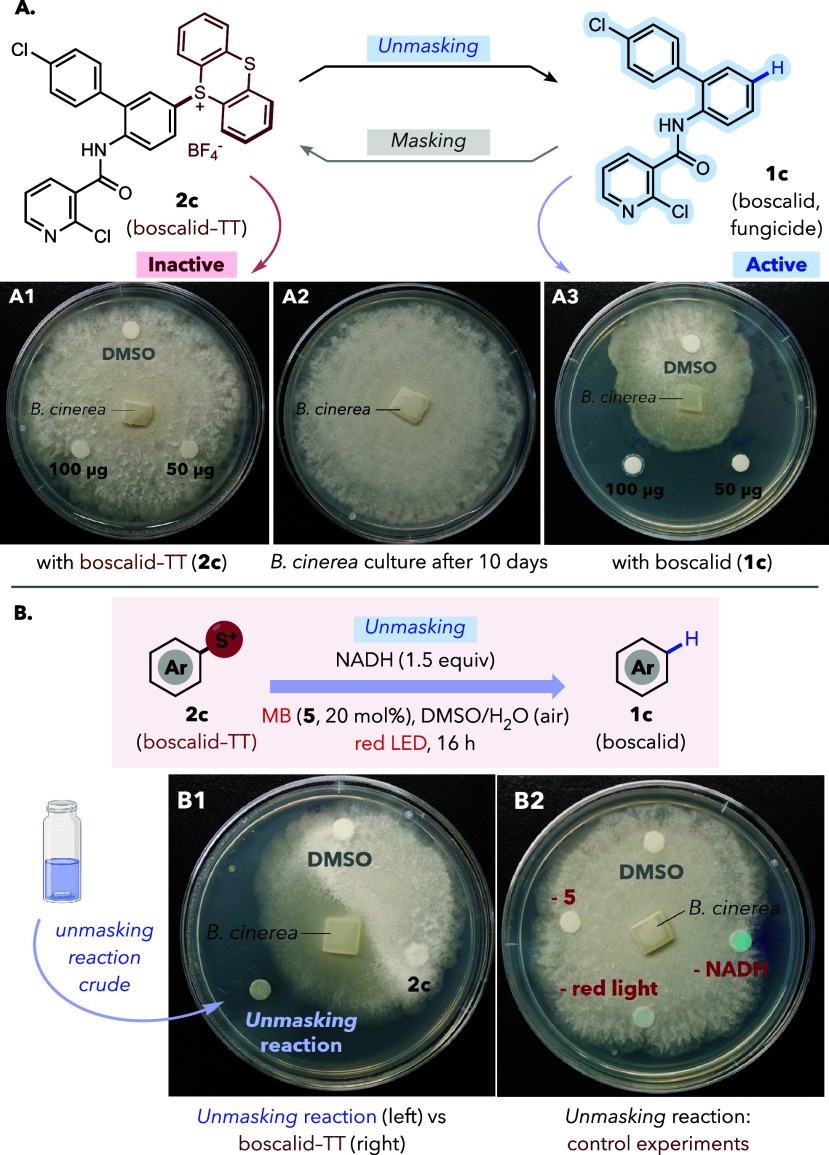
Antifungal activation of boscalid via C–H unmasking. (**A**) Disc assay comparing *Botrytis cinerea* growth inhibition upon exposure to **2c** vs **1c**. (**B**) Antifungal response after C–H unmasking of **2c** (left) and control experiments without light, NADH, or photocatalyst (right), indicated with the minus (−) sign al each spot.

After establishing this drastic difference, we exposed *B. cinerea* directly to crude mixtures of the unmasking reaction of protected boscalid **2c** (Figure 6, B1) at equivalent substrate loading (20 μL of a 10 mM solution of **2c** with 1.5 equiv of NADH in 9:1 DMSO/water, after 16 h of red-light irradiation with 20 mol% of **5**). This resulted in inhibition comparable to pure parent compound **1c**, whereas treatment with reaction mixtures resulting from control experiments (Figure 6, B2: without NADH, without photocatalyst **5** or without light) led to virtually no growth inhibition. Importantly, the same experiment with masked dummy arenes **2a** and **2e** instead of protected boscalid **2c** also gave no antifungal response. (Figure S48). These exploratory experiments support the feasibility of using our C–H masking/unmasking approach as a new uncaging strategy for the photoredox control of biological activities.

In summary, we introduce a new conceptual framework in the field of prodrug uncaging, based on the reversible masking of aromatic C–H bonds. The approach merges two powerful tools in modern organic chemistry: C–H functionalization (caging) and photoredox catalysis (uncaging), both largely unexplored in biological contexts. The deprotection, based on low-energy SET-based photoredox activation of aryl-thianthrenium salts, entails aryl radical intermediates, and can even be performed in the complex environment of a living cell. This represents a pioneering demonstration of the use of radical synthetic chemistry in living contexts. The masking strategy can be used to silence biological activity, as demonstrated for fungal-growth inhibition, which can be fully restored upon photoredox uncaging. Altogether, these findings establish a new proof-of-concept platform for light-controlled modulation of molecular function and should further foster the emerging field of photocatalytic bioorthogonal synthetic chemistry.

## Supplementary Material



## Notes

Additional experimental details, materials, methods and full characterization data (^1^H, ^13^C, ^19^F NMR, and HRMS) for all new compounds (PDF)
