# Supplementary material for: Photoredox Unmasking of Aromatic C–H Bonds in Living Environments Enabled by Thianthrenium Salts
Source: J Am Chem Soc. 2026 Feb 7;148(6):5946–52. doi: 10.1021/jacs.6c00530 (PMC12921698; doi:10.1021/jacs.6c00530)
Supplement: Supplementary file 1 [file ja6c00530_si_001.pdf]

Supporting Information

Photoredox Unmasking of Aromatic C–H Bonds in Living Environments Enabled by Thianthrenium Salts

Mauro Mato,[a] Adrián Rivas-Saborido,[a] Alba Casas-Pais,[a] María Tomás-Gamasa,[a,]* José L. Mascareñas[a,]*

[a] Centro Singular de Investigación en Química Biolóxica e Materiais Moleculares (CiQUS) and Departamento de Química Orgánica, Universidade de Santiago de Compostela, 15705, Santiago de Compostela (Spain).

* Email: maria.tomas@usc.es, joseluis.mascarenas@usc.es

Table of contents

1. General considerations 4

2. Light sources and photochemical set-up 5

2.1. Light sources (Kessil LED) 5

2.2. Photochemical reactor and set-up for in vitro chemical reactions 6

2.3. Photochemical reaction set-up for in cellulo experiments 8

3. C–H masking of aromatic compounds by thianthrenation 9

3.1. General procedure A for the C–H thianthrenation of aromatics 9

3.2. Characterization data for aryl thianthrenium salts 11

4. Photochemical C–H unmasking: screening and control experiments 20

4.1. Reaction development, screening and control experiments 21

4.2. In vitro biocompatibility experiments and use of bioreductants 23

5. Photocatalytic C–H unmasking of aromatic compounds in vitro 24

5.1. General procedure B for the in vitro unmasking of aryl thianthrenium salts 24

5.2. Experimental details and yield determination for the unmasking step 25

6. Photophysical properties 34

6.1. UV–Vis absorption spectroscopy 34

6.2. Emission spectroscopy (fluorescence) 39

7. Mechanistic and photophysical experiments 41

7.1. Overlap of absorption spectra and emission of the light sources 41

7.2. Absorption and AIE emission of protected and unprotected TPE 44

7.3. Fluorescence quenching studies 46

8. Synthetic applications of the red-light-based photoredox strategy 49

8.1. Red-light photoredox C–C coupling of aryl radicals 49

8.2. Red-light nickel-metallaphotoredox C–N coupling 51

9. General considerations for cellular experiments 53

10. Cell viability experiments 54

10.1. Analysis of the effect of free boscalid vs TT-protected boscalid on HepG2 cell viability 54

10.2. Analysis of the effect of different photocatalysts under light irradiation on the viability of mammalian cells 55

10.3. Effect of the uncaging reaction conditions on HepG2 cell viability 57

10.4. Effect of thianthrene in HepG2 cell viability 58

10.5. Effect of TPE–TT AIE probe in HeLa cell viability 59

11. Cell internalization and fluorescence microscopy with TPE–TT 60

11.1. Internalization of AIE probes in mammalian HeLa cells 60

11.2. Bacterial internalization of AIE probes 63

12. Delivery and photocatalytic uncaging in cell cultures 66

12.1. General procedure C for the intracellular reactions in HepG2 cells 66

12.2. Quantification of caged and uncaged boscalid from HepG2 cells extracts 67

12.3. Intracellular photoredox uncaging reaction results 68

13. Fungal culture experiments 75

13.1. Experiments of exposure of *B. cinerea* cultures to masked and free boscalid 75

13.2. Experiments of exposure of *B. cinerea* cultures to unmasking reactions 76

14. NMR spectra 81

15. Supplementary references 101

1. General considerations

Unless stated otherwise, all reactions were conducted under air in HPLC-grade solvents. The water used in the reactions was deionized and purified on a Millipore Milli-Q® Integral system. Dry solvents (if needed) were directly purchased from Sigma–Aldrich and used without further purification. Unless otherwise noted, all reagents were obtained from commercial suppliers and used without further purification. Phosphate buffered saline (PBS, pH 7.4) was prepared following standard procedures. Dulbecco′s Modified Eagle's Medium (GibcoTM DMEM) was purchased from ThermoFisher Scientific. HeLa cell lysates were obtained from 2 days cultured HeLa cells: after two washings with PBS, cells were scraped from the well, sonicated and diluted with PBS to reach the indicated concentration.

^{1}H and ^{13}C NMR data were recorded in $CDCl_3$, using a Varian Mercury 300 MHz or Bruker AVIII 500 MHz spectrometer. (at 298–300 K, unless stated otherwise). ^{1}H and ^{13}C chemical shifts (δ) are reported in ppm relative to the solvent residual peaks as internal reference. For ^{1}H NMR, the following residual proton peaks of the deuterated solvents were used: $CDCl_3$, $\delta_H(CHCl_3)$7.26. For ^{13}C NMR: $CDCl_3$, δ77.16. ^{13}C spectra were acquired with broadband ^{1}H decoupling unless mentioned otherwise. Coupling constants (*J*) are provided in Hz, and ^{1}H-NMR multiplicities are reported as follows: chemical shift (δ ppm), integration, multiplicity (s = singlet, d = doublet, t = triplet, q = quartet, dd = double doublet, td = triple doublet, m = multiplet, br = broad). NMR spectra were analyzed using MestreNova NMR data processing software. GC–MS analysis was performed on a 8890 GC System with a 5977B GC/MSD (CI) from Agilent. High-resolution mass spectra (HRMS) were acquired using electrospray ionization (ESI) in a Bruker microTOF (time-of-flight analyzer) instrument in FIA mode (flow-injection analysis).

UV–Vis absorption spectra were recorded on a Jasco V-770 spectrometer using either disposable plastic cuvettes (2x10 mm) or quartz cuvettes (10x10 mm). Emission spectra were recorded on an Edinburgh FS5 Spectrofluorometer using quartz fluorescence cuvettes (10x10 mm). Unless stated otherwise, all spectroscopic studies were performed under air, mimicking the actual reaction conditions.

Thin-layer chromatography (TLC) was performed on pre-coated Merck 60 silica gel F_{254} plates. TLC plates were visualized by observation under UV light and/or staining with either phosphomolybdic acid solution or potassium permanganate solution, followed by heat. Chromatographic purifications were performed by flash column chromatography on silica gel (Merck Geduran® Si 60, 40–63 μm) or by preparative TLC using PLC Silica gel 60 F_{254}, 1 mm, 20x20 cm (Analtech).

HPLC–MS analysis was carried out using an Agilent 1260 Infinity II system connected to an Agilent Technologies 6120 Quadrupole LC-MS, with a Phenomenex Luna-C18 reverse-phase column (250x10 mm).

Unless stated otherwise, EY, Eosin Y-Na_2 or EY-Na_2 all refer to Eosin Y disodium salt (**3**), and ZnTPP refers to zinc(II) tetraphenylporphyrin (**4**) and MB refers to methylene blue (**5**). TT stands for thianthrene, and "ArTT" stands for aryl thianthrenium salt.

2. Light sources and photochemical set-up

2.1. Light sources (Kessil LED)

Kessil PR160L

Blue, green and red-light irradiation was performed using standardized 456, 525 and 660 nm LED (respectively) PR160L lamps purchased from Kessil, in combination with the PhotoRedOx Duo^{TM} photochemical reactor from HepatoChem (see next section for details). Unless stated otherwise, the lamps were used at full intensity (100%). Intensity maps and more details can be found at the Kessil website: https://kessil.com/products/science_PR160L.php

The emission profile of the three light sources was recorded with a MK350S Premium Handheld Spectrometer from UPRtek, sitting around 20 cm in front of the Kessil LED light at 25% intensity (Figure S1).

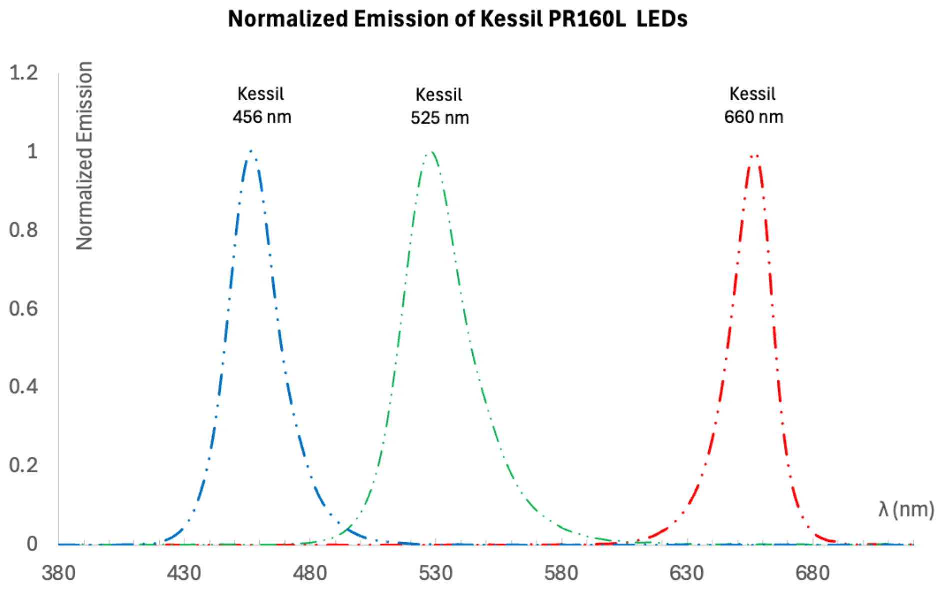

Figure S1. Recorded normalized emission of the Kessil LED light sources employed in this study.

2.2. Photochemical reactor and set-up for in vitro chemical reactions

PhotoRedOx Duo™

In vitro photochemical reactions were performed using a PhotoRedOx Duo™ reactor from HepatoChem, attaching a single Kessil PR160L lamp of the appropriate wavelength (see previous section for details) at full intensity (100%) to the chamber where the reactions were run (sample holders). If both chambers/holders were employed at the same time, one Kessil lamp of the same color was attached to each of them. The photoreactor is based on a simple mirror set up which directs the light towards the reaction vials (sample holders) and is equipped with an electrical ventilation system to keep the temperature of the chambers below 30 ºC.

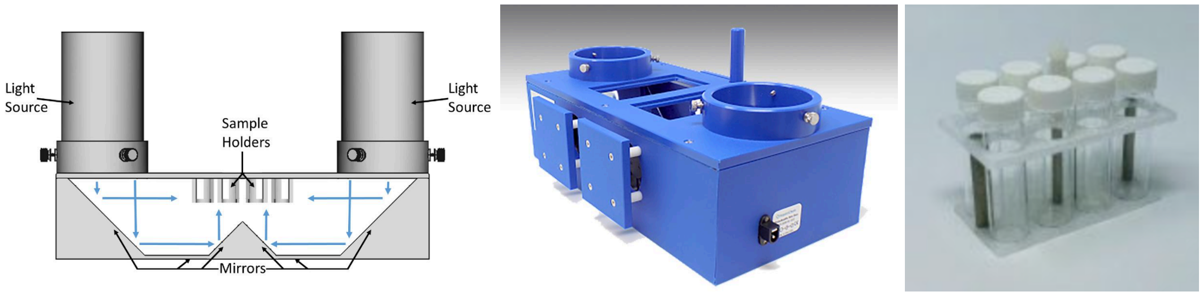

Figure S2. Schematic representation (left), picture of the photoreactor (center) and sample holder for 8 mL vials (right) provided by HepatoChem.

A detailed description of the photoreactor can be found at the HepatoChem website:

https://hepatochem.com/photoreactors-leds-accessories/photoredox-duo/

The photoreactor was installed on top of a stirring plate in order to stir the reaction solutions using Teflon-coated magnetic stirring bars. The reactions were set up in 6 mL screw-cap vials under ambient atmosphere, with no special precautions taken to exclude air. To ensure efficient irradiation of the solutions, the volume of the vials was always kept between 1.0 and 4.0 mL. A maximum of 4 reaction vials (placed on the row of the sample holder closer the light source) were irradiated at the same time.

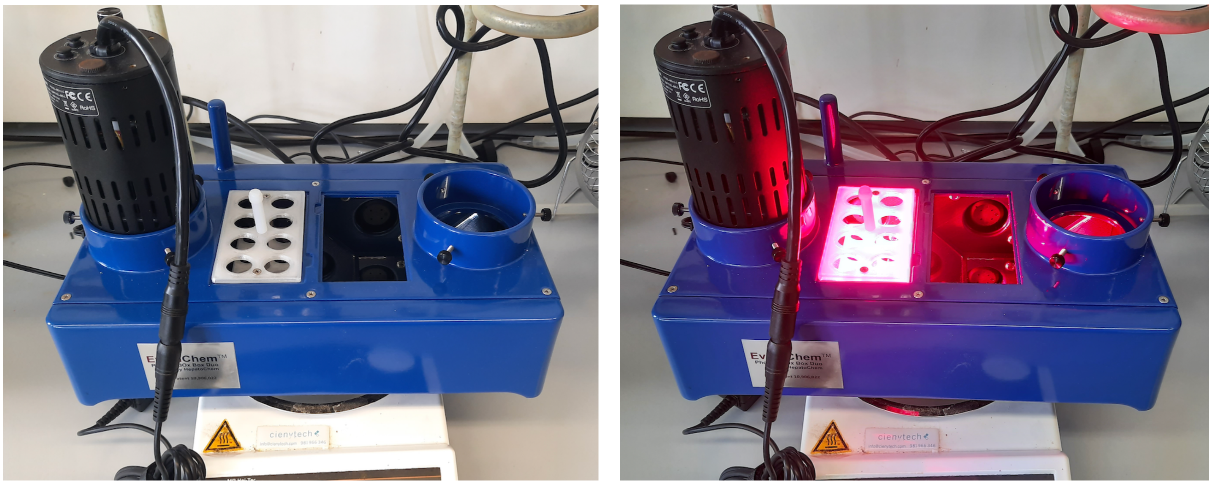

Figure S3. Pictures of the photochemical set up with one operative chamber (light off or on).

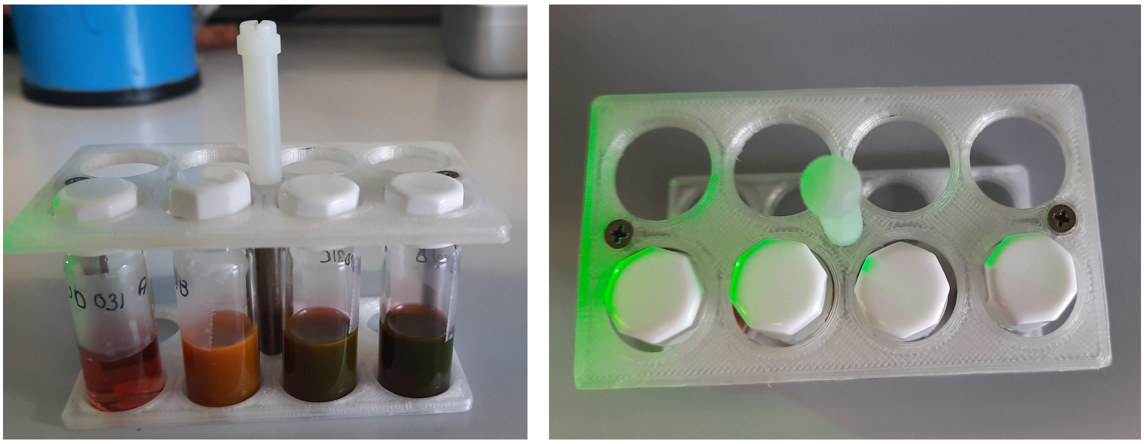

Figure S4. Pictures of the reaction vials and sample holder that is located in the chamber; front (left) or top view (right).

2.3. Photochemical reaction set-up for in cellulo experiments

In cellulo photochemical experiments were performed using a single Kessil PR160L LED lamp (appropriate wavelength, see previous section) positioned 15 cm above the cell culture plates. The lamp operated at full power (100%) under continuous light exposure. Temperature was regulated with one or two external axial fans placed near the irradiation area to maintain airflow and keep the samples below 30 °C during the experiments.

Cell cultures were exposed to light in standard 24-well glass-bottom plates or 100 mm dishes under ambient atmospheric conditions. Unless otherwise noted, the working volume per well ranged between 300 and 500 µL in the 24-well glass-bottom plates and between 3 and 5 mL in the 100 mm dishes to ensure effective light penetration. Only two 100 mm dishes were irradiated simultaneously to minimize shading effects and ensure uniform light distribution.

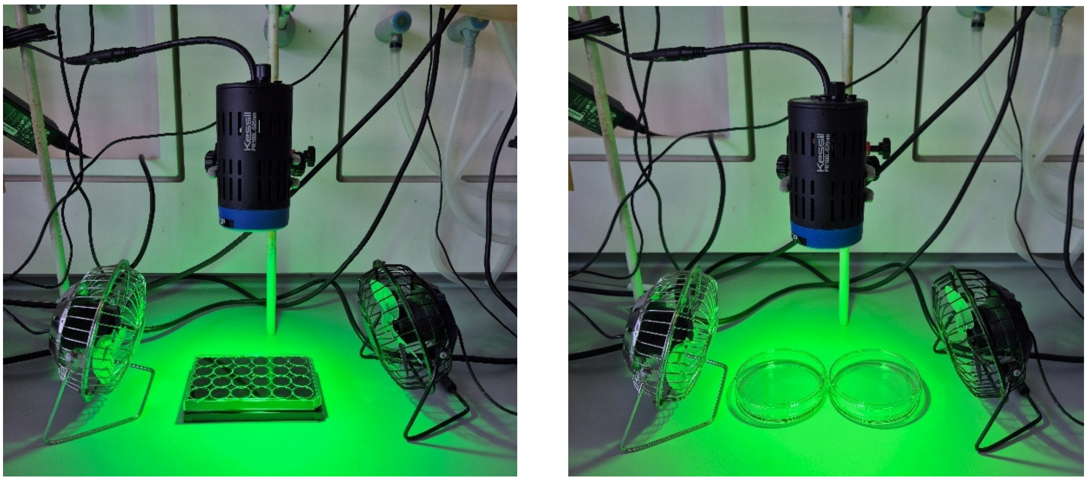

Figure S5. Pictures of the photochemical set up for in cellulo experiments in 24-well glass-bottom (left) or 100 mm dishes (right).

3. C–H masking of aromatic compounds by thianthrenation

3.1. General procedure A for the C–H thianthrenation of aromatics

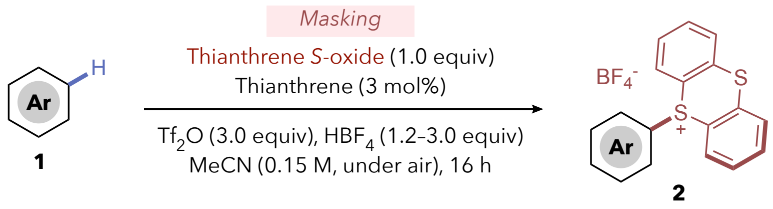

Following typical procedures for the synthesis of aryl thianthrenium salts,[1,2,3] a round-bottom flask of the appropriate size (equipped with a Teflon-coated stirring bar) was charged under air atmosphere with the corresponding arene **1** (1.0 equiv), thianthrene-*S*-oxide (1.0 equiv) and thianthrene (0.03 equiv). This was followed by the addition of HPLC-grade acetonitrile (0.15–0.20 M) and, after ca. 1 min of stirring, $HBF_4 \cdot Et_2O$ (1.2–3.0 equiv, depending on the substrate) was added in a single portion via syringe. The resulting stirred mixture was cooled down to 0 ºC using a water/ice bath, before Tf_2O (3.0 equiv) was added dropwise via syringe, resulting in an immediate color change to dark purple. The resulting solution was stirred at 0 ºC for 30 min, before removing the cooling bath and further stirring at room temperature (ca. 22 ºC) for 16 h.

After this time, the mixture was diluted with DCM (ca. 30 mL/mmol **1**), water (ca. 15 mL/mmol **1**) and subsequently quenched with a saturated aqueous Na_2CO_3 solution (ca. 15 mL/mmol **1**). The biphasic mixture was poured into a separatory funnel and shaken energetically before the two phases were separated. The organic fraction was washed again once with saturated aqueous Na_2CO_3 solution (ca. 30 mL/mmol **1**), then twice with 5%(w/w) aqueous $NaBF_4$ solution (ca. 2x 20 mL/mmol **1**) and twice with water (2x ca. 20 mL/mmol **1**). Then, the organic fraction was dried over anhydrous $MgSO_4$, filtered, and concentrated in vacuum. The resulting product was purified by flash column chromatography in silica gel, using gradients of DCM/MeOH as eluent. The obtained products were redissolved in DCM (ca. 2 mL/mmol **1**) and subsequently crashed out by adding Et_2O (ca. 15 mL/mmol **1**). The resulting suspension was filtered out and washed with another portion of Et_2O, and further dried in high vacuum, giving the corresponding pure product **2**.

The following sulfonylated arenes were prepared and tested for the deprotection strategy studied:

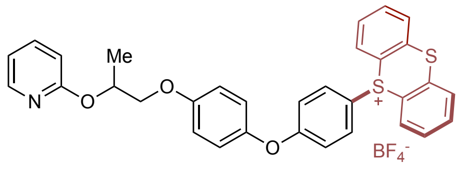

2a (from pyriproxyfen)

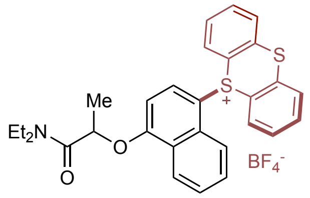

2b (from napropamide)

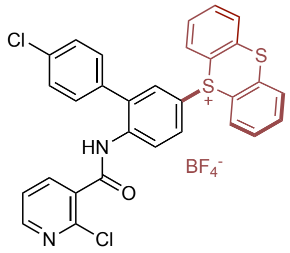

2c (from boscalid)

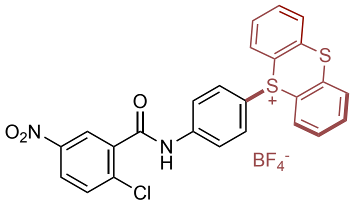

2d (from GW-9662)

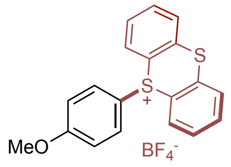

2e (from anisole)

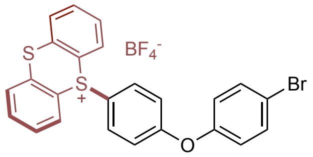

2f (from 1-bromo-4-phenoxybenzene)

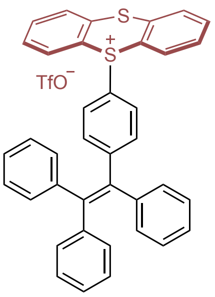

2g (from TPE)

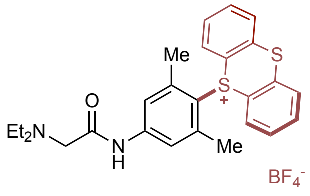

SI-1 (from lidocaine)

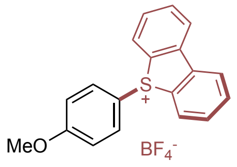

SI-2 (from anisole)

Notes:

- Many of the aryl thianthrenium salts can be obtained in a relatively pure form even before column chromatography, after simply crashing out the product with Et_2O and washing. This approach might be enough for applications in photoredox catalysis. However, we found that chromatography is the only way to fully and reliably remove any leftover unreacted starting arene, which is mandatory for studying the unmasking reactions described in this work. Thus, column chromatography was performed for all the substrates employed.
- If upon addition of Et_2O to a concentrated solution of a product **2** in DCM (after column chromatography) a free-flowing solid is not obtained, sonication of the mixture in an ultrasound bath often helps to crash out the thianthrenium salt product. Then, solvent removal either by evaporation or filtration, leads to the pure thianthrenium salt as a solid.
- While some sulfonylated compounds **2** were already known in the literature,[1] for the sake of practicality, exact experimental details and NMR data are reported in the following pages. For reference, ^1H NMR data of commercial samples of unmasked products is also included.
- Exceptionally, compound **2g** was prepared from the corresponding parent boronic acid following a reported procedure (see end of this section for details).[5]

3.2. Characterization data for aryl thianthrenium salts

Pyriproxyfen derived thianthrenium salt (2a)

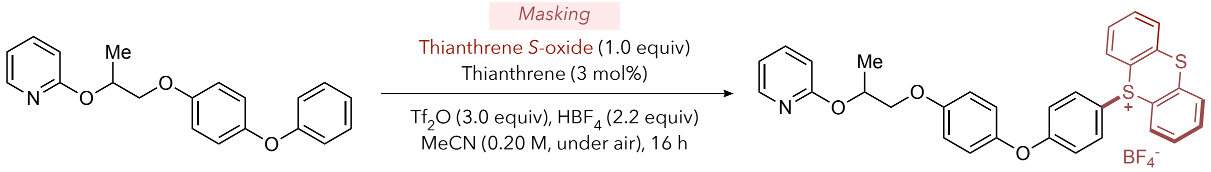

Following General Procedure A, the title product was obtained from pyriproxyfen (160 mg, 0.50 mmol, 1.0 equiv), thianthrene-*S*-oxide (116 mg, 0.50 mmol, 1.0 equiv), thianthrene (TT, 3.2 mg, 0.015 mmol, 3 mol%), tetrafluoroboric acid diethyl ether complex ($HBF_4{\cdot}Et_2O$, 178 mg, 0.15 mL, 1.1 mmol 2.2 equiv) and trifluoromethanesulfonic anhydride (Tf_2O, 423 mg, 0.25 mL, 1.5 mmol 3.0 equiv) in MeCN (2.5 mL, 0.20 M). Purification by flash column chromatography in silica gel was performed using DCM/MeOH as solvent (gradient from 99:1 to 9:1). This gave 283 mg (91%) of the title product as a white foam.

***R**$_f$* (9:1 DCM/MeOH) = 0.4

^{1}H NMR (300 MHz, $CDCl_3$) δ 8.47 (d, *J* = 7.7 Hz, 2H), 8.11 (dd, *J* = 5.0, 2.0 Hz, 1H), 7.76 (ddt, *J* = 20.7, 7.5, 3.7 Hz, 6H), 7.60 – 7.49 (m, 1H), 7.17 (d, *J* = 8.9 Hz, 2H), 6.87 (dd, *J* = 10.6, 5.3 Hz, 7H), 6.70 (d, *J* = 8.3 Hz, 1H), 5.55 (q, *J* = 5.8 Hz, 1H), 4.15 (dd, *J* = 9.9, 5.3 Hz, 1H), 4.04 (dd, *J* = 9.9, 4.8 Hz, 1H), 1.44 (d, *J* = 6.4 Hz, 3H).

^{13}C NMR (75 MHz, $CDCl_3$) δ 163.16, 163.10, 156.54, 147.55, 146.76, 138.75, 136.19, 134.92, 134.82, 130.41, 130.30, 130.20, 121.71, 119.16, 118.50, 116.84, 116.21, 115.17, 111.63, 71.08, 69.14, 16.96.

^{19}F NMR (282 MHz, $CDCl_3$) δ -151.04, -151.09.

HRMS (ESI+): calculated for $C_{32}H_{26}NO_3S_2$ $[M–BF_4]^+$: 536.1349; found: 536.1342.

Unmasked pyriproxyfen (**1a**):

^{1}H NMR (300 MHz, $CDCl_3$) δ 8.15 (ddd, *J* = 5.1, 2.0, 0.8 Hz, 1H), 7.57 (ddd, *J* = 8.4, 7.1, 2.0 Hz, 1H), 7.33 – 7.26 (m, 2H), 7.07 – 7.00 (m, 1H), 6.98 – 6.90 (m, 6H), 6.86 (ddd, *J* = 7.1, 5.1, 1.0 Hz, 1H), 6.75 (dt, *J* = 8.4, 0.9 Hz, 1H), 5.59 (dt, *J* = 6.6, 5.5 Hz, 1H), 4.19 (dd, *J* = 9.9, 5.3 Hz, 1H), 4.07 (dd, *J* = 9.9, 4.9 Hz, 1H), 1.48 (d, *J* = 6.4 Hz, 3H).

Napropamide derived thianthrenium salt (2b)

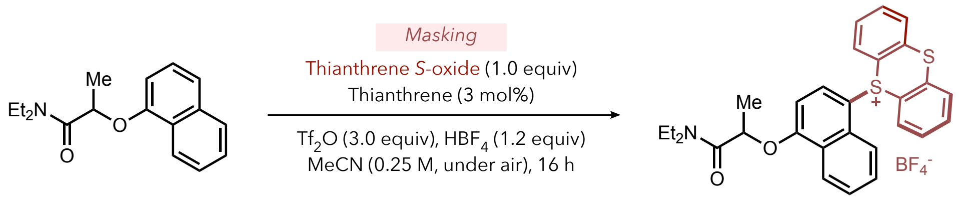

Following General Procedure A, the title product was obtained from napropamide (271 mg, 1.0 mmol, 1.0 equiv), thianthrene-*S*-oxide (232 mg, 1.0 mmol, 1.0 equiv), thianthrene (TT, 6.4 mg, 0.030 mmol, 3 mol%), tetrafluoroboric acid diethyl ether complex ($HBF_4 \cdot Et_2O$, 194 mg, 0.16 mL, 1.2 mmol 1.2 equiv) and trifluoromethanesulfonic anhydride (Tf_2O, 846 mg, 0.51 mL, 3.0 mmol 3.0 equiv) in MeCN (4 mL, 0.20 M). Purification by flash column chromatography in silica gel was performed using DCM/MeOH as solvent (gradient from 99:1 to 97:3). This gave 463 mg (81%) of the title product as a pale beige foam.

R_f (95:5 DCM/MeOH) = 0.3

^{1}H NMR (300 MHz, $CDCl_3$) δ 8.50 (d, *J* = 8.4 Hz, 1H), 8.18 (d, *J* = 8.4 Hz, 1H), 7.99 (dd, *J* = 12.1, 8.0 Hz, 2H), 7.81 (dd, *J* = 7.9, 5.4 Hz, 2H), 7.68 (td, *J* = 8.2, 4.7 Hz, 4H), 7.60 – 7.50 (m, 3H), 6.92 (d, *J* = 8.6 Hz, 1H), 5.35 (q, *J* = 6.6 Hz, 1H), 3.49 (q, *J* = 6.7 Hz, 2H), 3.36 (h, *J* = 6.8 Hz, 2H), 1.78 – 1.72 (d, *J* = 6.7 Hz, 3H), 1.18 (t, *J* = 7.1 Hz, 3H), 1.09 (t, *J* = 7.1 Hz, 3H).

^{13}C NMR (75 MHz, $CDCl_3$) δ 168.76, 160.04, 135.66, 135.19, 135.09, 134.02, 133.97, 132.31, 130.96, 130.46, 130.42, 130.30, 130.18, 130.14, 127.41, 127.33, 124.66, 122.39, 120.31, 120.18, 106.40, 104.60, 73.38, 41.40, 40.39, 18.12, 14.33, 12.66.

^{19}F NMR (282 MHz, $CDCl_3$) δ -151.90, -151.96.

HRMS (ESI+): calculated for $C_{29}H_{28}NO_2S_2$ $[M–BF_4]^+$: 486.1556; found: 486.1550.

Unmasked napropamide (**1b**):

^{1}H NMR (300 MHz, $CDCl_3$) δ 8.34 – 8.25 (m, 1H), 7.84 – 7.75 (m, 1H), 7.47 (tdd, *J* = 8.7, 6.7, 4.7 Hz, 3H), 7.33 (td, *J* = 8.0, 1.2 Hz, 1H), 6.83 (d, *J* = 7.6 Hz, 1H), 5.18 – 5.05 (m, 1H), 3.64 – 3.34 (m, 4H), 1.73 (dd, *J* = 6.7, 1.2 Hz, 3H), 1.11 (td, *J* = 7.1, 1.1 Hz, 3H), 1.00 (td, *J* = 7.1, 1.2 Hz, 3H).

Boscalid derived thianthrenium salt (2c)

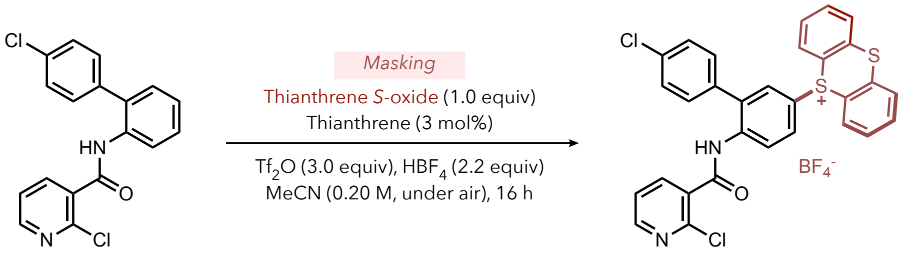

Following General Procedure A, the title product was obtained from boscalid (515 mg, 1.5 mmol, 1.0 equiv), thianthrene-*S*-oxide (348 mg, 1.5 mmol, 1.0 equiv), thianthrene (TT, 10 mg, 0.045 mmol, 3 mol%), tetrafluoroboric acid diethyl ether complex ($HBF_4 \cdot Et_2O$, 534 mg, 0.45 mL, 3.3 mmol 2.2 equiv) and trifluoromethanesulfonic anhydride (Tf_2O, 1.27 g, 0.76 mL, 4.5 mmol 3.0 equiv) in MeCN (6.0 mL, 0.25 M). Purification by flash column chromatography in silica gel was performed using DCM/MeOH as solvent (gradient from 95:5 to 9:1). This gave 720 mg (74%) of the title product as a white solid.

$\boldsymbol{R_f}$ (9:1 DCM/MeOH) = 0.3

^{1}H NMR ^{1}H NMR (300 MHz, CD_3CN) δ 8.50 (s, 1H), 8.46 – 8.38 (m, 3H), 8.30 (d, *J* = 8.9 Hz, 1H), 8.05 – 7.99 (m, 2H), 7.93 (td, *J* = 7.6, 1.5 Hz, 2H), 7.88 – 7.81 (m, 3H), 7.50 – 7.45 (m, 2H), 7.41 (dd, *J* = 7.6, 4.8 Hz, 1H), 7.34 – 7.28 (m, 2H), 7.24 (dd, *J* = 8.9, 2.6 Hz, 1H), 7.13 (d, *J* = 2.6 Hz, 1H).

^{13}C NMR (75 MHz, CD_3CN) δ 164.17, 151.08, 146.68, 139.11, 138.18, 136.60, 135.45, 135.19, 134.98, 134.53, 131.82, 131.00, 130.82, 130.12, 130.03, 129.15, 128.44, 124.87, 122.92, 119.29, 118.69.

^{19}F NMR (282 MHz, CD_3CN) δ -151.71, -151.76.

HRMS (ESI+): calculated for $C_{30}H_{19}Cl_2N_2OS_2$ $[M–BF_4]^+$: 557.0310; found: 557.0314.

Unmasked boscalid (**1c**):

^{1}H NMR (300 MHz, $CDCl_3$) δ 8.43 (dd, *J* = 4.7, 2.0 Hz, 1H), 8.40 (d, *J* = 8.0 Hz, 1H), 8.16 – 8.07 (m, 2H), 7.48 – 7.43 (m, 1H), 7.43 – 7.40 (m, 2H), 7.36 – 7.30 (m, 3H), 7.27 – 7.24 (m, 2H).

GW-9662 derived thianthrenium salt (2d)

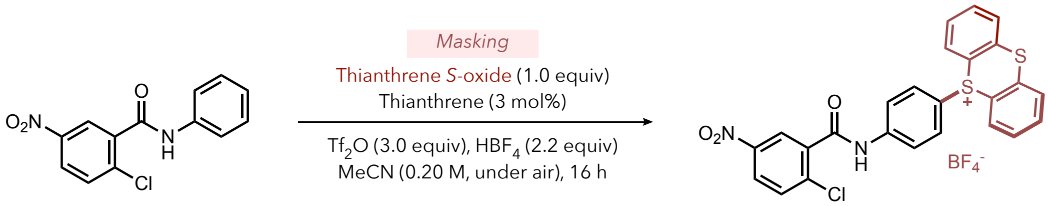

Following General Procedure A, the title product was obtained from GW-9662 (138 mg, 0.50 mmol, 1.0 equiv), thianthrene-*S*-oxide (116 mg, 0.50 mmol, 1.0 equiv), thianthrene (TT, 3.2 mg, 0.015 mmol, 3 mol%), tetrafluoroboric acid diethyl ether complex ($HBF_4 \cdot Et_2O$, 178 mg, 0.15 mL, 1.1 mmol 2.2 equiv) and trifluoromethanesulfonic anhydride (Tf_2O, 423 mg, 0.25 mL, 1.5 mmol 3.0 equiv) in MeCN (2.0 mL, 0.25 M). Purification by flash column chromatography in silica gel was performed using DCM/MeOH as solvent (gradient from 98:2 to 9:1). This gave 250 mg (86%) of the title product as a white foam.

$\boldsymbol{R_f}$ (9:1 DCM/MeOH) = 0.4

^{1}H NMR (300 MHz, CD_3CN) δ 9.25 (s, 1H), 8.44 – 8.38 (m, 2H), 8.30 (dd, *J* = 8.9, 2.8 Hz, 1H), 8.05 – 7.73 (m, 10H), 7.26 – 7.17 (m, 2H).

^{13}C NMR (75 MHz, CD_3CN) δ 163.53, 146.60, 142.50, 137.63, 136.50, 135.12, 134.84, 131.47, 130.75, 130.54, 129.98, 129.46, 126.20, 124.12, 121.16, 118.86, 117.65.

^{19}F NMR (282 MHz, CD_3CN) δ -151.56, -151.62.

HRMS (ESI+): calculated for $C_{25}H_{16}ClN_2O_3S_2$ $[M–BF_4]^+$: 491.0285; found: 491.0279.

Unmasked GW-9662 (**1d**):

^{1}H NMR (300 MHz, $CDCl_3$) δ 8.60 (d, *J* = 2.7 Hz, 1H), 8.26 (dd, *J* = 8.8, 2.7 Hz, 1H), 7.94 (s, 1H), 7.70 – 7.61 (m, 3H), 7.42 (t, *J* = 7.8 Hz, 2H), 7.24 (t, *J* = 7.4 Hz, 1H).

Anisole derived thianthrenium salt (2e)

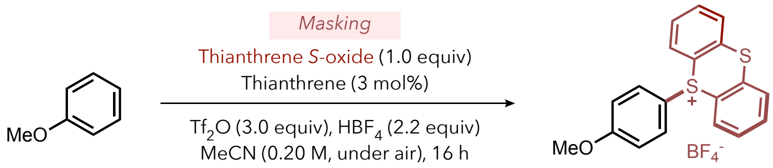

Following General Procedure A, the title product was obtained from anisole (216 mg, 2.0 mmol, 1.0 equiv), thianthrene-*S*-oxide (465 mg, 2.0 mmol, 1.0 equiv), thianthrene (TT, 13 mg, 0.060 mmol, 3 mol%), tetrafluoroboric acid diethyl ether complex ($HBF_4 \cdot Et_2O$, 389 mg, 0.33 mL, 2.4 mmol 1.2 equiv) and trifluoromethanesulfonic anhydride (Tf_2O, 0.85 g, 0.51 mL, 3.0 mmol 1.5 equiv) in MeCN (7.0 mL, 0.30 M). Purification by flash column chromatography in silica gel was performed using DCM/MeOH as solvent (gradient from 99:1 to 95:5). This gave 0.75 g (91%) of the title product as a white solid.

R*f* (95:5 DCM/MeOH) = 0.4

^{1}H NMR (300 MHz, $CDCl_3$) δ 8.54 – 8.43 (m, 2H), 7.85 – 7.70 (m, 6H), 7.30 – 7.25 (m, 2H), 6.99 – 6.92 (m, 2H), 3.80 (s, 3H).

^{13}C NMR (75 MHz, $CDCl_3$) δ 163.66, 136.04, 134.81, 134.61, 130.58, 130.28, 130.09, 119.63, 116.50, 113.60, 55.89.

^{19}F NMR (282 MHz, $CDCl_3$) δ -151.27, -151.32.

HRMS (ESI+): calculated for $C_{19}H_{15}OS_2$ $[M–BF_4]^+$: 323.0559; found: 323.0554.

Unmasked anisole (**1e**):

^{1}H NMR (300 MHz, $CDCl_3$) δ 7.33 – 7.27 (m, 2H), 6.98 – 6.89 (m, 3H), 3.81 (s, 3H).

1-Bromo-4-phenoxybenzene derived thianthrenium salt (2f)

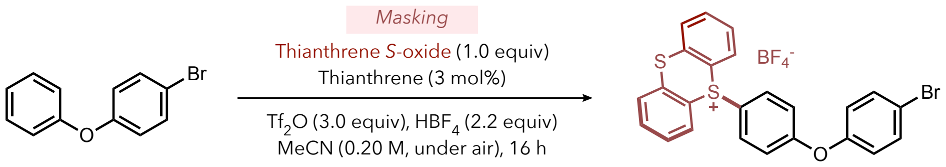

Following General Procedure A, the title product was obtained from 1-bromo-4-phenoxybenzene (374 mg, 1.50 mmol, 1.0 equiv), thianthrene-*S*-oxide (348 mg, 1.50 mmol, 1.0 equiv), thianthrene (TT, 10 mg, 0.045 mmol, 3 mol%), tetrafluoroboric acid diethyl ether complex ($HBF_4{\cdot}Et_2O$, 267 mg, 0.23 mL, 1.7 mmol 1.1 equiv) and trifluoromethanesulfonic anhydride (Tf_2O, 635 mg, 0.38 mL, 2.3 mmol 1.5 equiv) in MeCN (5.0 mL, 0.30 M). Purification by flash column chromatography in silica gel was performed using DCM/MeOH as solvent (gradient from 98:2 to 9:1). This gave 720 mg (87%) of the title product as a white foam.

R_f (95:5 DCM/MeOH) = 0.4

^{1}H NMR (300 MHz, $CDCl_3$) δ 8.61 – 8.51 (m, 2H), 7.88 – 7.72 (m, 6H), 7.53 – 7.41 (m, 2H), 7.27 – 7.21 (m, 2H), 7.01 – 6.94 (m, 2H), 6.93 – 6.81 (m, 2H).

^{13}C NMR (75 MHz, $CDCl_3$) δ 161.73, 153.55, 136.26, 135.21, 134.83, 133.33, 130.53, 130.37, 130.18, 122.14, 119.23, 119.15, 118.28, 116.46.

^{19}F NMR (282 MHz, $CDCl_3$) δ -150.93, -150.98.

HRMS (ESI+): calculated for $C_{24}H_{16}{}^{79}BrOS_2$ $[M–BF_4]^+$: 462.9820; found: 462.9817.

Unmasked 1-bromo-4-phenoxybenzene (**1f**):

^{1}H NMR (300 MHz, $CDCl_3$) δ 7.47 – 7.39 (m, 2H), 7.39 – 7.31 (m, 2H), 7.16 – 7.10 (m, 1H), 7.04 – 6.97 (m, 2H), 6.92 – 6.85 (m, 2H).

Lidocaine derived thianthrenium salt (SI-1)

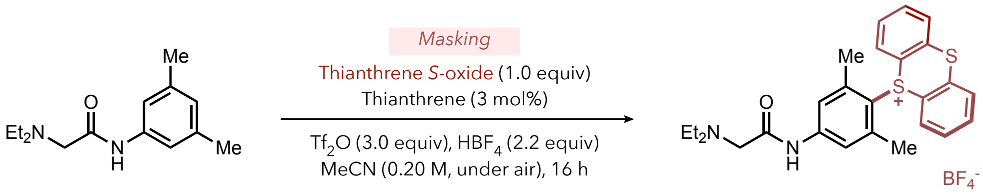

Following General Procedure A, the title product was obtained from lidocaine (117 mg, 0.50 mmol, 1.0 equiv), thianthrene-*S*-oxide (116 mg, 0.50 mmol, 1.0 equiv), thianthrene (TT, 3.2 mg, 0.015 mmol, 3 mol%), tetrafluoroboric acid diethyl ether complex ($HBF_4 \cdot Et_2O$, 243 mg, 0.20 mL, 1.5 mmol 3.0 equiv) and trifluoromethanesulfonic anhydride (Tf_2O, 423 mg, 0.25 mL, 1.5 mmol 3.0 equiv) in MeCN (2.0 mL, 0.25 M). Purification by flash column chromatography in silica gel was performed using DCM/MeOH as solvent (slow gradient from 98:2 to 9:1). This gave 120 mg (45%) of the title product as a pale beige solid.

***R**$_f$* (9:1 DCM/MeOH) = 0.4

^{1}H NMR (300 MHz, CD_3CN) δ 9.13 (s, 1H), 8.39 (dd, *J* = 7.9, 1.5 Hz, 2H), 8.02 (dd, *J* = 7.9, 1.5 Hz, 2H), 7.94 (td, *J* = 7.6, 1.5 Hz, 2H), 7.89 – 7.83 (m, 2H), 6.92 (s, 2H), 2.73 – 2.66 (m, 4H), 2.15 (s, 6H), 1.12 (t, *J* = 7.2 Hz, 6H).

^{13}C NMR (75 MHz, CD_3CN) δ 170.16, 140.21, 138.89, 136.58, 135.14, 135.01, 130.80, 130.03, 126.88, 118.47, 57.05, 48.54, 17.99, 11.58.

^{19}F NMR (282 MHz, CD_3CN) δ -151.71, -151.76.

HRMS (ESI+): calculated for $C_{26}H_{29}N_2OS_2$ $[M–BF_4]^+$: 449.1716; found: 449.1708.

Unmasked lidocaine:

^{1}H NMR (300 MHz, $CDCl_3$) δ 8.90 (s, 1H), 7.09 (s, 2H), 3.22 (s, 2H), 2.69 (q, *J* = 7.1 Hz, 4H), 2.24 (s, 6H), 1.14 (t, *J* = 7.1 Hz, 6H).

4-Anisole derived dibenzothiophenium salt (SI-2)

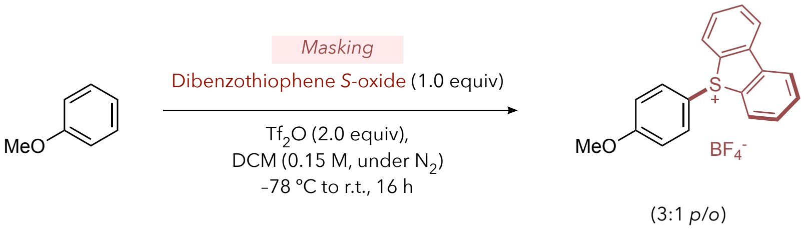

Following a modified version of General Procedure A, at lower temperature, in a different solvent and using dibenzothiophene *S*-oxide as reagent.

A 50 mL two-necked round-bottom flask equipped with a Teflon-coated stirring bar was charged with anisole (0.18 g, 0.18 mL, 1.7 mmol, 1.1 equiv) and dibenzothiophene-*S*-oxide (0.30 g, 1.5 mmol, 1.0 equiv). The flask was evacuated and refilled with nitrogen three times, followed by the addition of dry DCM (10 mL, 0.15 M). After the resulting mixture was cooled down to –78 ºC in an acetone/dry ice bath, Tf_2O (0.86 g, 0.51 mL, 3.0 mmol, 2.0 equiv) was added dropwise via syringe, resulting in an immediate color change to dark blue. The resulting solution was stirred at –78 ºC for 30 min, before removing the cooling bath and further stirring at room temperature (ca. 22 ºC) for 16 h.

After this time, the mixture was diluted with DCM (ca. 30 mL/mmol **1**), water (ca. 15 mL/mmol **1**) and subsequently quenched with a saturated aqueous Na_2CO_3 solution (ca. 15 mL/mmol **1**). The biphasic mixture was poured into a separatory funnel and shaken energetically before the two phases were separated. The organic fraction was washed again once with saturated aqueous Na_2CO_3 solution (ca. 30 mL/mmol **1**), then twice with 5%(w/w) aqueous $NaBF_4$ solution (ca. 2x 20 mL/mmol **1**) and twice with water (2x ca. 20 mL/mmol **1**). Then, the organic fraction was dried over anhydrous $MgSO_4$, filtered, and concentrated in vacuum. The resulting product was purified by flash column chromatography in silica gel, using a gradient between 99:1 to 90:10 DCM/MeOH as eluent. The product was redissolved in DCM (ca. 2 mL/mmol **1**) and subsequently crashed out by adding Et_2O (ca. 15 mL/mmol **1**). The resulting suspension was filtered out and washed with another portion of Et_2O, and further dried in high vacuum, giving the title product (0.41 g, 72%), as a 3:1 mixture of *para* and *ortho* regioisomers. Characterization data matched the ones reported for this compound in the literature.[4]

^{1}H NMR (300 MHz, $CDCl_3$) δ 8.24 – 8.20 (m, 2H), 8.04 (d, *J* = 8.1 Hz, 2H), 7.87 – 7.82 (m, 2H), 7.64 – 7.54 (m, 4H), 7.02 – 6.97 (m, 2H), 3.84 (s, 3H). The ratio of regioisomers was determined by integration of the MeO signal at 3.93 ppm belonging to the *ortho* isomer.

^{19}F NMR (282 MHz, $CDCl_3$) δ -151.19, -151.25.

HRMS (ESI+): calculated for $C_{19}H_{15}OS$ $[M–BF_4]^+$: 291.0838; found: 291.0833.

Preparation and characterization of [TPE–TT]OTf (2g)

The title compound was prepared from the corresponding boronic acid adapting a procedure reported for other substrates.[5] While the direct C–H thianthrenation of 1,1,2,2-tetraphenylethylene can be achieved through General Procedure A to some extent, practical considerations such as selectivity and purification issues led us to choosing this procedure for the synthesis of this particular substrate.

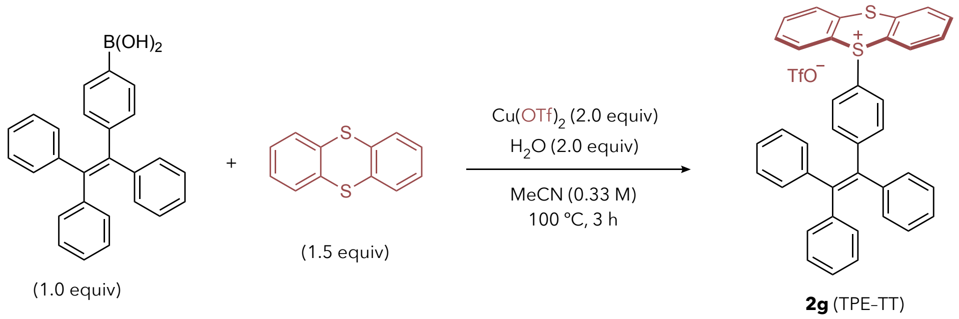

2g (TPE-TT)

1,1,2,2-Tetraphenylethene derived thianthrenium salt [TPE–TT]OTf (**2g**)

A 10 mL pressure Schlenk tube was charged with (4-(1,2,2-triphenylvinyl)phenyl)boronic acid (188 mg, 0.50 mmol, 1.0 equiv), thianthrene (TT, 162 mg, 0.75 mmol, 1.5 equiv) and $Cu(OTf)_2$ (362 mg, 1.0 mmol, 2.0 equiv). The atmosphere was evacuated and refilled with N_2 three times, before dry MeCN (1.5 mL, 0.33 M) and H_2O (MiliQ-grade, 18 µL, 2.0 equiv) were added. Then, the tube was sealed with its cap and the mixture was stirred in an oil bath at 100 ºC during 3 h (the mixture evolves from a suspension to a dark homogeneous mixture over the course of the reaction). After this time, the mixture was cooled down to room temperature and poured into 20 mL of aqueous ammonia (ca. 30% aqueous NH_4OH). The product was extracted twice with DCM (2x 20 mL) in a separatory funnel. The combined organic fractions were washed twice with water (2x 20 mL), dried over anhydrous Na_2SO_4, filtered and concentrated in vacuum. Then, the product was purified by flash column chromatography in silica gel, using a gradient of DCM/MeOH from 99:1 to 95:5 as eluent. After removal of the solvent, the product was redissolved in ca. 1 mL of DCM, and crashed out by adding 5 mL of Et_2O. Further removal of the solvent gave the title product in pure form as a pale beige foam (240 mg, 71%).

^{1}H NMR (300 MHz, CD_3CN) δ 8.30 (dd, *J* = 7.9, 1.4 Hz, 2H), 7.96 (dd, *J* = 7.8, 1.5 Hz, 2H), 7.87 (td, *J* = 7.6, 1.5 Hz, 2H), 7.78 (td, *J* = 7.6, 1.5 Hz, 2H), 7.16 – 7.08 (m, 11H), 6.99 (tt, *J* = 7.0, 3.4 Hz, 4H), 6.94 – 6.89 (m, 2H), 6.84 (d, *J* = 8.5 Hz, 2H).

^{13}C NMR (75 MHz, CD_3CN) δ 148.95, 143.71, 142.77, 142.57, 142.31, 138.67, 136.63, 135.11, 134.95, 132.79, 130.86, 130.83, 130.69, 130.63, 129.89, 128.02, 127.94, 127.82, 127.53, 127.09, 127.04, 121.06, 118.88.

^{19}F NMR (282 MHz, CD_3CN) δ -79.24.

HRMS (ESI+): calculated for $C_{38}H_{27}S_2$ $[M–OTf]^+$: 547.1549; found: 547.1546.

4. Photochemical C–H unmasking: screening and control experiments

Unless stated otherwise, all reactions described in Section 4 were carried out using the following standard protocol, using sulfonylated pyriproxyfen **2a** as model substrate, applying the corresponding deviations or specific conditions particular to each experiment.

Unless stated otherwise, yields were always determined by ^1H NMR adding 1 equiv of 1,3,5-trimethoxybenzene as internal standard.

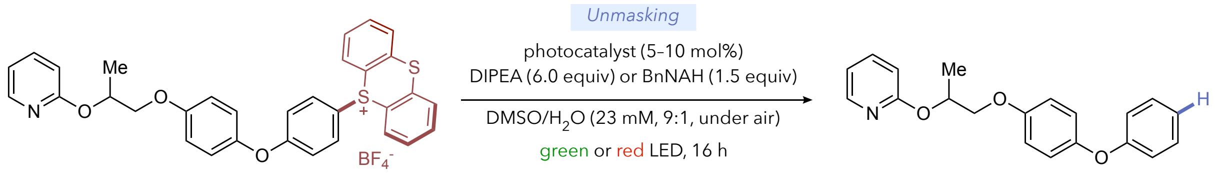

Under air atmosphere, a 6 mL screw-cap glass vial equipped with a Teflon-coated magnetic stirring bar was charged with protected pyriproxyfen **2a** (1.0 equiv, 0.023 mmol unless stated otherwise), the corresponding reductant (generally DIPEA; BnNAH or NADH) and photocatalyst (generally 5–10 mol%). Everything was dissolved in a 9:1 mixture of DMSO/water (generally 1.0 mL, 23 mM). Then, the vial was closed with the corresponding cap and loaded into the photoredox set up (Kessil lamp and Hepatochem PhotoRedOx Duo™, see Section 2 for details). Then, the vial was irradiated at the corresponding wavelength (generally 525 nm or 660 nm) upon stirring over 16 h. After this time, irradiation was switched off and 1.0 equiv of 1,3,5-trimethoxybenzene (internal standard) was added as a solution in 2.0 mL of EtOAc, followed by the addition of ca. 3 mL of water. After shaking, the organic phase was extracted with a glass Pasteur pipette, dried with anhydrous Na_2SO_4, filtered, evaporated in high vacuum and submitted to crude ^1H NMR analysis.

The following photocatalysts were employed throughout the screening. Acronyms used in the screening tables are also included below.

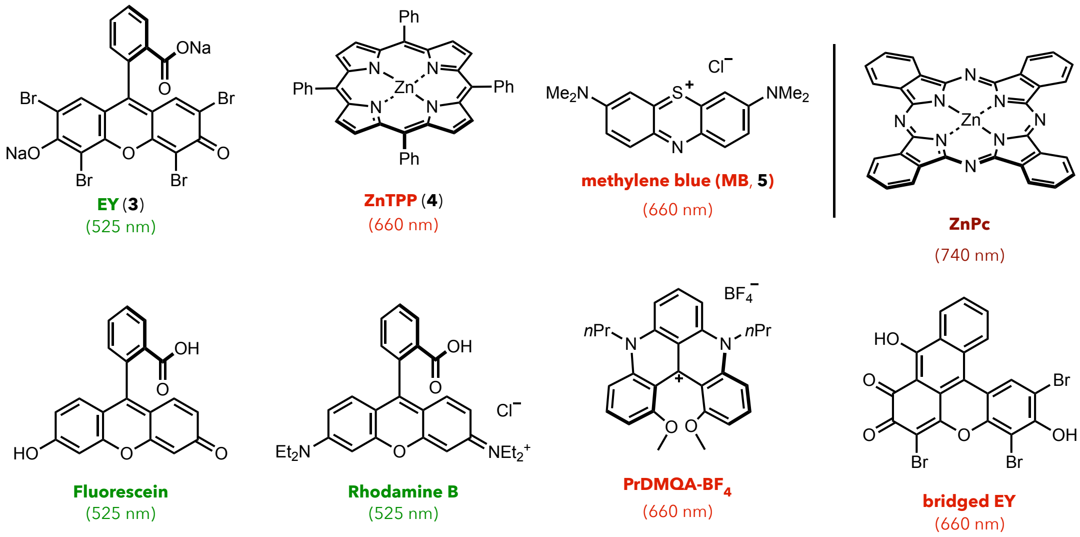

Figure S6. List of screened photocatalysts.

Bridged Eosin Y was prepared according to a literature procedure.[6] The rest of the photocatalysts were purchased from commercial sources and used as received.

4.1. Reaction development, screening and control experiments

Table S1. Initial development of the deprotection. Yields were always determined by ^{1}H NMR adding 1 equiv of 1,3,5-trimethoxybenzene as internal standard. TT = thianthrene.

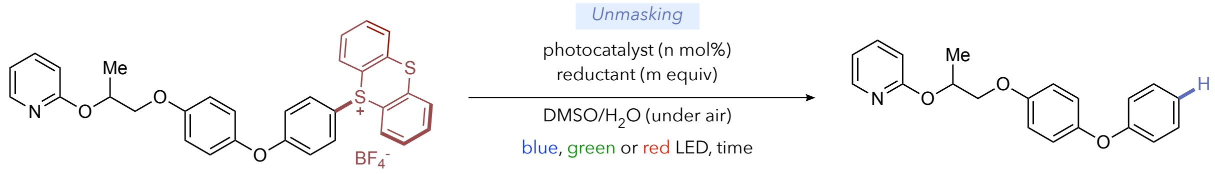

2a - **TT** **1a**

Entry	Conditions / Deviations	Recov. **2a**	**1a**	**TT**
1	Ir(cFCF$_3$ppy)$_2$(dtbpy)PF$_6$ (5 mol%) blue LED, DIPEA (6 equiv) DMSO/H$_2$O 9:1 (46 mM), 6 h	<5%	>95%	>95%
2	with BnNAH (1.5 equiv) instead of DIPEA	<5%	>95%	>95%
3	**EY** (5mol%), green LED, DIPEA (6 equiv) DMSO/H$_2$O 9:1 (46 mM), 6 h	<1%	>95%	>95%
4	with BnNAH (1.5 equiv) instead of DIPEA	<5%	>95%	>95%

Table S2. Control experiments with green and red light. Yields were always determined by ^{1}H NMR adding 1 equiv of 1,3,5-trimethoxybenzene as internal standard. TT = thianthrene.

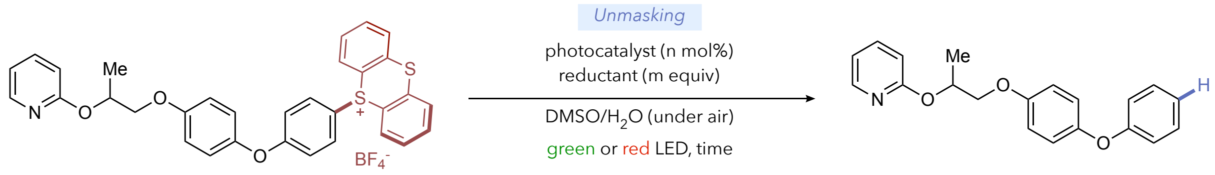

2a - **TT** **1a**

Entry	Conditions / Deviations	Recov. **2a**	**1a**	**TT**
1	**EY** (5 mol%), green LED, DIPEA (6 equiv) DMSO/H$_2$O 9:1 (23 mM), 16 h	<1%	>90%	>90%
2	with BnNAH (1.5 equiv) instead of DIPEA	<5%	>90%	>90%
3	without photocatalyst (green LED + DIPEA)	>75%	7%	6%
4	without DIPEA (green LED + **EY**)	>75%	<3%	<3%
5	without light (DIPEA + **EY**)	>75%	<1%	<1%
6	**ZnTPP** (10 mol%), red LED, DIPEA (6 equiv) DMSO/H$_2$O 9:1 (46 mM), 16 h	<1%	95%	93%
7	with BnNAH (1.5 equiv) instead of DIPEA	21%	70%	65%
8	without photocatalyst (red LED + DIPEA)	>75%	<1%	<1%
9	without light (DIPEA + **ZnTPP**)	>65%	<1%	<1%

Note: Due to partial loss of thianthrenium salt **2a** in the aqueous phase after a single EtOAc extraction, recoveries of the protected starting material are rarely >75% and are thus expressed with a ">" sign.

Table S3. Control experiments with green and red light. Yields were always determined by ^{1}H NMR adding 1 equiv of 1,3,5-trimethoxybenzene as internal standard. TT = thianthrene.

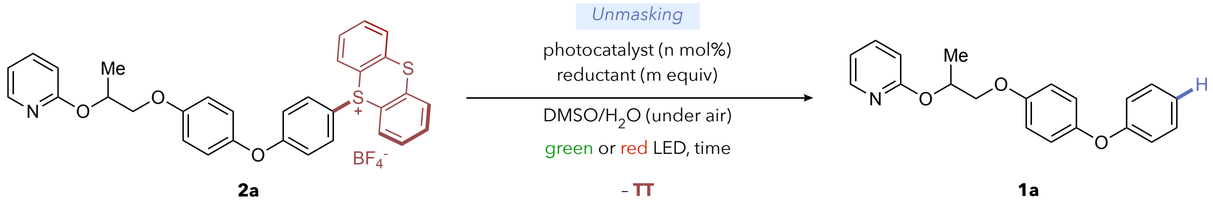

Entry	Conditions / Deviations	Recov. **2a**	**1a**	**TT**
1	**ZnTPP** (10 mol%), 660 nm LED, DIPEA (6 equiv) DMSO/H_2O 9:1 (23 mM), 16 h	<1%	95%	93%
2	with **PrDMQA-BF$_4$** instead of ZnTPP	<1%	92%	88%
3	with **methylene blue** instead of ZnTPP	<1%	93%	91%
4	with **bridged Eosin Y** instead of ZnTPP	<1%	62%	64%
5	with **ZnPc** instead of ZnTPP, 740 nm NIR lamp	69%	28%	30%
6	with **fluorescein** instead of ZnTPP, 525 nm	<1%	>95%	93%
7	with **rhodamine B** instead of ZnTPP, 525 nm	<1%	>95%	92%
8	with **Eosin Y Na$_2$** instead of ZnTPP, 525 nm	<1%	>90%	>90%

The photocatalytic process was found to be very robust, performing very well across a wide variety of low-energy light-absorbing photocatalysts. Notably, 740 nm NIR light could also be used, giving the product in a moderate yield.

We also evaluated shorter reaction times, revealing that the reaction is complete in roughly 30–60 min using either the red-light or green-light systems.

Table S4. Evaluating shorter reaction times. Yields were always determined by ^{1}H NMR adding 1 equiv of 1,3,5-trimethoxybenzene as internal standard. TT = thianthrene.

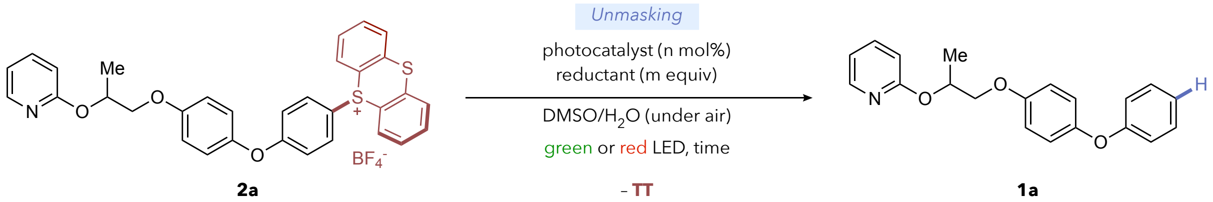

Entry	Conditions / Deviations	Recov. **2a**	**1a**	**TT**
1	**methylene blue** (10 mol%), 660 nm LED, DIPEA (6 equiv) DMSO/H_2O 9:1 (23 mM), 24 h	<1%	>90%	>90%
2	60 min instead of 16 h	<1%	86%	88%
3	30 min instead of 16 h	10%	79%	80%
1	**Eosin Y Na$_2$** (10 mol%), 525 nm LED, DIPEA (6 equiv) DMSO/H_2O 9:1 (23 mM), 24 h	<1%	>95%	>95%
2	60 min instead of 16 h	<1%	>95%	>95%
3	30 min instead of 16 h	<1%	>95%	>95%

4.2. In vitro biocompatibility experiments and use of bioreductants

We found that the red-light-promoted deprotection reaction with methylene blue was still operative employing cell-endogenous bioreductants.

Table S5. Experiments using bioreductants. Yields were always determined by ^{1}H NMR adding 1 equiv of 1,3,5-trimethoxybenzene as internal standard. TT = thianthrene.

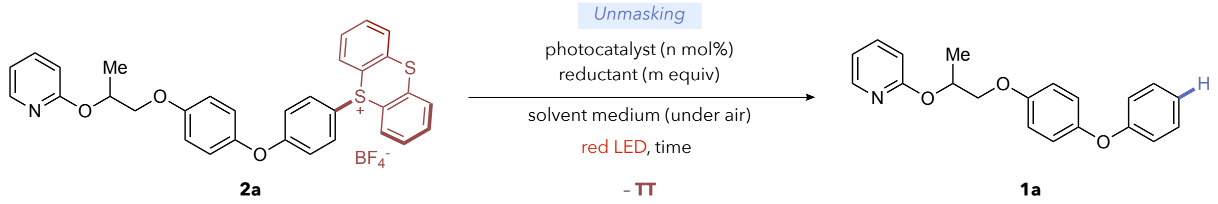

Entry	Conditions / Deviations	Recov. **2a**	**1a**	**TT**
	**MB** (10 mol%), 660 nm LED, bio-reductant (1.5 equiv) DMSO/H_2O, 8:2 (10 mM, 1 mL total vol.), **4 h**			
1	NADH (1.5 equiv)	>30%	51%	48%
2	GSH, reduced glutathione (1.5 equiv)	>51%	4%	3%
3	sodium ascorbate (1.5 equiv)	>50%	10%	12%
4	1 mL of DMEM (no additional reductant)	>60%	17%	18%

Furthermore, we found that the wide variety of biomolecules contained in DMEM (Dulbecco's Modified Eagle's Medium, a widely used basal medium for supporting the growth of mammalian cells) was able to promote the unmasking reaction at moderately high rates. This prompted us to study the reactions in living systems using the reducing potential contained in cell reductants as source of electrons.

Table S6. Experiments using DMEM instead of additional reductants. Yields were always determined by ^{1}H NMR adding 1 equiv of 1,3,5-trimethoxybenzene as internal standard. TT = thianthrene. n/dm = not determined. n/d = not detected.

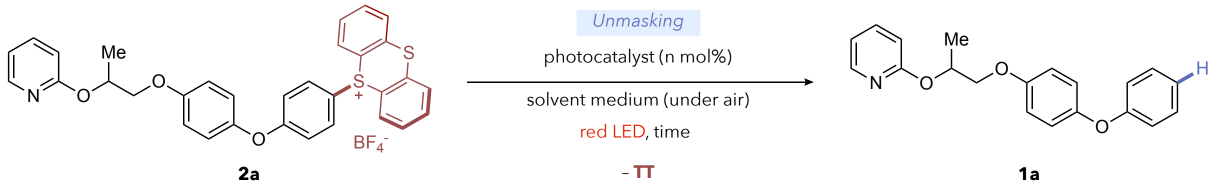

Entry	Conditions / Deviations	Recov. **2a**	**1a**	**TT**
	**MB** or **ZnTPP** (10 mol%), 660 nm LED or **dark**, **16 h** DMSO/dmem 1:1 (5 mM SM, 2 mL total vol.)			
1	**MB** + 660 nm LED	n/dm	35%	35%
2	**MB** + **dark**	n/dm	n/d	n/d
3	**ZnTPP** + 660 nm LED	n/dm	7%	7%
4	**ZnTPP** + **dark**	n/dm	n/d	n/d
5	no catalyst + 660 nm LED	n/dm	n/d	n/d
6	no catalyst + **dark**	n/dm	n/d	n/d

5. Photocatalytic C–H unmasking of aromatic compounds in vitro

5.1. General procedure B for the in vitro unmasking of aryl thianthrenium salts

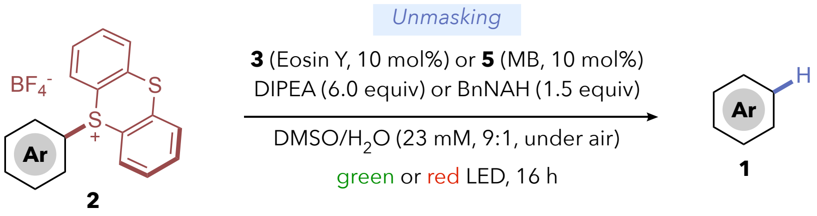

Under air atmosphere, a 6 mL screw-cap glass vial equipped with a Teflon-coated magnetic stirring bar was charged with an aryl thianthrenium salt **2** (1.0 equiv, 0.050 mmol), the corresponding reductant (either DIPEA, 6.0 equiv or BnNAH, 1.5 equiv) and photocatalyst (either Eosin Y disodium salt, 5 mol% or methylene blue, 10 mol%). Everything was dissolved in a 9:1 mixture of DMSO/water (2.0 mL, 25 mM). Then, the vial was closed with the corresponding cap and loaded into the photoredox set up (Kessil lamp and Hepatochem PhotoRedOx Duo^{TM}, see Section 2 for details). Then, the vial was irradiated at the corresponding wavelength (either green centered at 525 nm, or red centered at 660 nm) upon stirring over 16 h. After this time, irradiation was switched off and 1.0 equiv of 1,3,5-trimethoxybenzene (internal standard) was added as a solution in 2.0 mL of EtOAc, followed by the addition of ca. 3 mL of water. After shaking, the organic phase was extracted with a glass Pasteur pipette, dried with anhydrous Na_2SO_4, filtered, evaporated in high vacuum. Yields of unmasked products **1** and of released thianthrene (TT) were determined by crude ^{1}H NMR analysis.

All of the released products **1** are commercially available and unequivocal identification was performed by comparison with pure commercial samples by ^{1}H NMR and TLC. The crude ^{1}H NMR spectra in $CDCl_3$ that were used for identification and quantification of the product of unmasking are included below, together with specific details for each particular in vitro experiment. ^{1}H NMR of the masked compound is also included in the comparisons for the cases where it was characterized in $CDCl_3$. However, in most cases, they were characterized in MeCN-d_3 due to solubility, and direct comparison is not possible.

Note: In certain cases for which ^{1}H NMR quantification of the uncaged product is not fully reliable due to volatility (e.g.: release of anisole) or signal overlap (e.g.: in optimization/control experiments in Section 4, for reactions that did not reach full conversion and a non-overlapping peak between the caged and uncaged product could be used), the yield of the uncaging reaction was determined by integration of the ^{1}H NMR signal of the released free thianthrene (TT), since no side-products coming from the aryl-radical fragment were observed in any case. This is noted throughout this section whenever the situation arises.

5.2. Experimental details and yield determination for the unmasking step

Pyriproxyfen (1a)

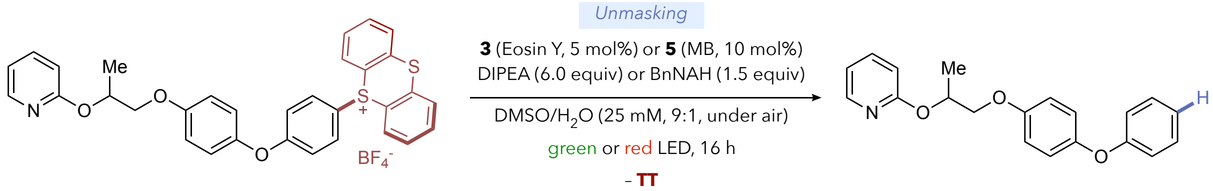

The in vitro release of the title product was carried out following General procedure B, starting from pyriproxyfen thianthrenium salt **2a** (31 mg, 0.050 mmol, 1.0 equiv) in DMSO/water (9:1, 2.0 mL, 25 mM) using the combinations of photocatalyst and reductant listed below. The yield of uncaged product was determined by ^{1}H NMR after extraction using 1.0 equiv of 1,3,5-trimethoxybenzene as internal standard.

- Eosin Y **3** (5 mol%), green LED light and DIPEA (6.0 equiv): >95% yield of **1a** (+95% **TT**).
- Eosin Y **3** (5 mol%), green LED light and BnNAH (1.5 equiv): 95% yield of **1a** (+95% **TT**).
- Methylene blue **5** (10 mol%), red LED light and DIPEA (6.0 equiv): 95% yield of **1a** (+95% **TT**).
- Methylene blue **5** (10 mol%), red LED light and BnNAH (1.5 equiv): 70% yield of **1a** (+75% **TT**).

^{1}H crude NMR for yield determination (A) and comparison with an original sample of free arene and of masked pyriproxyfen thianthrenium salt (**2a**). Quantitative release of thianthrene (TT) can also be observed.

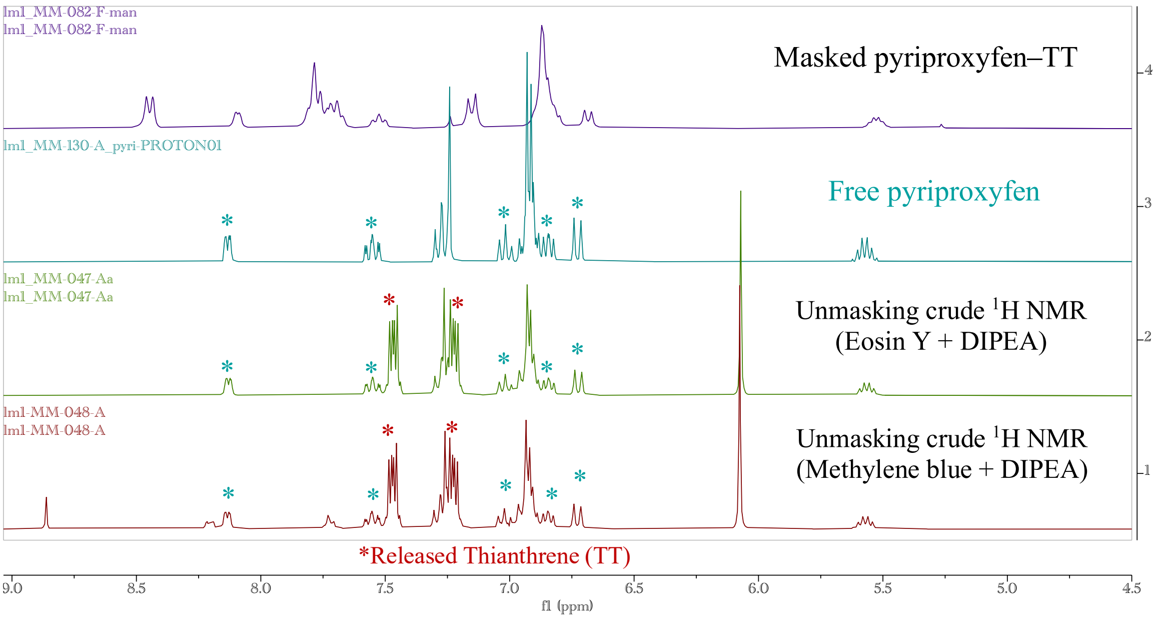

Napropamide (1b)

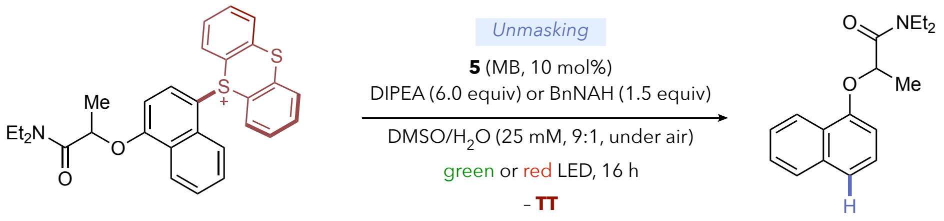

The in vitro release of the title product was carried out following General procedure B, starting from napropamide thianthrenium salt **2b** (14 mg, 0.025 mmol, 1.0 equiv) in DMSO/water (9:1, 1.0 mL, 25 mM) using the combinations of photocatalyst and reductant listed below. The yield of uncaged product was determined by ^{1}H NMR after extraction using 1.0 equiv of 1,3,5-trimethoxybenzene as internal standard.

- Methylene blue **5** (10 mol%), red LED light and DIPEA (6.0 equiv): >95% yield of **1b** (+95% **TT**).
- Methylene blue **5** (10 mol%), red LED light and BnNAH (1.5 equiv): >95% yield of **1b** (+95% **TT**).

^{1}H crude NMR for yield determination and comparison with an original sample of free arene:

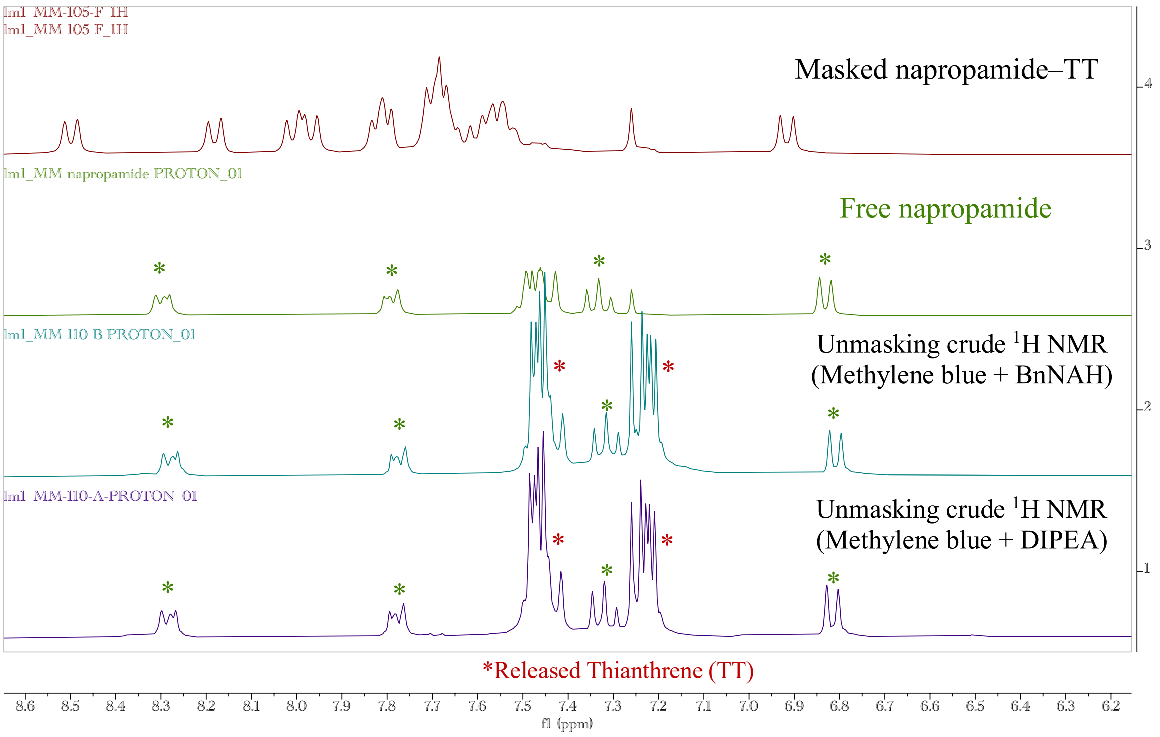

Boscalid (1c)

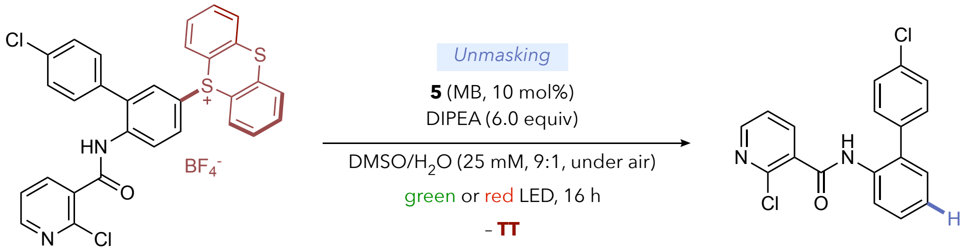

The in vitro release of the title product was carried out following General procedure B, starting from boscalid thianthrenium salt **2c** (32 mg, 0.050 mmol, 1.0 equiv) in DMSO/water (9:1, 2.0 mL, 25 mM) using the combinations of photocatalyst and reductant listed below. The yield of uncaged product was determined by ^{1}H NMR after extraction using 1.0 equiv of 1,3,5-trimethoxybenzene as internal standard.

- Methylene blue **5** (10 mol%), red LED light and DIPEA (6.0 equiv): >95% yield of **1c** (+95% **TT**).

^{1}H crude NMR for yield determination and comparison with an original sample of free arene:

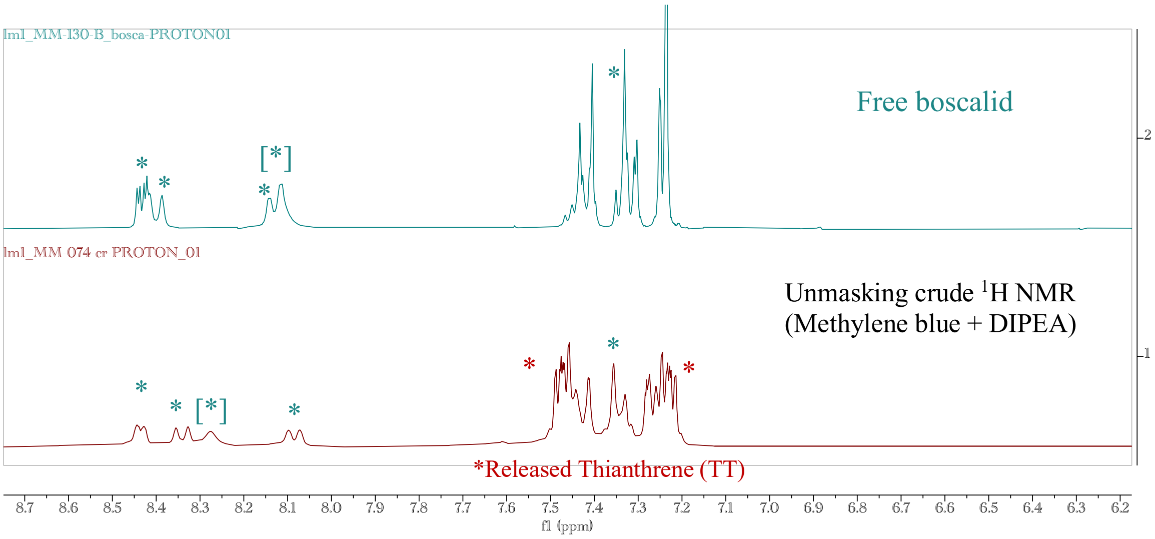

* Some ^{1}H signals (especially the NH [*]) are shifted/broadened due to the presence of significant amounts of DMSO and water in the crude mixture.

GW-9662 (1d)

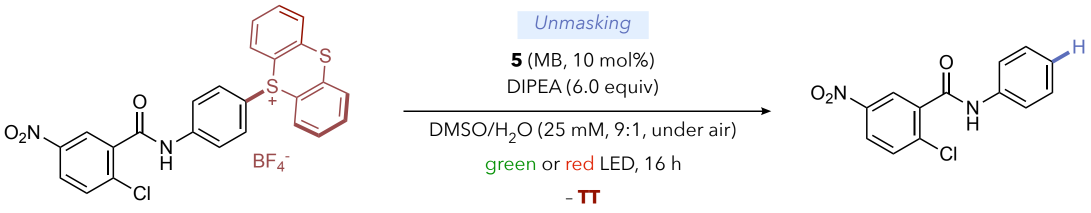

The in vitro release of the title product was carried out following General procedure B, starting from GW-9662 thianthrenium salt **2d** (15 mg, 0.025 mmol, 1.0 equiv) in DMSO/water (9:1, 1.0 mL, 25 mM) using the combinations of photocatalyst and reductant listed below. The yield of uncaged product was determined by ^{1}H NMR after extraction using 1.0 equiv of 1,3,5-trimethoxybenzene as internal standard.

- Methylene blue **5** (10 mol%), red LED light and DIPEA (6.0 equiv): 88% yield of **1d** (+95% **TT**).

^{1}H crude NMR for yield determination and comparison with an original sample of free arene:

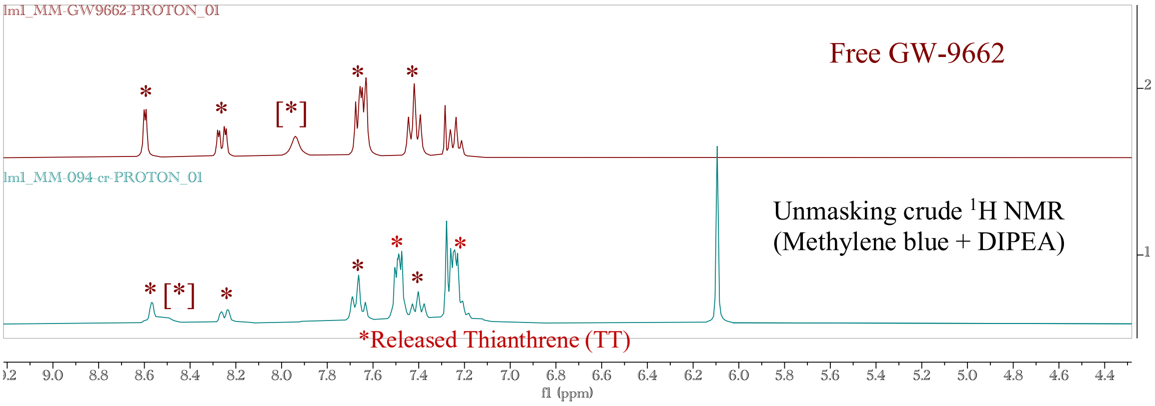

* Some ^{1}H signals (especially the NH [*]) are shifted/broadened due to the presence of significant amounts of DMSO and water in the crude mixture.

Anisole (1e)

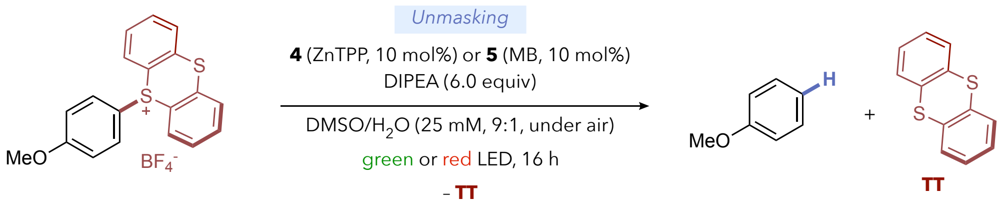

The in vitro release of the title product was carried out following General procedure B, starting from anisole thianthrenium salt **2e** (7.3 mg, 0.016 mmol, 1.0 equiv) in DMSO/water (9:1, 1.0 mL, 16 mM) using the combinations of photocatalyst and reductant listed below. The yield of uncaged product was determined by ^{1}H NMR after extraction using 1.0 equiv of 1,3,5-trimethoxybenzene as internal standard.

- ZnTPP **4** (10 mol%), red LED light and DIPEA (6.0 equiv): >95% yield based on **TT**.
- Methylene blue **5** (10 mol%), red LED light and DIPEA (6.0 equiv): >95% yield based on **TT**.

^{1}H crude NMR for yield determination. Due to volatility of the released anisole, yield was determined according to the amount of released thianthrene (**TT**):

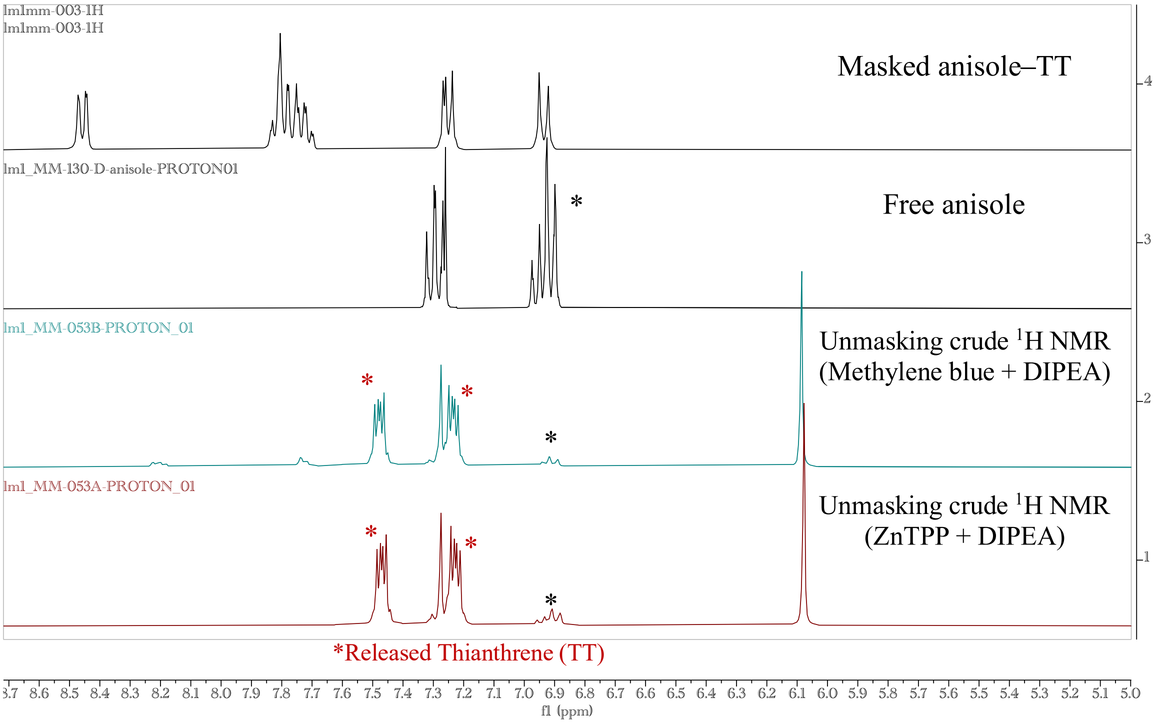

1-Bromo-4-phenoxybenzene (1f)

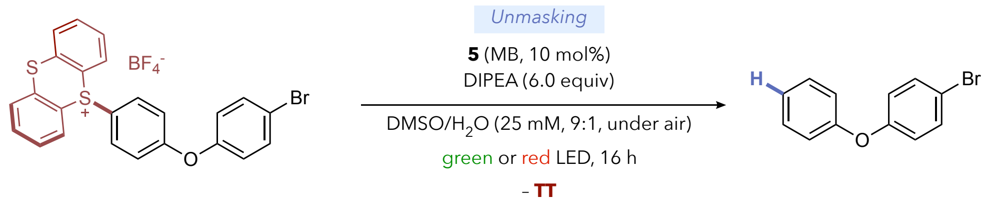

The in vitro release of the title product was carried out following General procedure B, starting from 1-bromo-4-phenoxybenzene thianthrenium salt **2f** (14 mg, 0.025 mmol, 1.0 equiv) in DMSO/water (9:1, 1.0 mL, 25 mM) using the combinations of photocatalyst and reductant listed below. The yield of uncaged product was determined by ^{1}H NMR after extraction using 1.0 equiv of 1,3,5-trimethoxybenzene as internal standard.

- Methylene blue **5** (10 mol%), red LED light and DIPEA (6.0 equiv): 80% yield of **1f** (+95% **TT**).

^{1}H crude NMR for yield determination and comparison with an original sample of free arene:

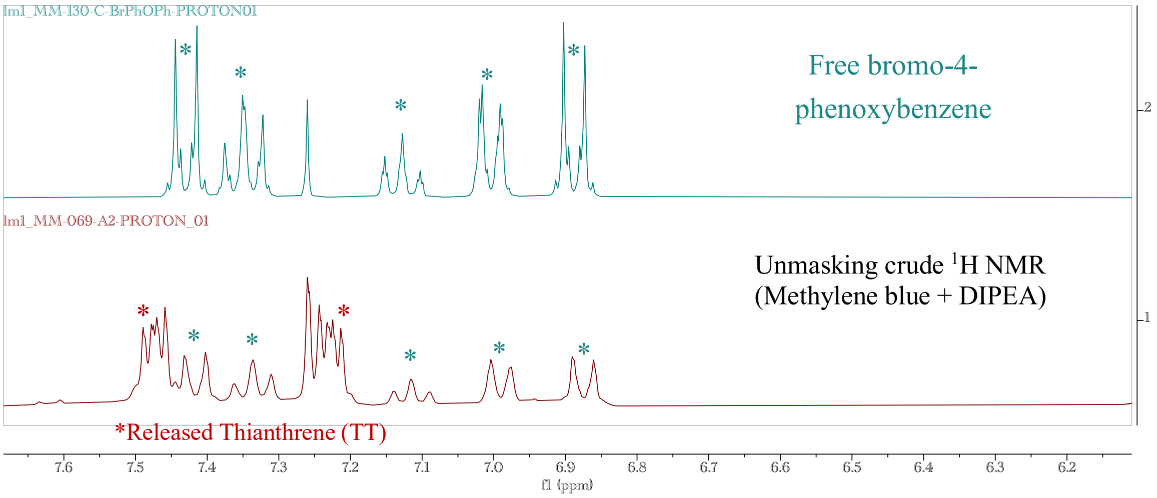

1,1,2,2-Tetraphenylethylene (TPE, **1g**)

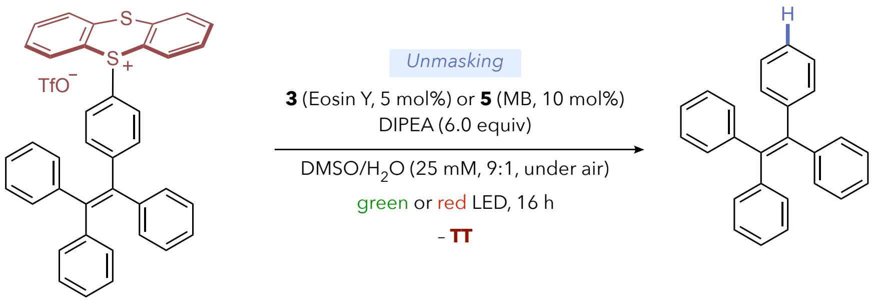

The in vitro release of the title product was carried out following General procedure B, starting from TPE thianthrenium salt **2g** (17 mg, 0.025 mmol, 1.0 equiv) in DMSO/water (9:1, 1.0 mL, 25 mM) using the combinations of photocatalyst and reductant listed below. The yield of uncaged product was determined by ^{1}H NMR after extraction using 1.0 equiv of 1,3,5-trimethoxybenzene as internal standard.

- Eosin Y **3** (5 mol%), green LED light and DIPEA (6.0 equiv): >95% yield of **1g** (+95% **TT**).
- Methylene blue **5** (10 mol%), red LED light and DIPEA (6.0 equiv): >95% yield of **1g** (+95% **TT**).

^{1}H crude NMR for yield determination and comparison with an original sample of free arene:

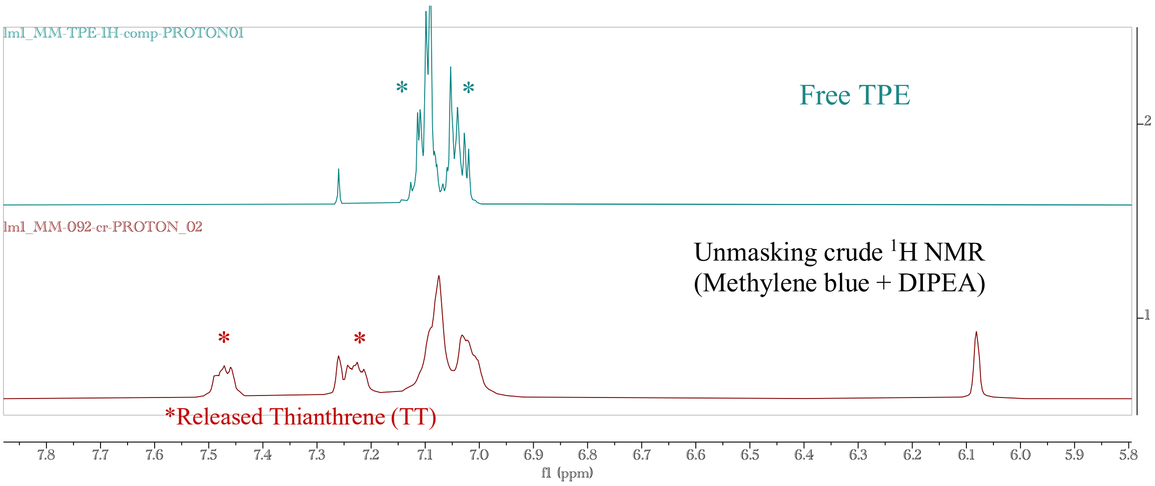

Lidocaine (SI-1)

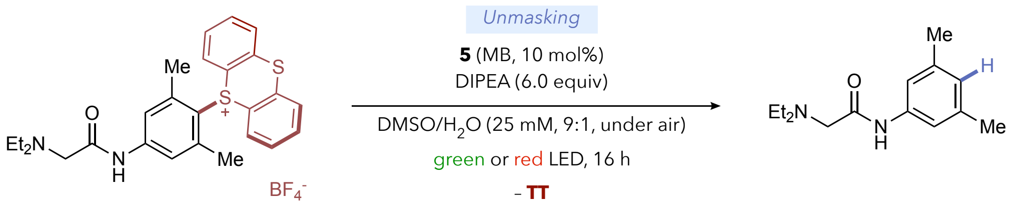

The in vitro release of the title product was carried out following General procedure B, starting from lidocaine thianthrenium salt **SI-1** (13 mg, 0.025 mmol, 1.0 equiv) in DMSO/water (9:1, 1.0 mL, 25 mM) using the combinations of photocatalyst and reductant listed below. The yield of uncaged product was determined by ^{1}H NMR after extraction using 1.0 equiv of 1,3,5-trimethoxybenzene as internal standard.

- Methylene blue **5** (10 mol%), red LED light and DIPEA (6.0 equiv): 79% of lidocaine (+95% **TT**).

^{1}H crude NMR for yield determination and comparison with an original sample of free arene:

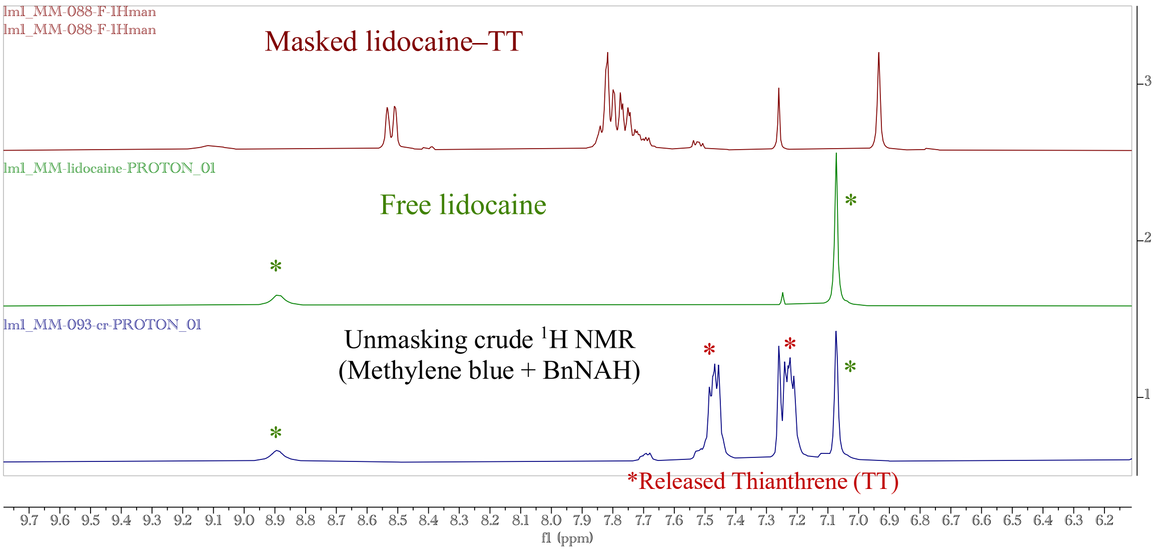

Unmasking of anisole from its dibenzothiophene-derived salt (SI-2)

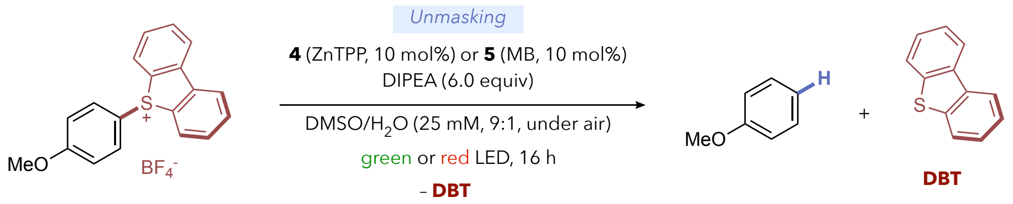

The in vitro release of the title product was carried out following General procedure B, starting from anisole DBT thianthrenium salt **SI-2** (11 mg, 0.025 mmol, 1.0 equiv) in DMSO/water (9:1, 1.0 mL, 25 mM) using the combinations of photocatalyst and reductant listed below. The yield of uncaged product was determined by ^{1}H NMR after extraction using 1.0 equiv of 1,3,5-trimethoxybenzene as internal standard.

- Eosin Y **3** (5 mol%), green LED light and DIPEA (6.0 equiv): >95% yield based on **DBT**.
- Eosin Y **3** (5 mol%), green LED light and BnNAH (1.5 equiv): >95% yield based on **DBT**.
- Methylene blue **5** (10 mol%), red LED light and DIPEA (6.0 equiv): 95% yield based on **DBT**.
- Methylene blue **5** (10 mol%), red LED light and BnNAH (1.5 equiv): 85% yield based on **DBT**.

^{1}H crude NMR for yield determination, due to volatility of the released anisole, yield was determined according to the amount of released dibenzothiophene (**DBT**):

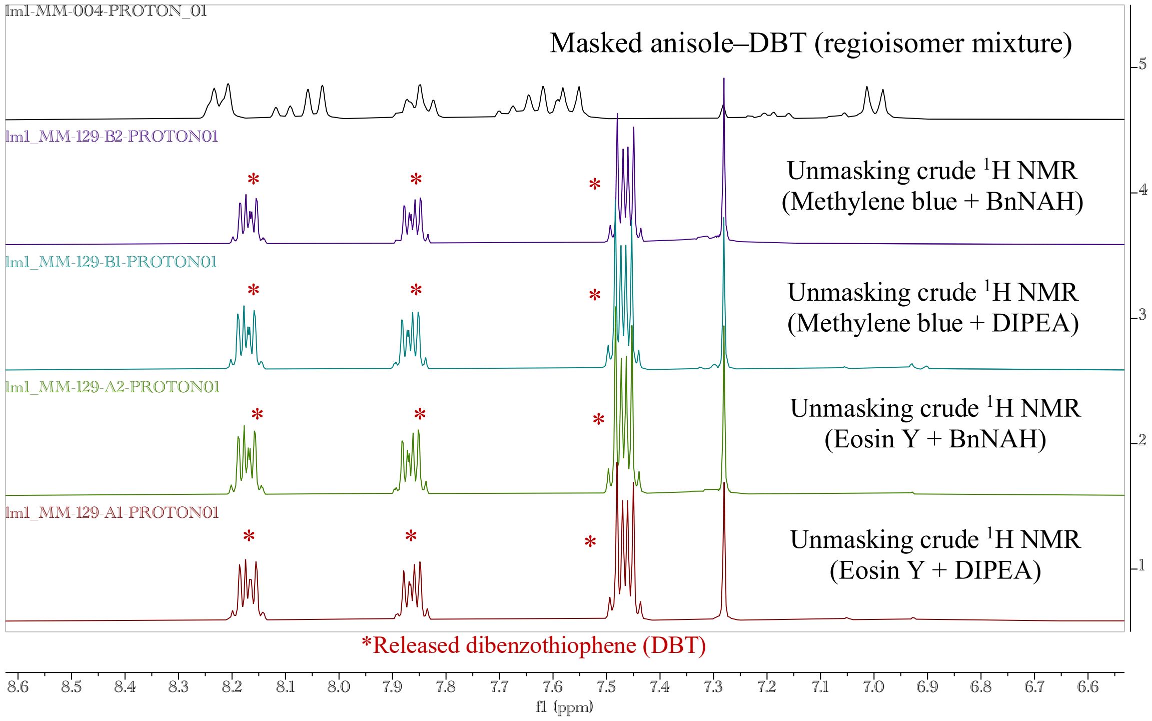

6. Photophysical properties

6.1. UV–Vis absorption spectroscopy

General information

UV–Vis absorption spectroscopy was performed using a Jasco V-770 UV–Vis spectrophotometer at room temperature (ca. 25 ºC). Sample solutions of the appropriate concentration were freshly prepared in HPLC-grade DMSO. Absorption measurements were performed under air either in 10×2 mm disposable plastic cuvettes (l = 10 mm) or in 10×10 mm quartz cuvettes (l = 10 mm).

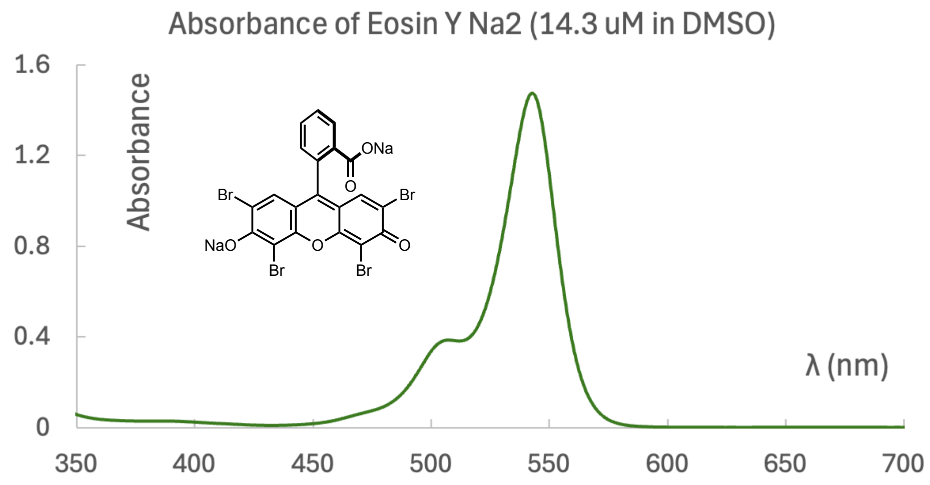

Figure S7. UV–Vis absorption spectrum of Eosin Y disodium salt **3** in DMSO (14.3 μM).

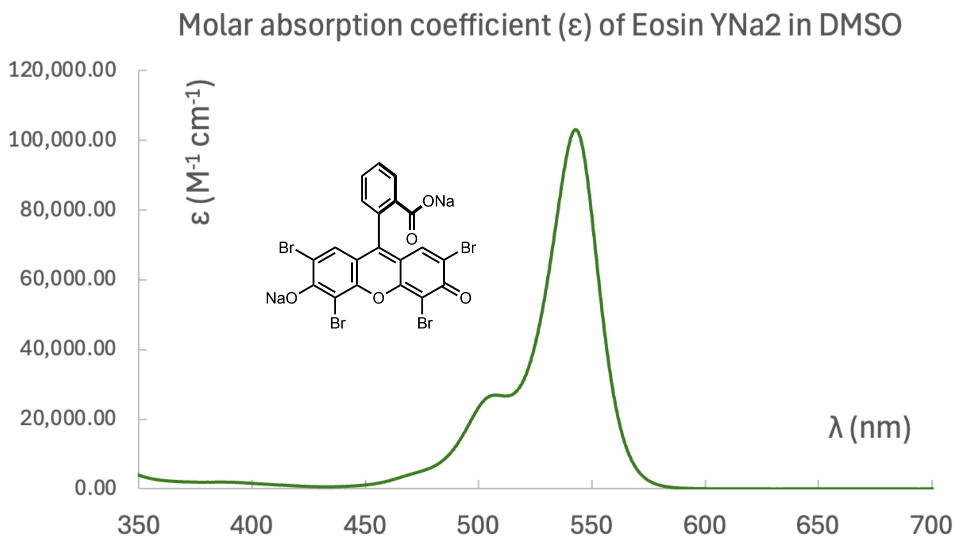

Figure S8. Molar absorption coefficient of Eosin Y disodium salt **3** in DMSO (recorded at 14.3 μM, l = 10 mm).

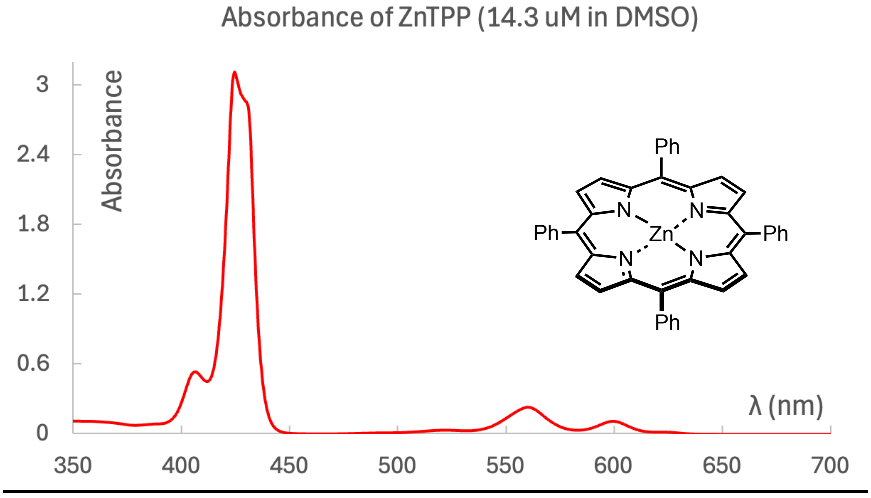

Figure S9. UV–Vis absorption spectrum of ZnTPP **4** in DMSO (14.3 μM).

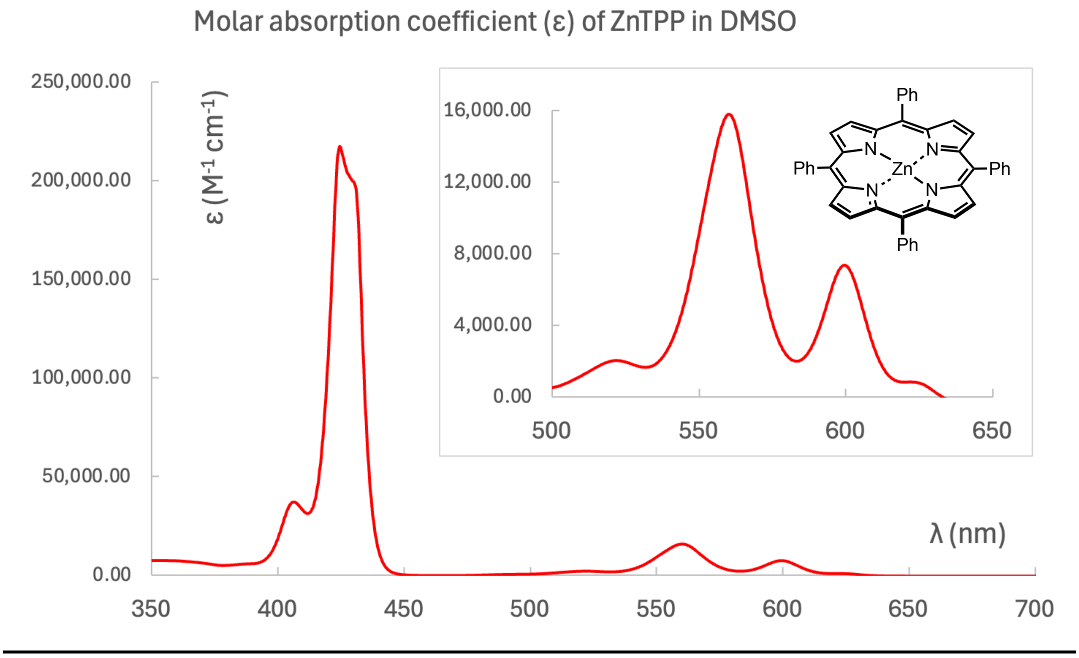

Figure S10. Molar absorption coefficient of ZnTPP **4** in DMSO (recorded at 14.3 μM, l = 10 mm).

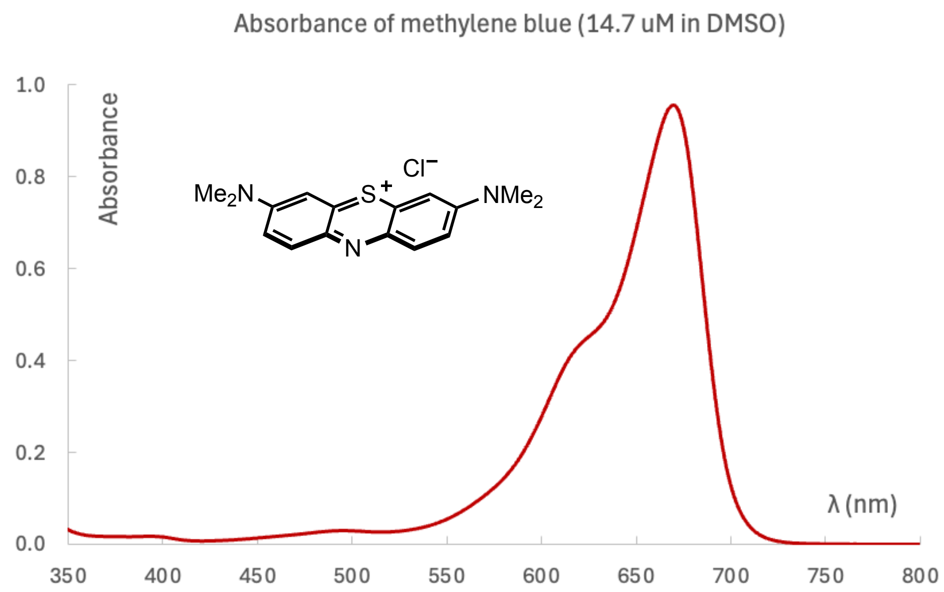

Figure S11. UV–Vis absorption spectrum of methylene blue **5** in DMSO (14.7 μM).

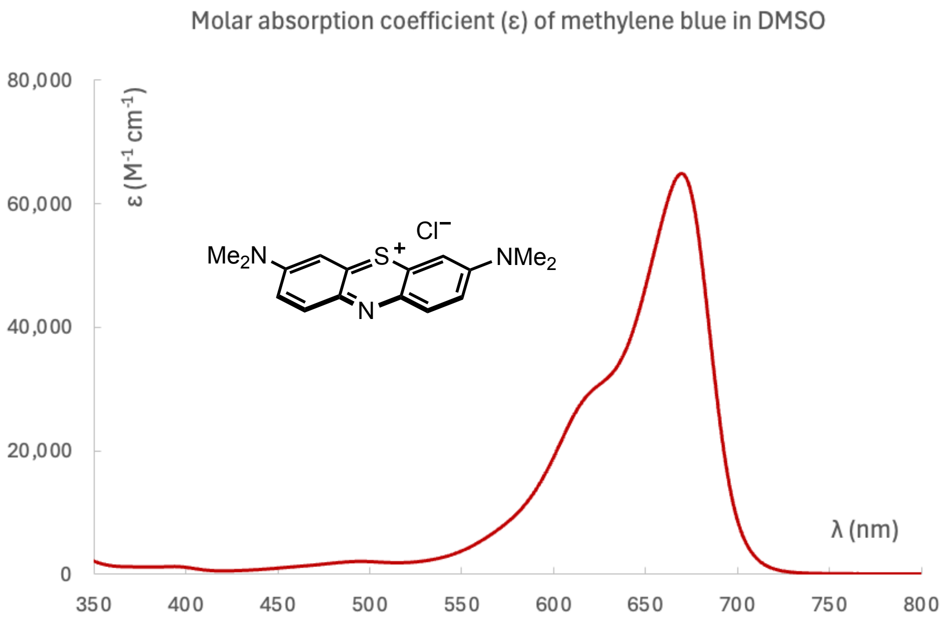

Figure S12. Molar absorption coefficient of methylene blue **5** in DMSO (recorded at 14.7 μM, *l* = 10 mm).

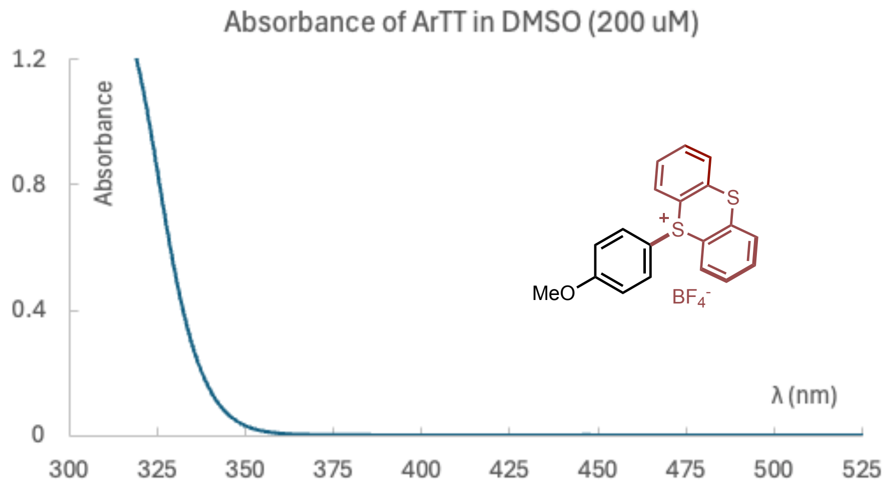

Figure S13. UV–Vis absorption spectrum of 4-anisole thianthrenium salt **2e** in DMSO (150 μM).

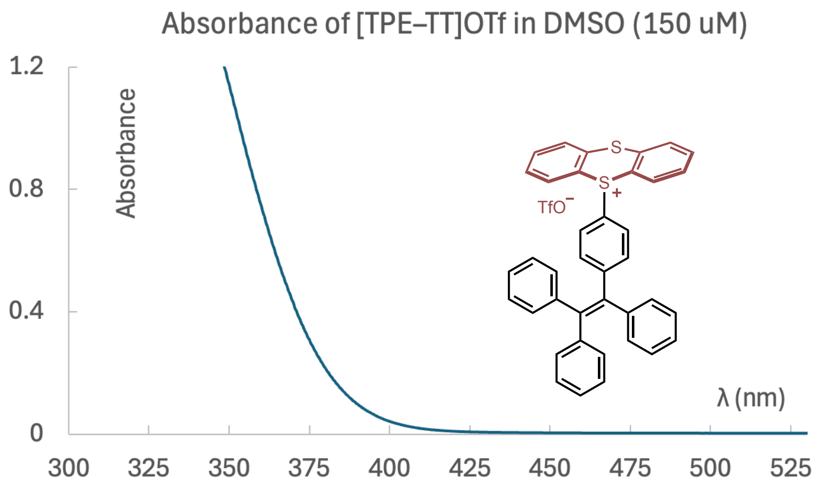

Figure S14. UV–Vis absorption spectrum of TPE aryl thianthrenium salt **2g** in DMSO (150 μM).

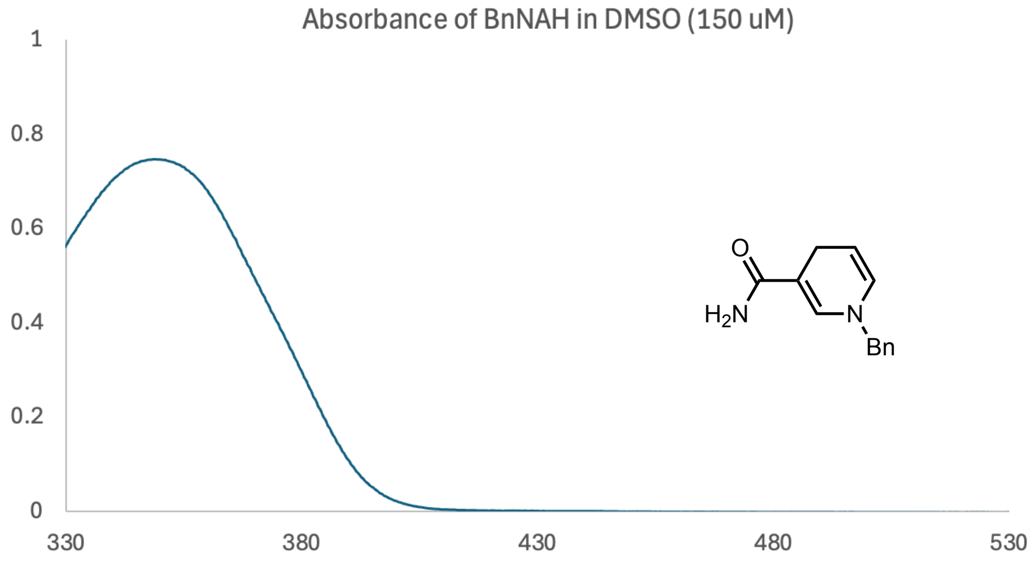

Figure S15. UV–Vis absorption spectrum of BnNAH **6** in DMSO (150 μM).

* The absorbance of these compounds was recorded in plastic cuvettes, which do not transmit light below 300 nm.

6.2. Emission spectroscopy (fluorescence)

General information

Fluorescence spectroscopy was performed using a FS5 spectrofluorometer at room temperature (ca. 25 ºC). Sample solutions of the appropriate concentration were freshly prepared in HPLC-grade DMSO. Emission measurements were performed under air in 10×10 mm quartz cuvettes (l = 10 mm). For specific parameters for each compound, see Section 8.2.

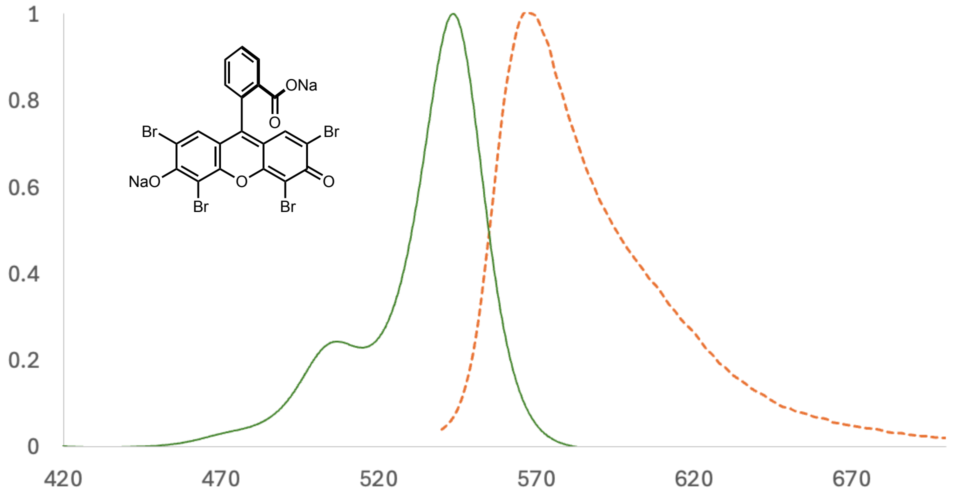

Figure S16. Normalized emission (orange dashed trace; excitation wavelength = 530 nm) and UV–Vis absorption (green trace) spectra of Eosin Y disodium salt **3** in DMSO.

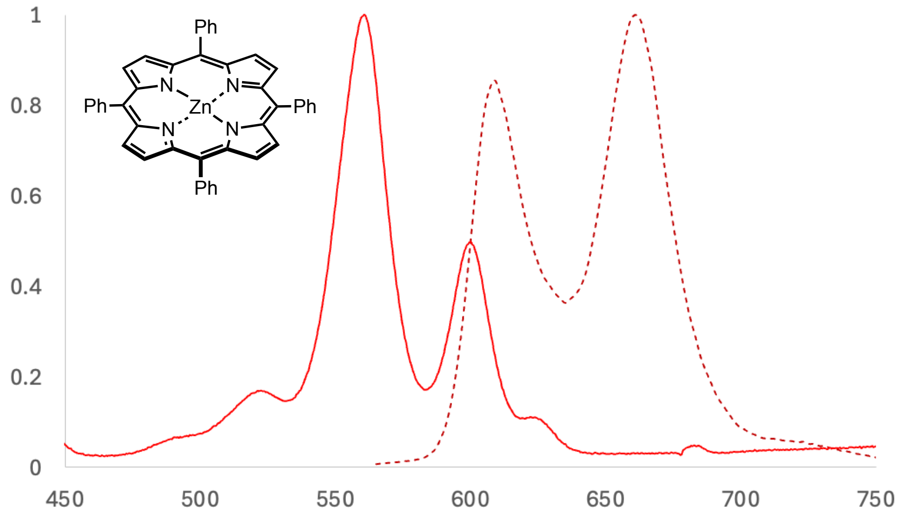

Figure S17. Normalized emission (excitation wavelength = 560 nm) and UV–Vis absorption spectra of ZnTPP **4** in DMSO.

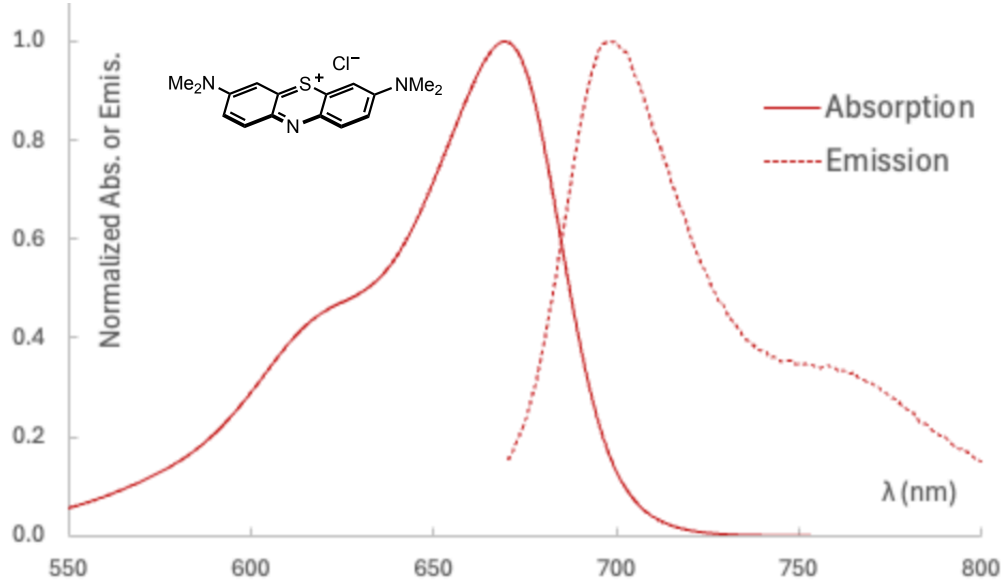

Figure S18. Normalized emission (excitation wavelength = 668 nm) and UV–Vis absorption spectra of methylene blue **5** in DMSO.

7. Mechanistic and photophysical experiments

7.1. Overlap of absorption spectra and emission of the light sources

Absorption of the reaction components vs emission of the LEDs

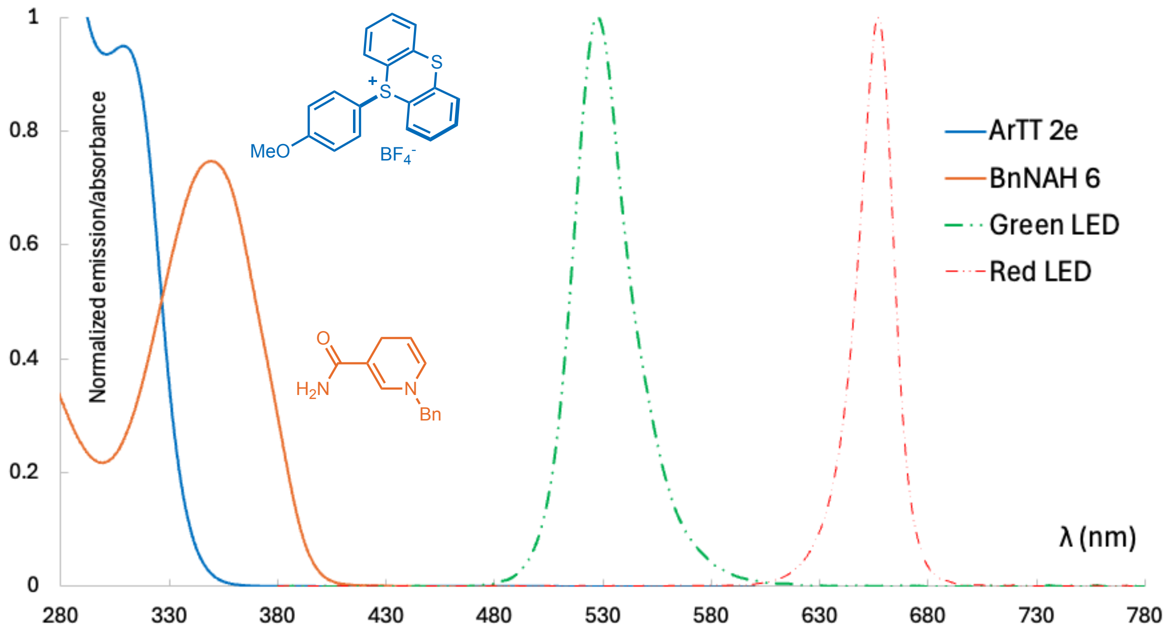

Figure S19. Normalized UV–Vis absorption spectra of ArTT **2e** and BnNAH **6** in DMSO and emission spectra of the green and red LED used in this study.

Neither the reaction substrate nor the reactant absorb at the wavelengths of emission of the two LEDs used in this study, justifying the requirement of photocatalysts.

The lack of reactivity in the absence of photocatalysts suggests that the hypothetical formation of electron donor–acceptor complexes between the reactants does not lead to significant productive electron transfer events.

Absorption of the photocatalysts vs emission of the corresponding light source

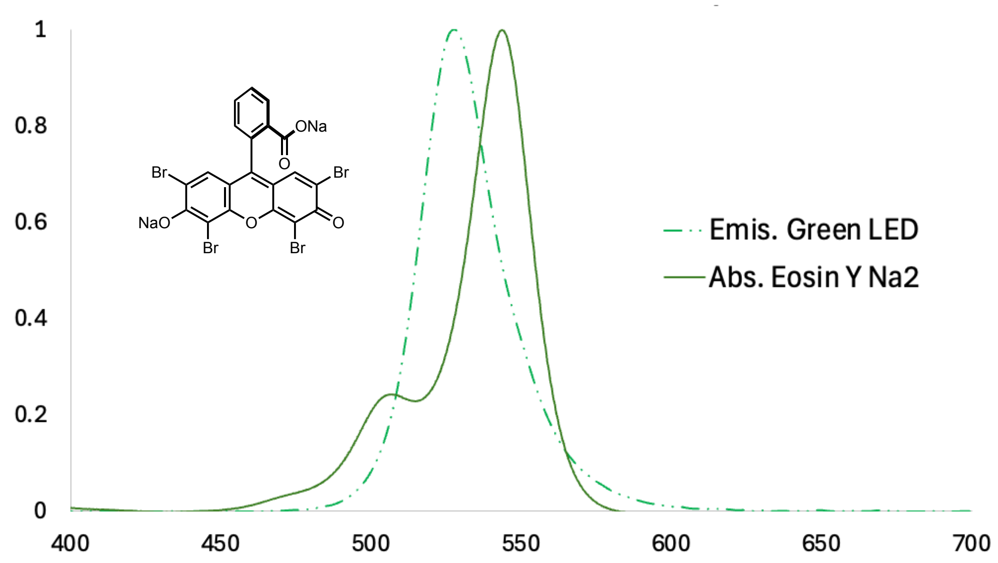

Figure S20. Normalized UV–Vis absorption spectrum of Eosin Y disodium salt **3** in DMSO and emission spectra of the green LED used in this study.

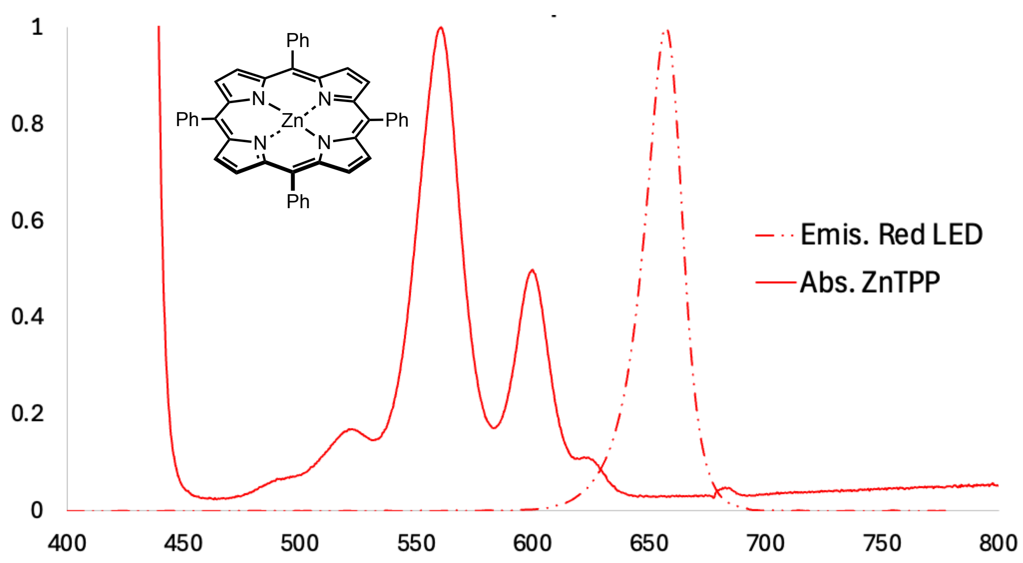

Figure S21. UV–Vis absorption spectrum of ZnTPP **4** in DMSO and emission spectra of the red LED used in this study.

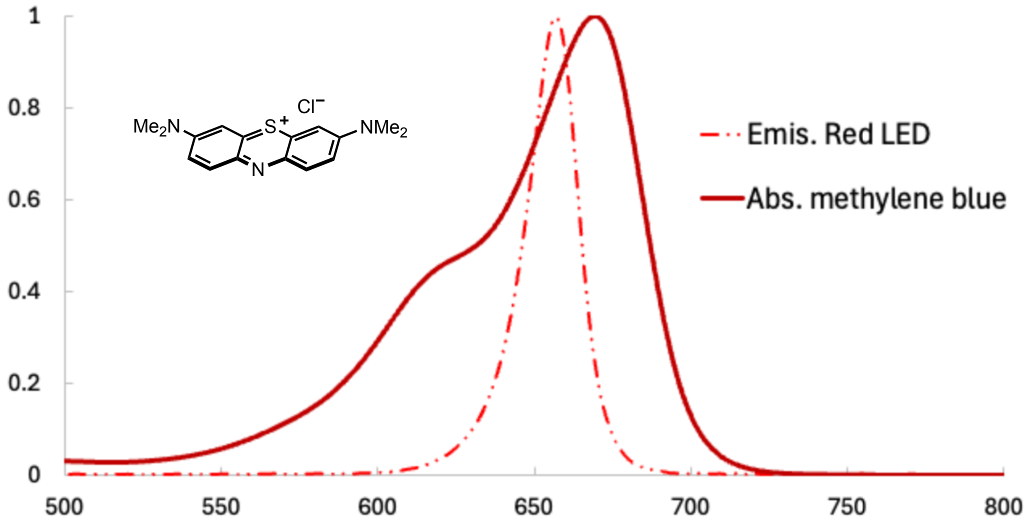

Figure S22. UV–Vis absorption spectrum of methylene blue **5** in DMSO and emission spectra of the red LED used in this study.

7.2. Absorption and AIE emission of protected and unprotected TPE

Absorption of the reaction components present in the fluorescence-microscope experiments

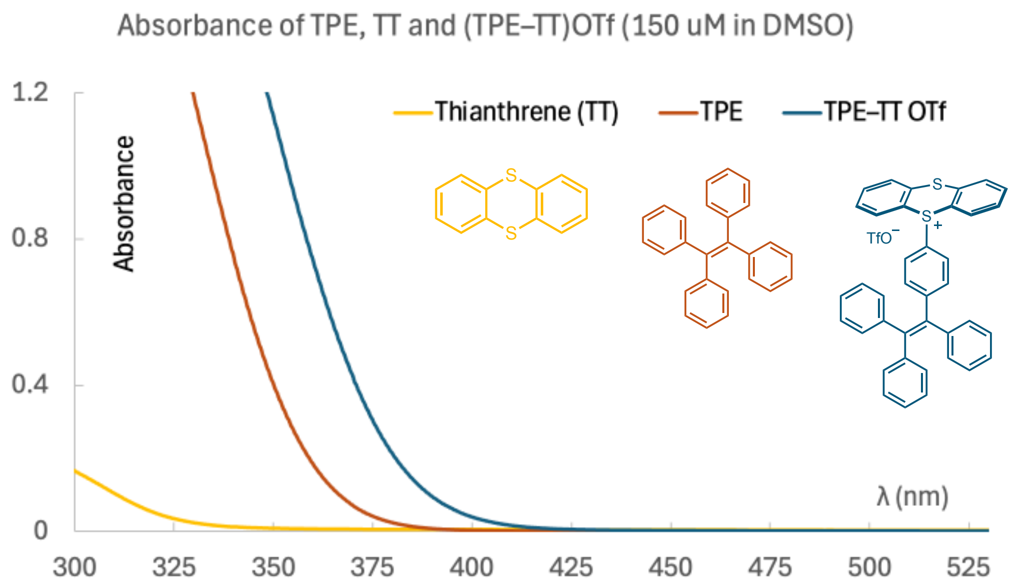

Figure S23. Overlapped UV–Vis absorption spectra of thianthrene (TT), 1,1,2,2-tetraphenylethylene (TPE, **1g**) and TPE–TT **2g** in DMSO.

As described in Section 11, fluorescence microscopy was conducted using a 365 nm excitation light source (https://www.coolled.com/products/pe-300white/), which tails between 360 and 400 nm, clearly absorbed by **2g** (TPE–TT).

Study on AIE properties of protected [TPE–TT]OTf (2g)

1,1,2,2-Tetraphenylethylene (TPE) **1g** and TPE–TT **2g**, were evaluated for their aggregation-induced fluorescence emission (AIE) effect. TPE is highly insoluble in aqueous media, whereas TPE–TT is expected to be more soluble due to the presence of the thianthrene protecting group. Stock solutions of both compounds were prepared in dimethyl sulfoxide (DMSO) and subsequently diluted to a final concentration of 50 μM in two different media: DMSO or phosphate-buffered saline (PBS). The use of PBS aimed to simulate AIE effects in an aqueous biological environment, assessing whether it would be feasible to accumulate TPE–TT within cellular systems and observe fluorescence emission.

TPE and TPE–TT 50 μM dilutions in the above-mentioned media were placed into a 24-well plate and fluorescence emission spectra were recorded using a microplate reader (Tecan Infinite 200Pro) controlled by Tecan i-control software (version 2.0.10.0). Spectra were recorded in the range of 380–700 nm upon excitation at 350 nm.

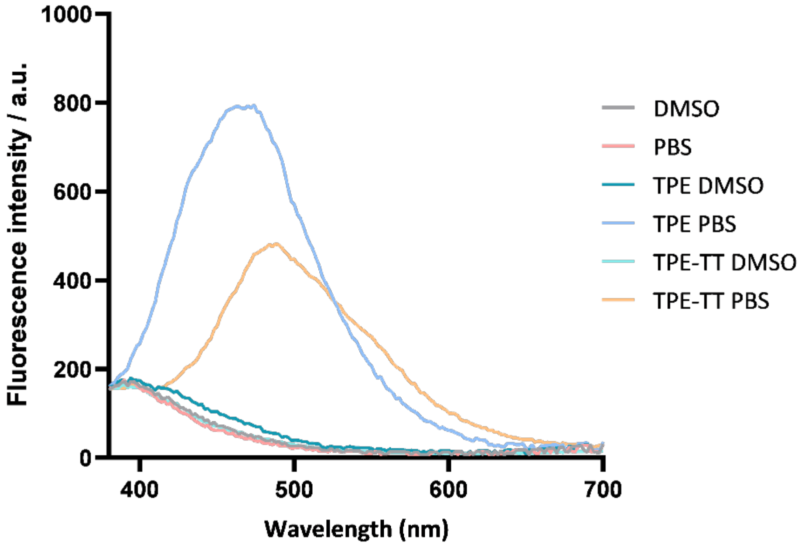

Figure S24. Steady-state fluorescence emission spectra of TPE and TPE–TT in DMSO and PBS. λ_{ex} = 350 nm.

As expected, steady-state fluorescence measurements in DMSO show very weak emissive properties of these compounds in solution, whereas both of them are significantly emissive in an aqueous PBS medium. While TPE–TT shows partially attenuated AIE fluorescence compared to free TPE (which is expected as it is more water soluble), both of them are sufficiently emissive to be observed by fluorescence microscopy and imaging of cells and bacteria (see Section 11).

7.3. Fluorescence quenching studies

Fluorescence quenching studies were performed using a FS5 spectrofluorometer at room temperature (ca. 25 ºC), according to typical procedures.[7] Sample solutions of the appropriate concentration were freshly prepared in HPLC-grade DMSO. Emission measurements were performed under air in 10×10 mm quartz cuvettes (*l* = 10 mm).

In order to minimize inner filter effects, the following excitation wavelength with absorption value between 0.1–0.2 at the photocatalyst concentration was selected:

- Eosin Y disodium salt **3** in DMSO: Excitation at 490 nm (Absorbance = 0.19 at 14.3 μM)

The following additional experimental settings were employed:

- 495–750 nm scan range (step = 1 nm). Scan slit: 1.2 nm. Fixed/offset slit: 1.0 nm.

Fluorescence quenching experiments were performed under air, simulating the reaction experimental conditions. The following stock solutions were prepared in DMSO:

- A) 10 mL of a 0.0005 M solution of Eosin Y disodium salt **3**.
- B) 20 mL of a 0.02 M solution of aryl thianthrenium salt **2e**.
- C) 20 mL of a 0.02 M solution of BnNAH **6**.

From this stock solutions, the following set of final solutions were prepared for each combination of photocatalyst and quencher (3.5 mL total volume for the cuvette).

- I) 14.3 μM in photocatalyst + 498 μM of quencher.
- II) 14.3 μM in photocatalyst + 956 μM of quencher.
- III) 14.3 μM in photocatalyst + 1811 μM of quencher.
- IV) 14.3 μM in photocatalyst + 3339 μM of quencher.
- V) 14.3 μM in photocatalyst + 6670 μM of quencher.

The 3.5 mL 10×10 mm fluorescence quartz cuvettes were filled with the corresponding solution, starting from I and up to V. Between cuvettes of the same set, the cuvette was rinsed twice with DMSO before the addition of the next solution. After a set of fluorescence measurements, the cuvette was fully washed with water, acetone and Hellmanex™, followed by another water rinse and then a final acetone rinse. Fluorescence emission was recorded for each solution and the results for each combination of photocatalyst/quencher is reported in the following pages. For Stern–Volmer analysis, the fluorescence intensity value at the emission maximum was used.

Quenching of Eosin Y

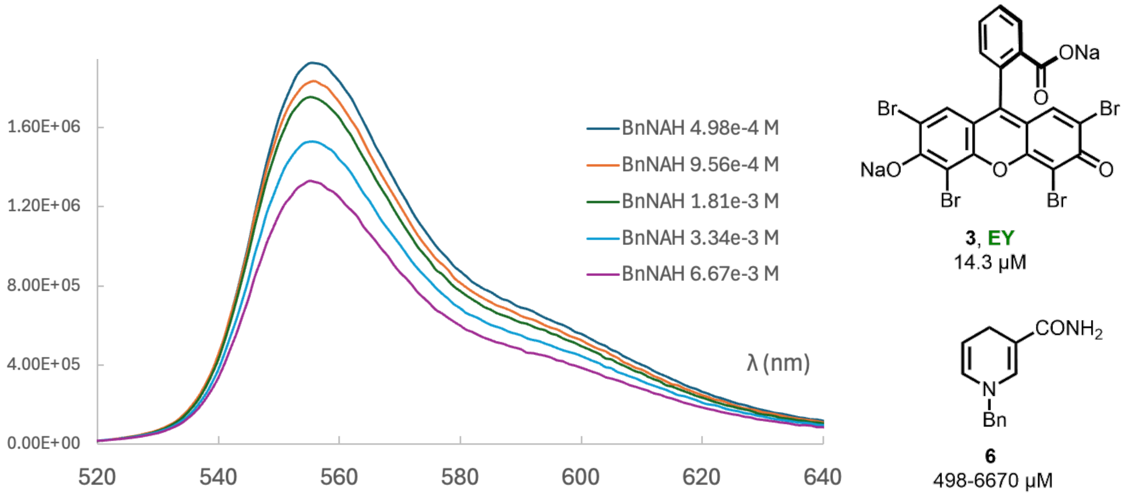

Figure S25. Overlapped steady-state fluorescence emission spectra of Eosin Y disodium salt **3** (14.3 μM) in the presence of different concentrations of quencher **6** (498–6670 μM). λ_{ex} = 490 nm.

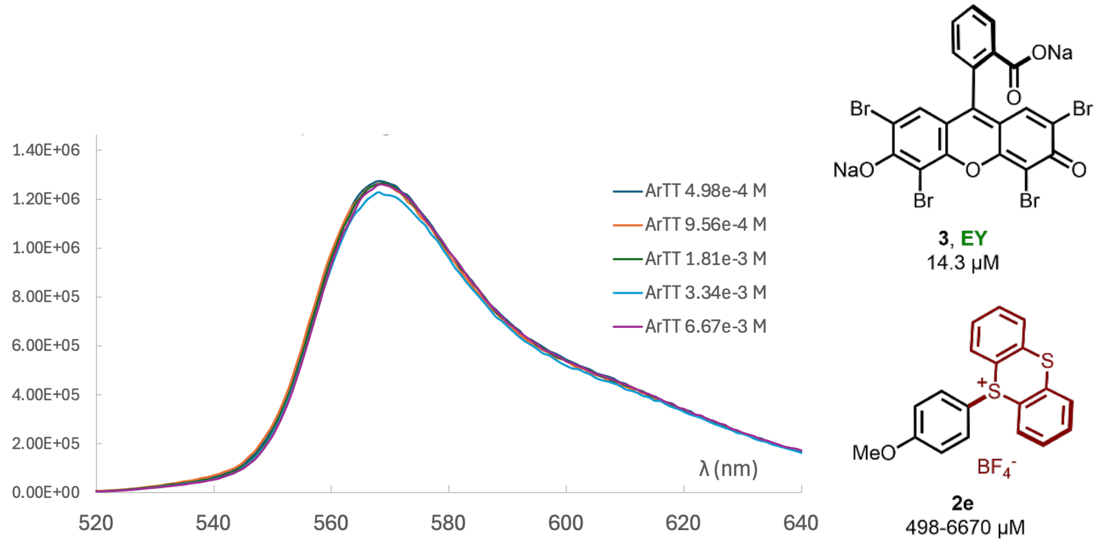

Figure S26. Overlapped steady-state fluorescence emission spectra of Eosin Y disodium salt **3** (14.3 μM) in the presence of different concentrations of aryl thianthrenium salt **2e** (498–6670 μM). λ_{ex} = 490 nm.

A significant quenching effect was observed for BnNAH **6** with Eosin Y (**3**), while no quenching effect was detected with ArTT **2e**. This suggests that a reductive-quenching photoredox mechanism is at least operative to some extent, while a hypothetical oxidative-quenching could not be identified.

It is worth noting that steady-state fluorescence quenching analysis only delivers direct information about the reactivity of singlet excited states (which for Eosin Y are long-lived enough [ca. 5 ns] to engage in bimolecular interactions). Triplet-state reactivity could also be operative.

Stern–Volmer analysis

We constructed Stern–Volmer plots based on the fluorescence intensity at the emission maximum of Eosin Y in DMSO upon addition of increasing concentrations of quenchers **2e** and BnNAH **6**. The fluorescence intensity value at the emission maximum was used. This ratio was plotted as a function of the quencher concentration [Q] as shown in Figure S27.

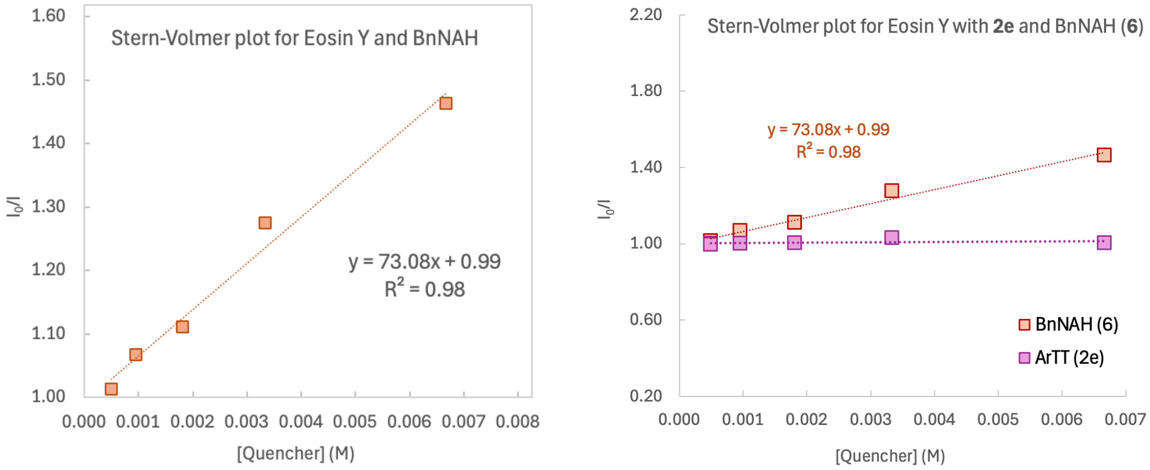

Figure S27. Stern–Volmer plot for the fluorescence quenching of Eosin Y disodium salt **3** with BnNAH **6** (left) and overlapped effect of BnNAH **6** vs **2e** (right).

The rate of the quenching process was calculated according to the Stern–Volmer equation:

$$\frac{I_0}{I} = 1 + K_{SV}[Q] = 1 + k_q\tau_0[Q]$$

where $\mathbf{K_{SV}}$ is the Stern–Volmer constant (slope), k_q is the bimolecular quenching rate and τ_0 is the excited state lifetime.

Linear fitting of the data points (Figure S27) with the Stern–Volmer equation provided a value of $\mathbf{K_{SV} = 73.1\ M^{-1}}$ for Eosin Y disodium salt **3** in DMSO with BnNAH **6**. Considering a reported value of singlet-state lifetime for Eosin Y of τ_0 = 4.5 ns,[8] a bimolecular quenching rate constant of $\boldsymbol{k_q} \mathbf{= 1.6 \times 10^{10}\ M^{-1}s^{-1}}$ was calculated. This value can be consistent with a diffusion-controlled bimolecular quenching (i.e.: all collisions between excited photocatalyst and quencher **6** results in reactivity). Alternatively, this could also be consistent with a static quenching process, which cannot be discarded before studying the system with fluorescence lifetime analysis.

Significant fluorescence quenching with **2e** was not observed at the explored concentrations.

8. Synthetic applications of the red-light-based photoredox strategy

8.1. Red-light photoredox C–C coupling of aryl radicals

2-(4-(4-Bromophenoxy)phenyl)-1-methyl-1*H*-pyrrole (8)

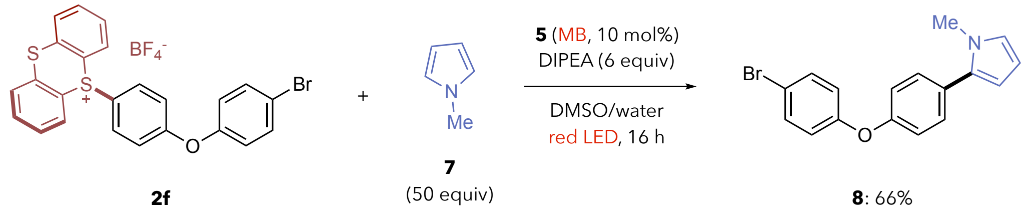

Under air atmosphere, a 6 mL screw-cap glass vial equipped with a Teflon-coated magnetic stir bar was charged with aryl thianthrenium salt **2f** (55 mg, 0.10 mmol, 1.0 equiv), *N*-methylpyrrole (410 mg, 0.45 mL, 5.0 mmol, 50 equiv) and methylene blue (**5**, 3.2 mg, 0.010 mmol, 10 mol%). Then, 9:1 DMSO/water (2.0 mL, 50 mM) was added as solvent, followed by DIPEA (78 mg, 105 µL, 0.60 mmol, 6.0 equiv). The vial was closed with the corresponding screw cap, loaded into the photoredox set up (Kessil lamp and Hepatochem PhotoRedOx Duo™, see Section 2 for details) and it was irradiated with 660 nm red light upon stirring over 16 h. After that time, the mixture was diluted with 10 mL of water and 10 mL of EtOAc. The organic fraction was washed once with water and once with brine, dried over anhydrous Na_2SO_4, filtered and concentrated in vacuum. The resulting residue was purified by preparative TLC in silica gel, using hexane/EtOAc 95:5 as eluent (one elution). This gave the title product as a white solid (19 mg, 58%).

Rf (95:5 hexane/EtOAc) = 0.6

^{1}H NMR (300 MHz, $CDCl_3$) δ 7.49 – 7.41 (m, 2H), 7.39 – 7.33 (m, 2H), 7.07 – 6.99 (m, 2H), 6.97 – 6.90 (m, 2H), 6.75 – 6.68 (m, 1H), 6.20 (dd, *J* = 2.3, 1.0 Hz, 2H), 3.71 – 3.63 (m, 3H).

^{13}C NMR (75 MHz, $CDCl_3$) δ 165.30, 156.38, 155.79, 132.74, 130.17, 129.73, 128.96, 123.51, 120.64, 118.73, 108.57, 107.76, 34.97.

HRMS (ESI+): calculated for $C_{17}H_{15}{}^{79}BrNO$ $[M+H]^+$: 328.0332; found: 328.0327.

We also performed a variety of control or optimization experiments with the reaction, following the procedure above but at 0.025 mmol scale, and determining the yields by ^{1}H NMR. The obtained results are summarized in the following table:

Table S7. Control and optimization experiments for the red-light-promoted photoredox C–C coupling of aryl thianthrenium salts. Unless stated otherwise, yields were determined by ^{1}H NMR using 1,3,5-trimethoxybenzene as internal standard. TT = released thianthrene. n/d = not detected. n/dm = not determined.

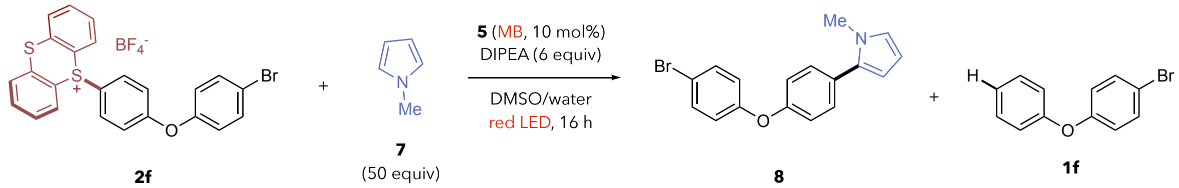

Entry	Deviations from above	Recov. **2f**	TT	**8**	**1f**
1	no deviations	n/d	85%	66% (57% isol)	n/dm
2	without light (660 nm)	>45%	n/d	n/d	n/dm
3	without photocatalyst **5** (MB)	>37%	<2%	<2%	n/dm
4	without DIPEA	>50%	<5%	<5%	n/dm
5	with K_2CO_3 instead of DIPEA	>10%	75%	62%	n/dm
6	without NMP (**7**)	n/d	95%	n/a	80%
7	without NMP (**7**) and without DIPEA	>41%	n/d	n/d	n/d

These experiments show the requirement of all components of the reaction. Removal of DIPEA led to no formation of product, even if a large excess of pyrrole **7** is present, which could potentially act as electron source for the activation of **2f**. Interestingly, we found that the addition of potassium carbonate instead of DIPEA restores the reactivity, suggesting that DIPEA could be playing a relevant role as a base for the deprotonation/rearomatization step after aryl-radical addition and subsequent oxidation.

8.2. Red-light nickel-metallaphotoredox C–N coupling

1-(4-(4-Bromophenoxy)phenyl)piperidine (10)

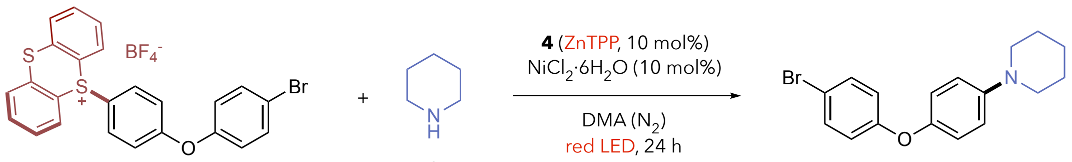

2f
9 (4.0 equiv)
10: 63%

A 6 mL screw-cap glass vial equipped with a Teflon-coated magnetic stir bar was charged with aryl thianthrenium salt **2f** (55 mg, 0.10 mmol, 1.0 equiv), $NiCl_2 \cdot 6H_2O$ (2.5 mg, 0.010 mmol, 10 mol%) and ZnTPP (**4**, 6.8 mg, 0.010 mmol, 10 mol%). The vial was closed with a screw cap equipped with a septum and the atmosphere was evacuated and refilled with N_2 three times. Then, dry DMA (2.0 mL, 50 mM) was added, followed by piperidine (34 mg, 40 µL, 0.400 mmol, 4.0 equiv), before the cap was further sealed with parafilm. The vial was loaded into the photoredox set up (Kessil lamp and Hepatochem PhotoRedOx Duo^{TM}, see Section 2 for details) and it was irradiated with 660 nm red light upon stirring over 16 h. After that time, the mixture was diluted with 10 mL of water and 10 mL of EtOAc. The organic fraction was washed once with water and once with brine, dried over anhydrous Na_2SO_4, filtered and concentrated in vacuum. The resulting residue was purified by flash column chromatography in silica gel, using a gradient between pure hexane and hexane/EtOAc 95:5 as eluent. This gave the title product as a white solid (20 mg, 61%). Characterization data matched the previously reported ones for the product obtained from the same reaction under blue-light irradiation, without ZnTPP.[9]

Rf (95:5 hexane/EtOAc) = 0.5

^{1}H NMR (300 MHz, $CDCl_3$) δ 7.37 (d, *J* = 8.9 Hz, 2H), 6.93 (s, 4H), 6.82 (d, *J* = 8.9 Hz, 2H), 3.15 – 3.07 (m, 4H), 1.73 (dt, *J* = 11.4, 6.1 Hz, 4H), 1.58 (q, *J* = 6.1 Hz, 2H).

^{13}C NMR (75 MHz, $CDCl_3$) δ 157.97, 149.30, 148.80, 132.41, 120.50, 119.15, 118.03, 114.48, 51.34, 25.97, 24.19.

HRMS (ESI+): calculated for $C_{17}H_{19}{}^{79}BrNO$ $[M+H]^+$: 332.0645; found: 332.0647.

We also performed a variety of control or optimization experiments with the reaction, following the procedure above but at 0.025 mmol scale, and determining the yields by ^{1}H NMR. The obtained results are summarized in the following table:

Table S8: Control and optimization experiments for the red-light-promoted nickel-metallaphotoredox C–N coupling of aryl thianthrenium salts. Unless stated otherwise, yields were determined by ^{1}H NMR using 1,3,5-trimethoxybenzene as internal standard. TT = released thianthrene. n/d = not detected. n/dm = not determined.

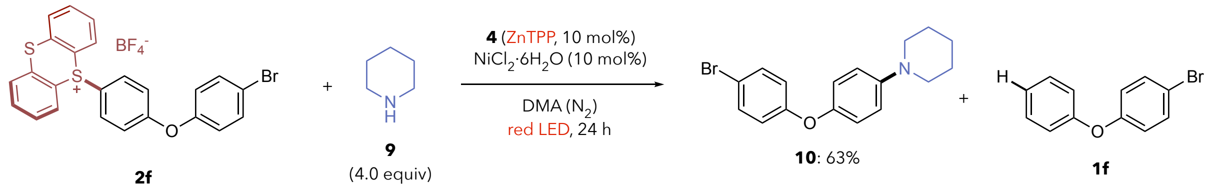

Entry	Deviations from above	Recov. **2f**	TT	**10**	**1f**
1	no deviations	<5%	82%	63% (61% isol)	39%
2	with **MB** instead of **ZnTPP**	>50%	50%	trace (<3%)	48%
3	with **PrDMQA-BF$_4$** instead of **ZnTPP**	>43%	<10%	n/d	<10%
4	without photocatalyst (MB)	>66%	n/d	n/d	n/d
5	without $NiCl_2{\cdot}6H_2O$	n/d	>95%	n/d	>95%
6	without light	>45%	n/d	n/d	n/d
7	under air instead of N_2	>29%	n/dm	n/d	28%

This reaction was found to be restricted to the use of ZnTPP as photocatalyst (Entries 2 and 3). Also, no exogenous reductive quencher is required, presumably due to the presence of an excess of piperidine which can also act as sacrificial electron donor as well as the coupling partner.

Notably, this metallaphotoredox reaction required the use of dry solvent and a protective inert atmosphere, which is consistent with the involvement of potentially air-sensitive organonickel intermediates (Entry 7). When the reaction was attempted in the absence of Ni catalyst (Entry 5) or in the presence of air (Entry 7), the only detectable product was that of deprotection (**1f**).

9. General considerations for cellular experiments

Unless stated otherwise, all procedures involving mammalian cell cultures were performed at room temperature on a laminar flow hood (HR1200-IIA2, Haier Biomedical), to maintain sterile conditions. Solutions stored in a fridge were warmed beforehand in a water bath (37 °C).

All procedures involving bacteria were carried out in close proximity to a Bunsen burner flame to maintain sterile conditions and reduce the risk of contamination. *B. cinerea* cultures and procedures were performed the same way, as only young cultures (white mycelia, with little or no sporulation) were used for the studies.

Mammalian cell cultures

HeLa and HepG2 cell lines were kindly provided by Dr. Bruno Sainz and Dr. Yolanda Pazos, respectively. Cells were maintained in Dulbecco´s modified Eagle medium (DMEM) supplemented with 100 U/mL penicillin, 100 µg/ml streptomycin, 2 mM L-glutamine and 10% of fetal bovine serum (FBS), at 37 °C in a humidified incubator with 5% CO_2. Cells were monthly tested for mycoplasma contamination and used only for 1–3 months after defrosted.

Bacterial growth conditions

Staphylococcus aureus and *Bacillus thuringiensis* were generously provided by Dr. Manuel Romero and Dr. Ana Otero, respectively. Bacteria were initially grown in Luria-Bertani (LB) agar medium at 37 ºC and maintained by periodic subculturing in the same media, by streaking a small portion of a single colony onto a new LB agar plate.

Fungal growth conditions

Botrytis cinerea (MUCL-16) was purchased from MUCL Agro-food & Environmental Fungal Collection (BCCM). The strain was initially grown in potato dextrose agar (PDA), at 20 ºC in light conditions, and periodically subcultured in the same media, by transferring 10 mm mycelial plugs to new PDA Petri dishes.

10. Cell viability experiments

10.1. Analysis of the effect of free boscalid vs TT-protected boscalid on HepG2 cell viability

To analyze the cytotoxic effect of free boscalid **1c** or TT-protected boscalid **2c**, HepG2 cells were seeded in a 96-well plate, at a cell density of 5 x 10^3 cells per well. 48 h after seeding, fresh stock solutions of **1c** and **2c** (40 mM in DMSO) were prepared and cells were incubated with different concentrations (25-200 μM) of the above-mentioned compounds, diluted in DMEM supplemented with 10% FBS. DMSO concentration was equalized in all the conditions tested, to avoid differences caused by solvent effects. Cytotoxicity assay was carried out 24 h after treatment, by using 3-(4,5-dimethylthiazol-2-yl)-2,5-diphenyltetrazolium bromide (MTT) colorimetric assay (Sigma-Aldrich).

Cells were incubated with MTT solution at 37 ºC for 2 h, at a final concentration of 0.5 mg/mL diluted in DMEM supplemented with 10% FBS. After incubation with MTT solution, culture medium was carefully removed, and formazan crystals were dissolved with DMSO. Finally, absorbance was measured at a wavelength of 570 nm, using 630 nm as a reference value. Absorbance was measured by using Infinite® 200 PRO plate reader (Tecan). Results are expressed as mean ± SD of three biological replicates, each one with 6 technical replicates. Statistical significance of data was determined with one-way ANOVA statistical test (*p < 0.05; ** p < 0.01; *** p < 0.001; ****p<0.0001), using GraphPad Prism software.

10.2. Analysis of the effect of different photocatalysts under light irradiation on the viability of mammalian cells

In order to select the best photocatalytic system to perform uncaging experiments in cellulo, we evaluated the phototoxicity of a variety of green- and red-light based photoredox manifolds.

To analyze the effect of each system on cell viability, HeLa cells were seeded in a 96-well plate, at a cell density of 5 x 10^3 cells per well. 24 h after seeding, fresh stock solutions (10 mM in DMSO) were prepared and cells were incubated for 15 minutes with increasing concentrations (2.5 to 10 µM) of each photocatalyst diluted in DMEM without FBS or antibiotics, since that is how it used for the reaction. An equal amount of DMSO was used in all the conditions tested, to avoid differences caused by solvent effects. Once incubation finished, medium was changed by DMEM without phenol red, and cells were irradiated for 30 minutes with green light (525 nm) in case of Eosin Y, rhodamine B and fluorescein, or red light (660 nm) in case of methylene blue. After irradiation, fresh culture media was added, and cytotoxicity assay was carried out after overnight recovery, by using MTT colorimetric assay as described in Section 10.1.

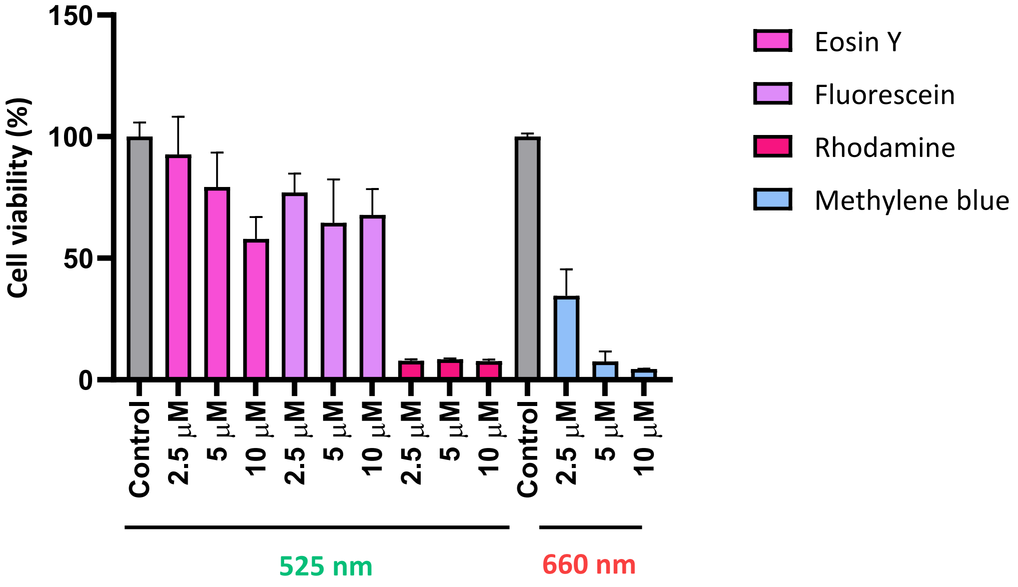

Figure S28. Cytotoxic effect of different photocatalysts under irradiation in HeLa cells. Cells were treated with increasing concentrations of Eosin Y, rhodamine and fluorescein for 15 minutes, and irradiated with green or red light as appropriate. DMSO was used as a vehicle control. Cell viability was measured by means of MTT assay. Results are represented as mean ± SD of six technical replicates.

While methylene blue and rhodamine B displayed a very significant cell toxicity under light irradiation at the corresponding wavelength, the combination of fluorescein and especially Eosin Y under green-light irradiation proved to be much more innocuous to mammalian HeLa cells under the evaluated conditions. Furthermore, we have already established Eosin Y as a viable photocatalyst to perform intracellular photocatalysis in a previous study. Quantification studies allowed proving successful internalization of this photocatalyst within mammalian cells.[10]

Taking all this together, we selected Eosin Y and 525 nm light as model system to explore the intracellular uncaging reactions. For a parallel comparison between different photocatalytic systems, see Section 12.3.

10.3. Effect of the uncaging reaction conditions on HepG2 cell viability

We also performed MTT cell-viability studies under the exact reaction conditions used for the uncaging of boscalid–TT **2c** before extraction and HPLC analysis (see Section 12).

The effect of intracellular photoredox C–H uncaging of boscalid–TT (**2c**) on cell viability was evaluated by seeding HepG2 cells in a 96 well plate, at a cell density of 5 x 10^3 cells per well. Two days after seeding, fresh stocks solutions (40 mM for **2c** and 10 mM for EY **3**) were prepared in DMSO and cells were incubated for 15 minutes with 100 µM **2c** and 15 µM Eosin Y (**3**) diluted in DMEM. The same amount of DMSO was used in each treatment, to avoid differences caused by the solvent itself. After incubation, medium was changed, and cells were irradiated for 1 hour with green light (525 nm). Appropriate controls were included, such as cells incubated with Eosin Y only, **2c** only, or without any compound, both with and without irradiation. After irradiation (or after 1 hour in dark conditions in control plates), fresh culture media was added, and cytotoxicity assay was carried out, by using MTT colorimetric assay as described in Section 10.2.

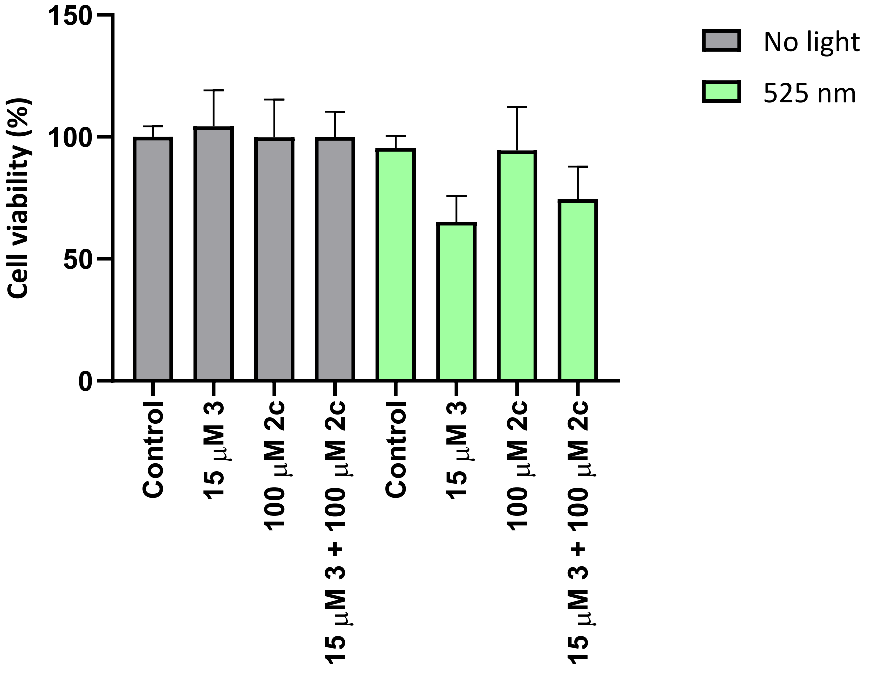

Figure S29. Cytotoxic effect of intracellular photoredox C–H uncaging of boscalid–TT (**2c**). HepG2 cells were treated with 15 µM **3** and 100 µM **2c** for 15 minutes and then irradiated with green light (or left in dark) for 1 hour. DMSO was used as a solvent control. Controls with **3** or **2c** alone are included. Cell viability was measured using MTT assay. Results are represented as mean ± SD of six technical replicates.

None of the reaction components were found to be cytotoxic at the employed concentrations in the dark. Under light irradiation, some phototoxicity was observed steaming from the photocatalyst (**3**). Interestingly, this toxicity marginally decreased in the presence of boscalid thianthrenium salt **2c**, possibly due to quenching of the excited/reduced photocatalyst, minimizing ROS generation.

10.4. Effect of thianthrene in HepG2 cell viability

To evaluate the potential contribution of thianthrene release to the cytotoxic effect of the unmasking reaction, HepG2 cells were seeded in a 96-well plate at a density of 1×10^4 cells per well. The following day, a fresh thianthrene stock solution (40 mM in DMSO) was prepared, and cells were incubated for 15 minutes with increasing concentrations (1–200 μM) of thianthrene (TT) diluted in DMEM. The final DMSO concentration was kept constant across all conditions to avoid effects caused by the solvent itself. After incubation, the medium was replaced, and cell viability was evaluated using the MTT colorimetric assay (Sigma-Aldrich), as described in Section 10.2.

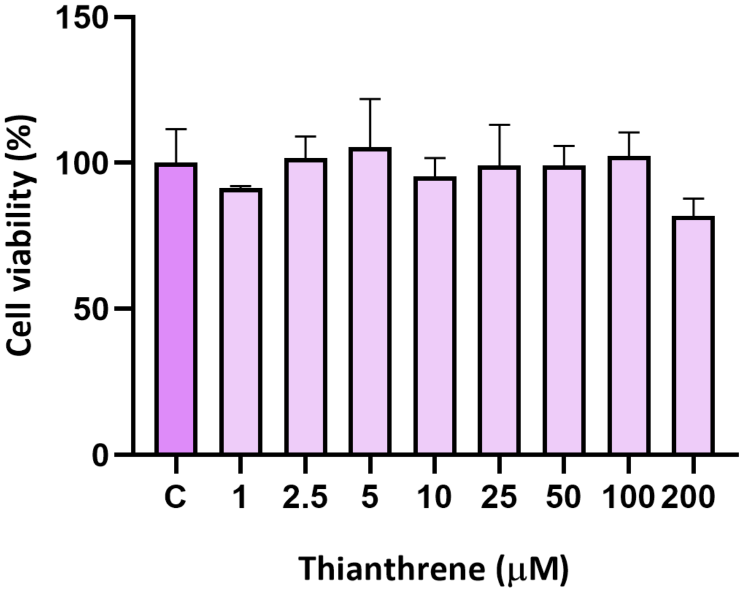

Figure S30. Cytotoxic effect of free thianthrene (TT). HepG2 cells were treated for 15 min with increasing concentrations of free thianthrene (TT), maintaining a constant DMSO concentration across conditions. Cell viability was subsequently determined by MTT assay. Results are expressed as mean ± SD of two biological replicates, each one with 6 technical replicates.

None of the tested concentrations of thianthrene (TT) significantly affected cell viability of HepG2 cells after 15 min of incubation, suggesting that thianthrene release during the unmasking reaction would not cause any cytotoxic effects.

10.5. Effect of TPE–TT AIE probe in HeLa cell viability

To evaluate whether the internalization of TPE–TT **2g** AIE probe induced cytotoxic effects in HeLa cells, an MTT assay was performed. Cells were seeded in a 96-well plate at a cell density of 1×10^4 cells per well. On the following day, a fresh **2g** stock solution (10 mM in DMSO) was prepared, and cells were incubated for 15 minutes with 10 μM TPE–TT diluted in DMEM. Same amount of DMSO was used for the control condition, to avoid differences caused by the solvent itself. After incubation, the medium was replaced, and cell viability was assessed using the MTT colorimetric assay (Sigma-Aldrich) as described in Section 10.2.

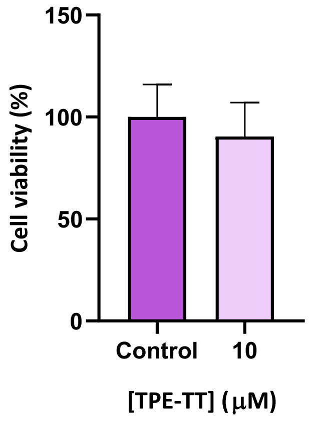

Figure S31. Effect TPE–TT **2g** AIE probe in cell viability. HeLa cells were treated for 15 minutes with 10 μM TPE–TT **2g** or DMSO as a solvent control, and cell viability was subsequently analyzed using MTT assay. Results are represented as mean ± SD of six technical replicates.

11. Cell internalization and fluorescence microscopy with TPE–TT

11.1. Internalization of AIE probes in mammalian HeLa cells

The experiments were performed on 24-well glass-bottom plates as follows: 125,000 cells per well were seeded two days before treatment. The following conditions reflect the optimized in cellulo protocol: Culture medium was replaced by 300 μL of DMEM containing thianthrenium salt TPE–TT **2g** (5-25 μM) or the uncaged product **1g** (5-25 μM). After 15 min of incubation, cells were washed twice with fresh PBS and then treated with DMEM (300 μL). The cells were observed under microscope with adequate filters. Digital pictures of the samples were taken and processed under identical conditions of gain and exposure.

Fluorescence microscopy for HeLa cells: Fluorescence and brightfield images were taken using a Nikon Eclipse Ti microscope equipped with a Zyla camera, using a 100×/1.45 oil immersion objective. Images were processed with NIS software and analyzed with ImageJ using identical brightness and contrast settings across samples.

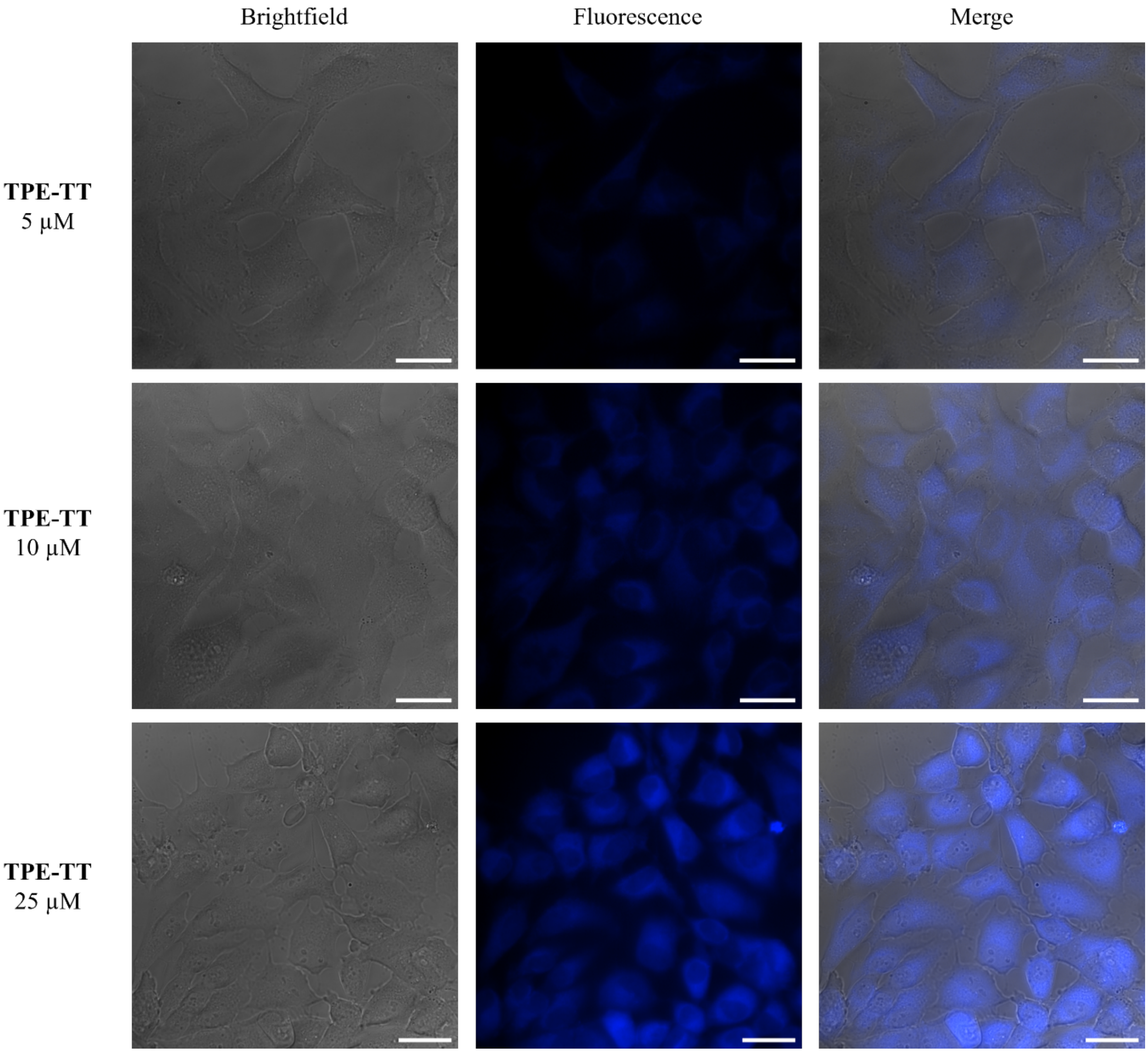

Figure S32. Fluorescence micrographs of different internalization experiments in HeLa cells after incubation with TPE-TT **2g**. Reaction conditions: Cells were incubated with different concentrations of TPE-TT **2g** for 15 min and then washed twice with PBS. Scale bar: 30 μm. λ_{exc} = 365 nm, λ_{em} = DAPI channel.

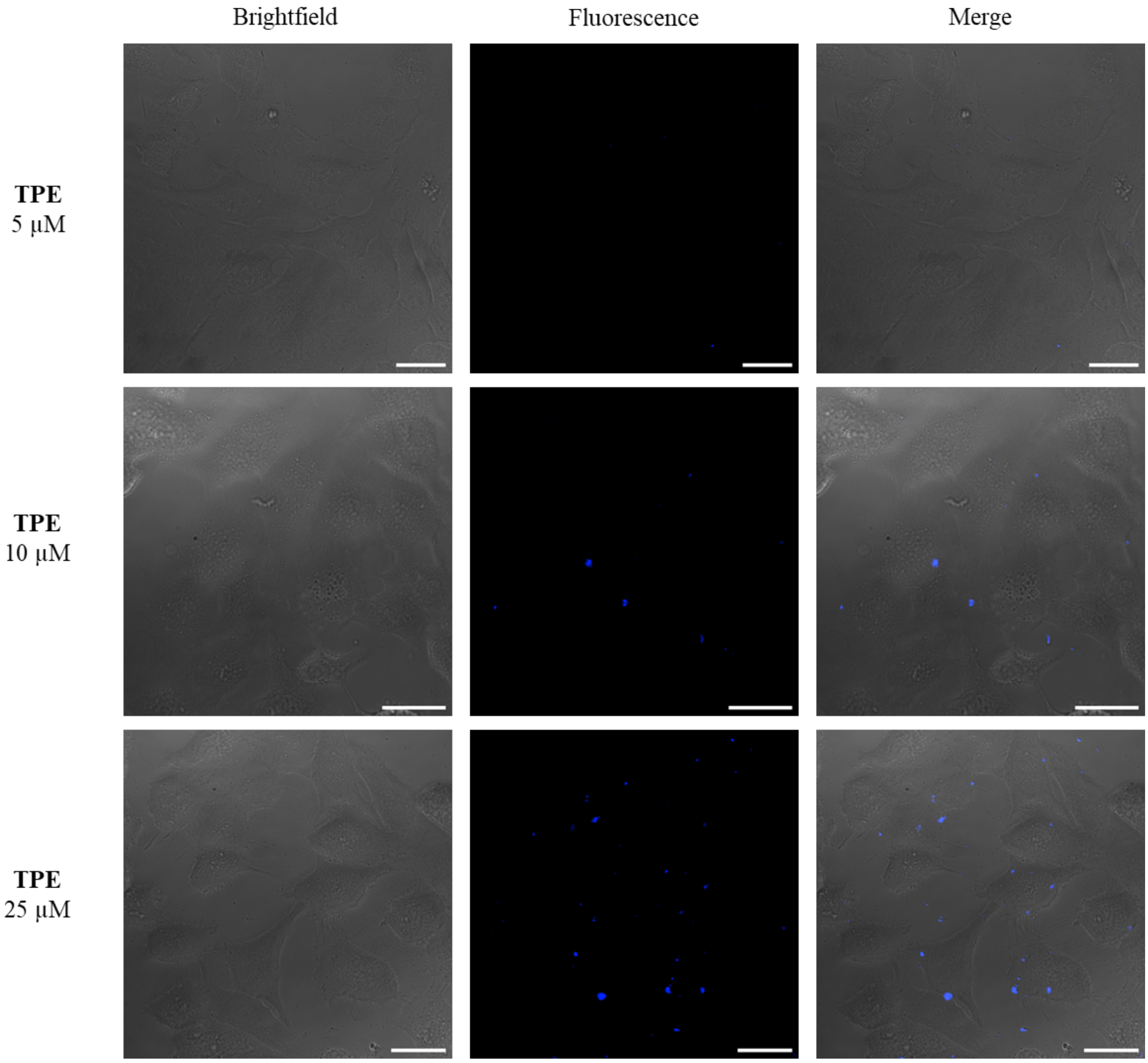

Figure S33. Fluorescence micrographs of different internalization experiments in HeLa cells after incubation with TPE **1g**. Reaction conditions: Cells were incubated with different concentrations of TPE **1g** for 15 min and then washed twice with PBS. Scale bar: 30 µm. λ_{exc} = 365 nm, λ_{em} = DAPI channel.

11.2. Bacterial internalization of AIE probes

To study the differential internalization of TPE **1g** vs TPE–TT **2g**, a single colony of *S. aureus* or *B. thuringiensis* was grown at 37 ºC overnight in 10 mL LB culture media. The day after, OD_{600} was measured, and bacterial cells were washed and resuspended in PBS to a final OD_{600} = 1. Then, bacterial cells were incubated with 50 µM TPE **1g** or TPE–TT **2g** for 15 minutes at 37ºC. After incubation, cells were centrifuged to remove treatment and resuspended in PBS. Finally, 10 µL of each bacterial suspension were placed onto a slide and subsequently analyzed by epi-fluorescence microscopy. TPE was also analyzed without removing the supernatant after incubation to observe the precipitates outside the bacterial cells.

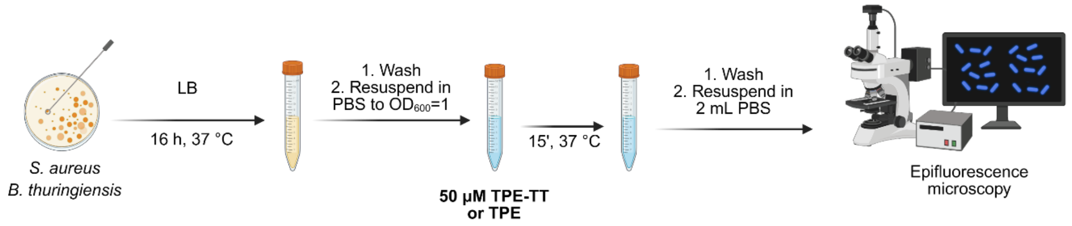

Figure S34. Schematic representation of bacterial internalization of AIE probes. Bacterial cells (OD_{600} = 1) were incubated for 15 min with **1g** or **2g**, washed and analyzed by epifluorescence microscopy.

Fluorescence microscopy for bacteria: Fluorescence and brightfield images were taken using a Nikon Eclipse Ti microscope equipped with a Sona 6 camera, using a 100×/1.45 oil immersion objective. Images were processed with Fusion software (version 2.4.0.14) and analyzed with ImageJ using identical brightness and contrast settings across samples.

A. *Bacillus thuringiensis*

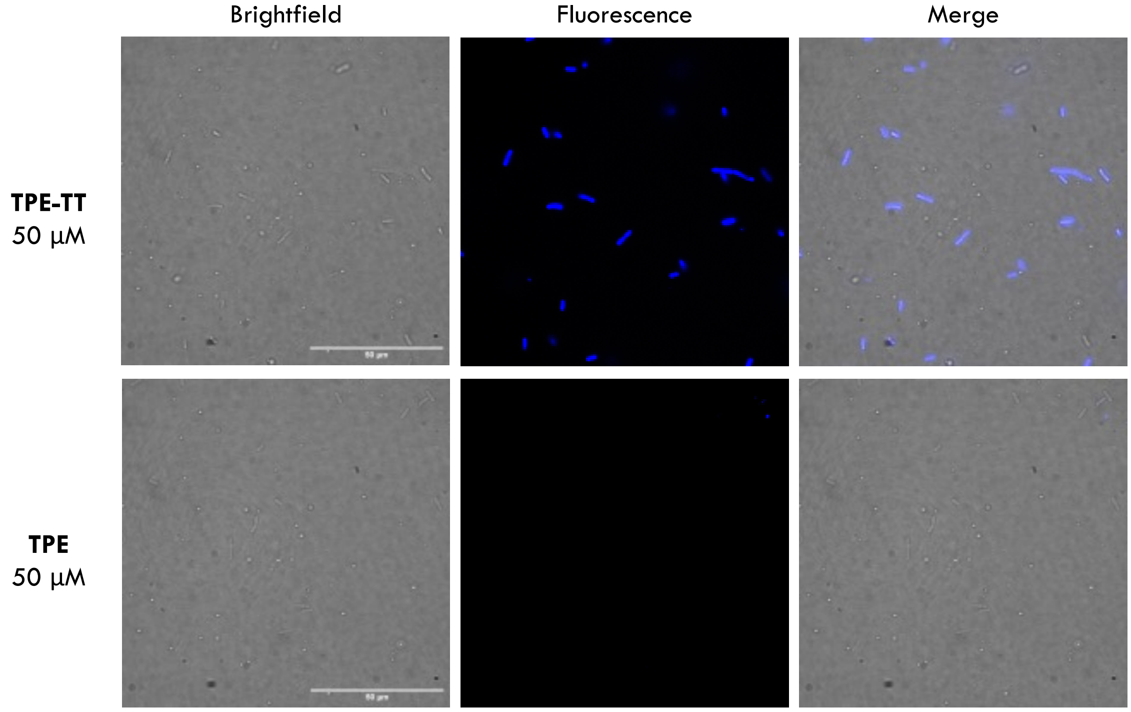

Figure S35. Fluorescence and brightfield images of *B. thuringiensis* after incubation for 15 min with 50 µM TPE–TT **2g** or TPE **1g**. Scale bar: 50 µm. λ_{exc} = 385 nm, λ_{em} = DAPI channel**.**

Bacillus thuringiensis: Incubation of TPE without washing step, observing only extracellular precipitate.

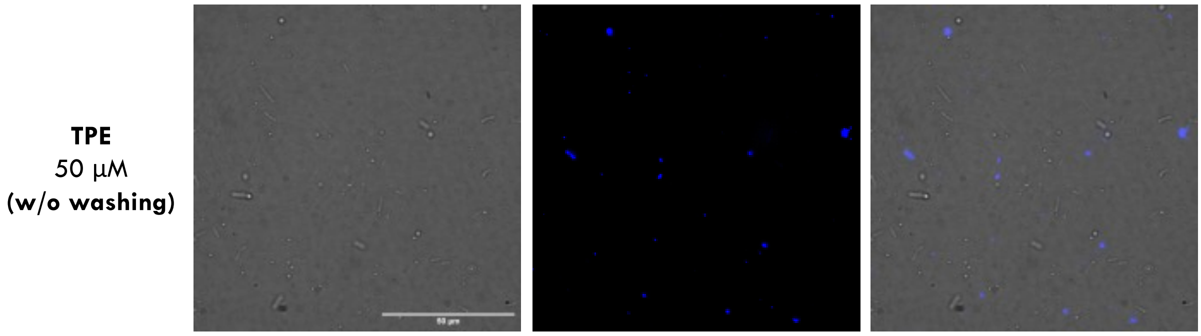

Figure S36. Fluorescence and brightfield images of *B. thuringiensis* after incubation for 15 min with 50 µM TPE **1g** without washing. Scale bar: 50 µm. λ_{exc} = 385 nm, λ_{em} = DAPI channel.

B. *Staphylococcus aureus*

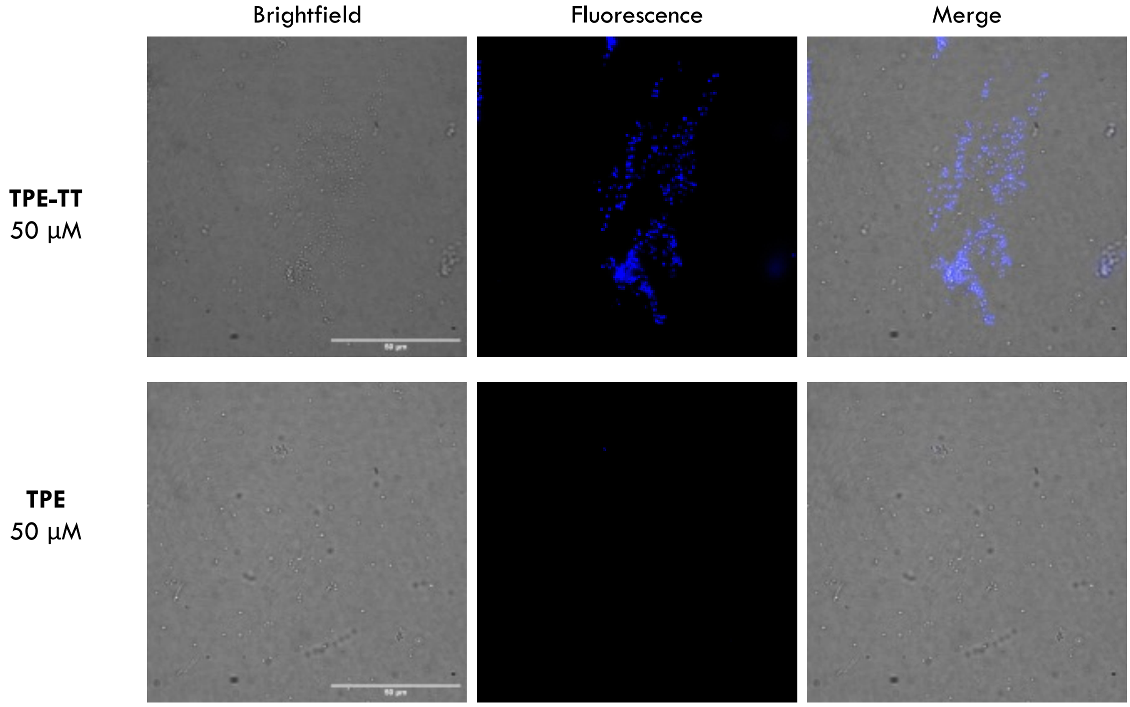

Figure S37. Fluorescence and brightfield images of *S. aureus* after incubation for 15 min with 50 µM TPE–TT **2g** or TPE **1g**. Scale bar: 50 µm. λ_{exc} = 385 nm, λ_{em} = DAPI channel.

12. Delivery and photocatalytic uncaging in cell cultures

12.1. General procedure C for the intracellular reactions in HepG2 cells

To evaluate the viability of intracellular photoredox C–H uncaging of boscalid–TT **2c**, HepG2 cells were seeded in 100 mm dishes at a cell density of 1.5 x 10^6 cells per dish. Two days after seeding, cells were incubated with masked boscalid–TT **2c** (100 µM) and Eosin Y **3** (15 µM) for 15 minutes in DMEM. Prior to irradiation, incubation medium was removed and replaced with fresh DMEM, and cells were subsequently irradiated for 1 hour with green light (525 nm). Reactions without catalyst or without light were performed as a control.

After this time, the reaction media was collected in a 15 mL Falcon tube (Figure S38, **A**). Then, cells were washed with 3 mL of PBS, and this washing media was also collected separately in another 15 mL Falcon tube (Figure S38, **B**). Then, the cell monolayer was extracted with MeCN (3x1 mL). The combined MeCN extracts were subsequently concentrated and analyzed by HPLC–MS to determine intracellular contents (Figure S38, **C**). Separately, the reaction media (**A**) and the PBS washing media (**B**) were also lyophilized and analyzed by HPLC–MS to determine extracellular contents. After lyophilization, each of the three samples (**A**, **B** and **C**) were dissolved in 1 mL of MeCN and filtered through Whatman® syringe filters (0.45 µm pore size). Subsequently, 200 µL of the filtered solution were transferred to an Eppendorf tube, and caffeine (used as an internal standard with a stock concentration of 1 mM in CH_3CN) was added to achieve a final concentration of 20 µM.

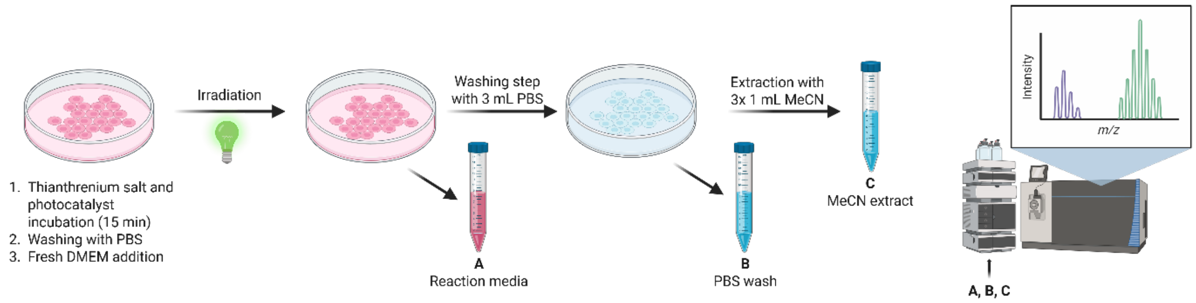

Figure S38. Schematic representation of the protocol for extraction and quantification of the photochemical C–H unmasking reaction in mammalian cells.

For the analysis, the samples were injected into an Agilent 1260 Infinity II system connected to an Agilent Technologies 6120 Quadrupole LC-MS, using a Phenomenex Luna-C18 reverse-phase column (250 x 10 mm) and a flow rate of 0.35 mL/min at room temperature. Solvent systems based on a gradient between A (H_2O with 0.1% formic acid) and B (CH_3CN 0.1% formic acid). The program started with 40% of B and gradually transitioned to 95% B over 12 minutes. After each analysis, the initial conditions were then gradually reinstated over a period of one minute and maintained for two minutes. Chromatograms were recorded using MS detection.

12.2. Quantification of caged and uncaged boscalid from HepG2 cells extracts

Each sample was analyzed by HPLC–MS in order to determine the concentration of boscalid **1c** formed and of boscalid–TT **2c** remaining. These concentrations were determined using the calibration curves below and then normalized to the total amount of cells in each well (nmol/10^6 cells).

For the calibration curve, we represented the analyte peak area ratio obtained through *Extracted Ion Chromatogram* HPLC spectra versus the concentration. To build the calibration curves, stock solutions of each analyte were prepared at different concentrations (10–200 nM for boscalid **1c** and 50–1500 nM for boscalid–TT **2c**) in MeCN/water, each containing a fixed concentration of internal standard (caffeine, 20 µM). Samples were analyzed using the chromatographic method described in Section 12.1.

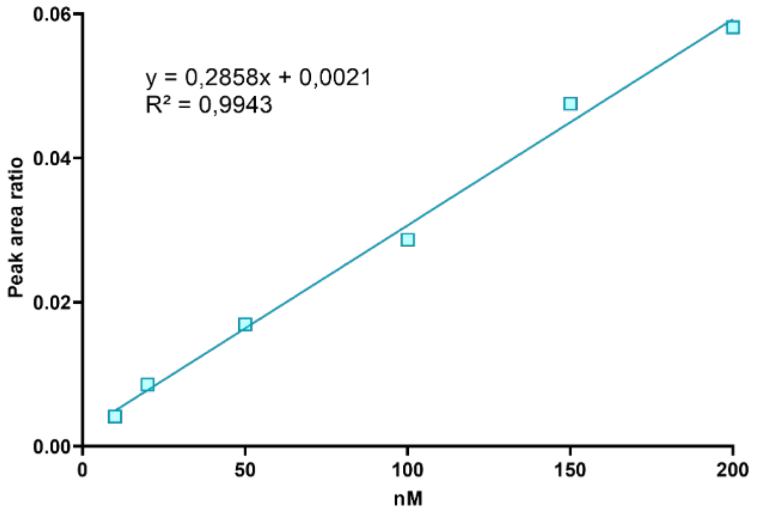

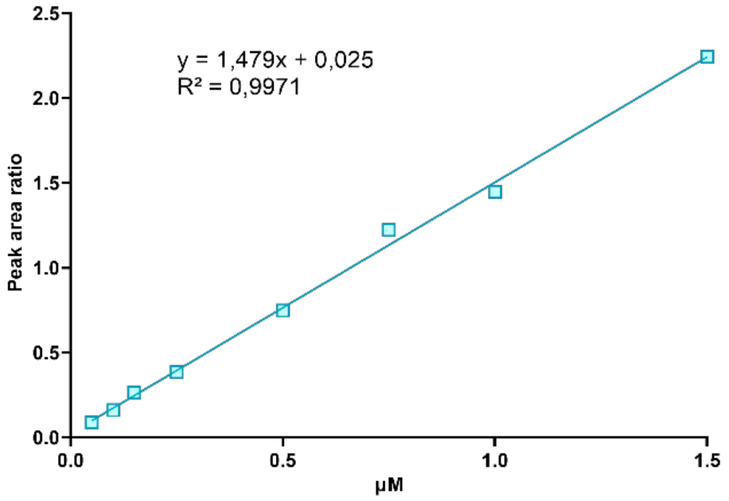

Figure S39. Top: calibration curve of boscalid **1c** (10–200 nM); Bottom: calibration curve of boscalid–TT **2c** (50 nM–1500 nM).

12.3. Intracellular photoredox uncaging reaction results

Representative results obtained after 1 hour of reaction using Eosin Y as photocatalyst

Each plate was analyzed and the amount of boscalid **1c** (nmol/10^6 cells) formed was calculated using the calibration curve. As a representative example, we show here the experiments with Eosin Y (15 µM) and boscalid–TT **2c** (100 µM) irradiated with a Green Kessil PR160 at 15 cm of distance for 1 h.

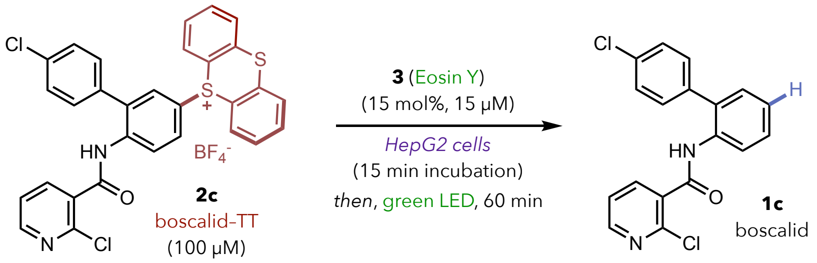

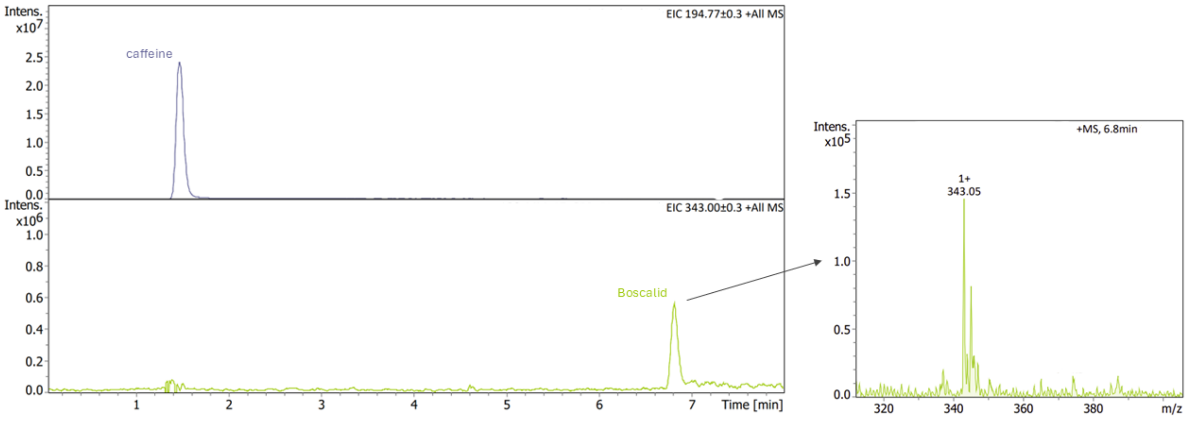

Figure S40. Detection and quantification of boscalid **1c** in HepG2 cells (MeCN extract). *Extracted Ion Chromatogram*: in blue, caffeine peak (internal standard); in green: boscalid **1c** and inset with the mass spectrum corresponding to the boscalid peak. Results obtained when using 15 µM of Eosin Y and 100 µM of boscalid–TT **2c**.

The results shown in Figure 5 of the main text, and all the Supplementary Figures displayed below were obtained following General Procedure C, with the corresponding variations.

Evaluation of different photocatalysts

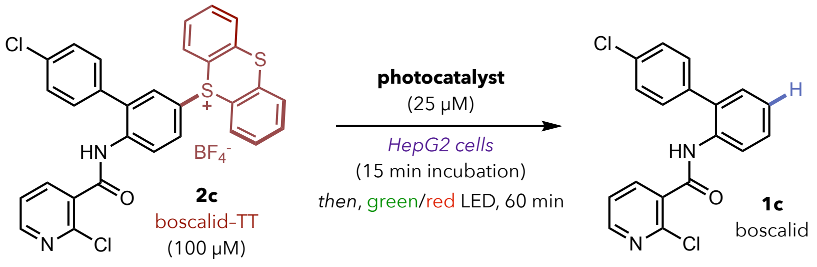

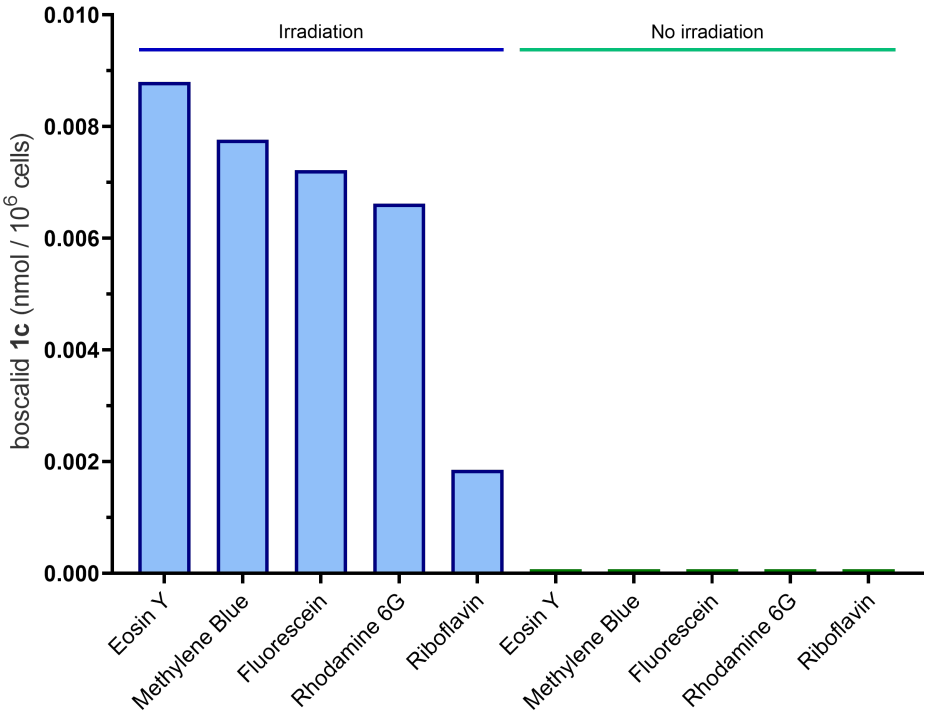

Figure S41. Screening of different photocatalysts (25 µM) using boscalid-TT **2c** (100 µM). Left bars: Results obtained under irradiation with a 525 nm Kessil lamp (the methylene blue sample was irradiated at 660 nm). Right bars: Results obtained in the absence of irradiation. The results shown in this figure correspond to experiments performed in parallel in the same day in 10 different plates.

Evaluation of different loadings of Eosin Y and cell confluence

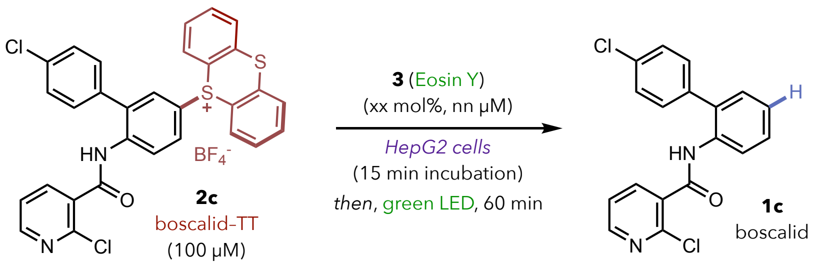

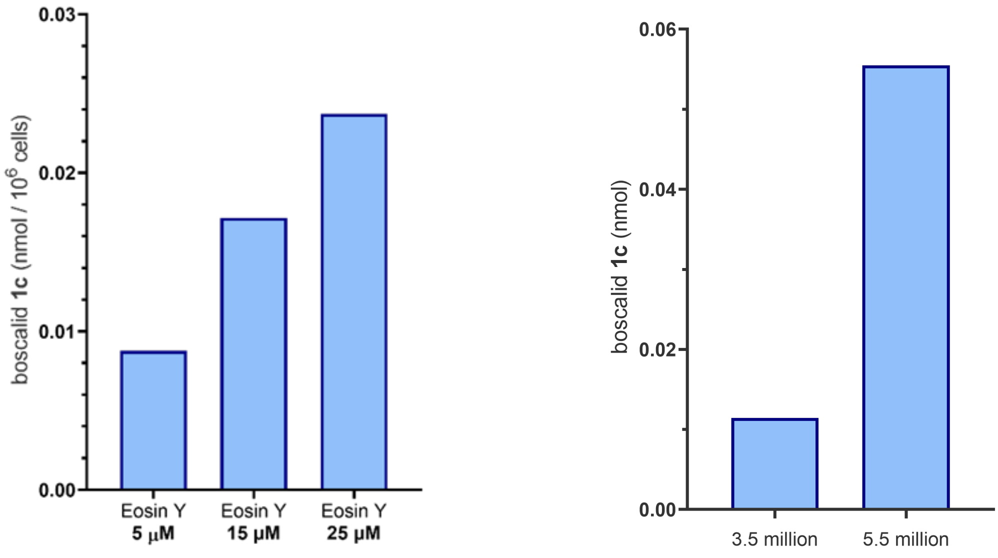

Figure S42. Left: screening of different Eosin Y loadings (5-25 µM) when using 100 uM of boscalid-TT **2c**; Right: comparison between different cell confluence, results obtained when using 15 µM of Eosin Y and 100 µM of boscalid–TT **2c**. The results shown in this figure correspond to experiments performed in parallel in the same day in 5 different plates.

Results of analysis of intracellular extracts and extracellular media: quantification of boscalid-TT 2c and boscalid 1c in acetonitrile extracts

Table S9. Uncaging reaction. Quantification of boscalid **1c** in HepG2 cells, after incubation with boscalid-TT **2c** (100 μM) and Eosin Y (15 μM) for 15 minutes and irradiation with green light for 1 hour**.** Analysis of intracellular content **A** + **B** + **C** of three experiments performed in different plates on different days, with cells coming from the same culture.

Sample	Value (nmol)	Number of cells	Normalization (nmol / 10^6 cells)
1	$4.35 \cdot 10^{-2}$	$2.90 \cdot 10^{6}$	$1.49 \cdot 10^{-2}$
2	$5.95 \cdot 10^{-2}$	$4.48 \cdot 10^{6}$	$1.33 \cdot 10^{-2}$
3	$8.14 \cdot 10^{-2}$	$5.05 \cdot 10^{6}$	$1.61 \cdot 10^{-2}$
		**Average**	$1.48 \cdot 10^{-2} \pm 1.40 \cdot 10^{-3}$

Table S10. Uncaging reaction. Detection of boscalid-TT **2c** in HepG2 cells, after incubation with boscalid-TT **2c** (100 μM) and Eosin Y (15 μM) for 15 minutes and irradiation with green light for 1 hour**.** Analysis of intracellular content **C** of three experiments performed on different days, with cells coming from the same culture.

Sample	Value (nmol)	Number of cells	Normalization (nmol / 10^6 cells)
1	4.08	$7.31 \cdot 10^{6}$	0.56
2	0.82	$5.05 \cdot 10^{6}$	0.16
3	1.58	$5.02 \cdot 10^{6}$	0.31
		**Average**	0.34 ± 0.19

According to these values, we determined an intracellular ratio of 4.4% between uncaged **1c** and recovered unreacted boscalid–TT **2c**.

Table S11. Control without Eosin Y. Quantification of boscalid **1c** in HepG2 cells, after incubation with boscalid-TT **2c** (100 µM) for 15 minutes and irradiation with green light for 1 hour, in absence of photocatalyst. Analysis of intracellular content **C** of three experiments performed on different days, with cells coming from the same culture. A very low-intensity peak was detected in both controls, yet its signal was near the noise threshold and outside the calibrated range.

Sample	Value (nmol)	Number of cells	Normalization (nmol / 10^6 cells)
1	traces	$5.05 \cdot 10^6$	traces
2	traces	$4.48 \cdot 10^6$	traces
3	traces	$5.02 \cdot 10^6$	traces

Table S12. Control without light. Quantification of boscalid **1c** in HepG2 cells, after incubation with boscalid-TT **2c** (100 µM) and Eosin Y (15 µM) for 15 minutes and exposition to ambient light for 1 hour**.** Analysis of intracellular content **C** of three experiments performed on different days, with cells coming from the same culture. A very low-intensity peak was detected in both controls, yet its signal was near the noise threshold and outside the calibrated range.

Sample	Value (nmol)	Number of cells	Normalization (nmol / 10^6 cells)
1	traces	$5.05 \cdot 10^6$	traces
2	traces	$4.48 \cdot 10^6$	traces
3	traces	$5.02 \cdot 10^6$	traces

Table S13. Quantification of boscalid **1c** detected in the different fractions **A**, **B** and **C**, in HepG2 cells, after incubation with boscalid-TT **2c** (100 µM) and Eosin Y (15 µM) for 15 minutes and irradiation with green light for 1 hour, in an experiment performed in one plate.

Sample	Normalization (nmol / 10^6 cells)
Reaction media **A**	$5.44 \cdot 10^{-3}$
Washing **B**	$2.59 \cdot 10^{-3}$
Extraction **C**	$8.07 \cdot 10^{-3}$
Total	$1.61 \cdot 10^{-2}$

Table S14. Quantification of boscalid **1c** formed in HepG2 cells after incubation with boscalid-TT **2c** (100 µM) and the indicated photocatalyst (see table) for 15 min, followed by irradiation with green light for 1 h. Analysis of intracellular content **A** + **B** + **C** with cells coming from the same culture. A very low-intensity peak was detected in non-irradiated samples, yet its signal was near the noise threshold and outside the calibrated range.

Photocatalyst	Value (nmol)	Number of cells	Normalization (nmol / 10^6 cells)
Eosin Y	$4.38 \cdot 10^{-1}$	$4.98 \cdot 10^{6}$	$8.79 \cdot 10^{-3}$
Methylene blue	$3.86 \cdot 10^{-1}$	$4.98 \cdot 10^{6}$	$7.76 \cdot 10^{-3}$
Fluorescein	$3.59 \cdot 10^{-2}$	$4.98 \cdot 10^{6}$	$7.22 \cdot 10^{-3}$
Rhodamine 6G	$3.29 \cdot 10^{-2}$	$4.98 \cdot 10^{6}$	$6.61 \cdot 10^{-3}$
Riboflavin	$3.91 \cdot 10^{-3}$	$4.98 \cdot 10^{6}$	$7.87 \cdot 10^{-4}$

Table S15. Uncaging reaction. Quantification of boscalid **1c** formed in HepG2 cells after incubation with boscalid-TT **2c** (100 µM) and varying concentrations of Eosin Y (see table) for 15 min, followed by irradiation with green light for 1 h. Analysis of intracellular content **A** + **B** + **C** with cells coming from the same culture. A very low-intensity peak was detected in non-irradiated samples, yet its signal was near the noise threshold and outside the calibrated range.

Eosin Y loading (%)	Value (nmol)	Number of cells	Normalization (nmol / 10^6 cells)
5	$8.77 \cdot 10^{-3}$	$5.50 \cdot 10^{6}$	$8.77 \cdot 10^{-3}$
15	$1.72 \cdot 10^{-2}$	$5.50 \cdot 10^{6}$	$1.72 \cdot 10^{-2}$
25	$2.37 \cdot 10^{-2}$	$5.50 \cdot 10^{6}$	$2.37 \cdot 10^{-2}$

Table S16. Uncaging reaction. Quantification of boscalid **1c** formed in HepG2 cells at different cell confluencies after incubation with boscalid-TT **2c** (100 µM) and Eosin Y (15 µM) for 15 min, followed by irradiation with green light for 1 h Analysis of intracellular content **A** + **B** + **C** with cells coming from the same culture. A very low-intensity peak was detected in non-irradiated samples, yet its signal was near the noise threshold and outside the calibrated range.

Number of cells	Value (nmol)
$3.40 \cdot 10^{6}$	$1.11 \cdot 10^{-2}$
$5.50 \cdot 10^{6}$	$5.55 \cdot 10^{-2}$

Amount of uncaged boscalid in concentration units relative to the total amount of solvent

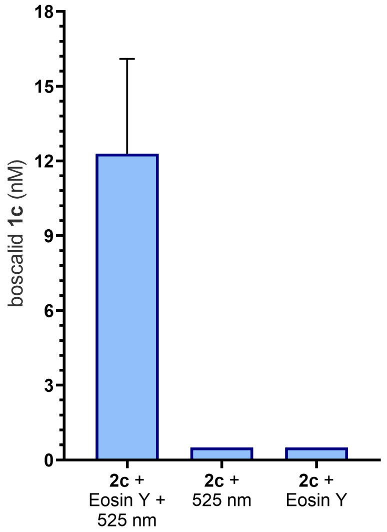

Figure S43. C–H release of boscalid inside live HepG2 cells: quantification of uncaged product by HPLC–MS in concentration units relative to the total amount of 5 mL of solvent. A minor product peak was detected in both controls but fell below the calibrated range and could not be reliably integrated.

Importantly, these concentration values correspond only to the amount of uncaged product **1c** formed *intracellularly* relative to the total amount of solvent in the plate, and cannot be directly compared to the initial dose and concentration of **2c** (since most of it is not internalized and therefore removed during washing steps).

13. Fungal culture experiments

13.1. Experiments of exposure of *B. cinerea* cultures to masked and free boscalid

Disc diffusion susceptibility test

The analysis of the antifungal activity of masked and unmasked boscalid was carried out by using a disc diffusion assay. To do so, *Botrytis cinerea* was grown on Potato Dextrose Agar (PDA, Sigma-Aldrich) plates for 10 days at 20 ºC under light. For the disc diffusion assay, blank antimicrobial paper discs embedded with the tested compounds were used. To prepare the treatment discs, compounds **1c** and **2c** were prepared at a stock concentration of 5 mg/mL in DMSO, and 50 and 100 µg of each compound were added to the discs. For the control discs, an equal volume of DMSO was used.

Once discs were air-dried, 10 mm mycelial plugs of the above-mentioned 10-day culture of *B. cinerea* were placed in the center of each plate, and embedded discs were placed around it. Plates were then incubated at 20 ºC, and growth inhibition was evaluated after 10 days of culture.

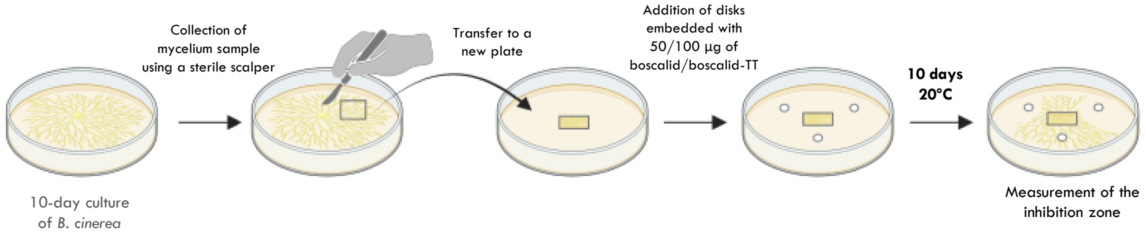

Figure S44. Botrytis cinerea disc diffusion assay workflow. 10 mm mycelial plugs of 10-day culture of *B. cinerea* and discs containing 50 and 100 µg of **1c** or **2c** (or DMSO as a control) were placed on a fresh PDA culture plate. Growth inhibition was analyzed after 10 days.

13.2. Experiments of exposure of *B. cinerea* cultures to unmasking reactions

In vitro reactions evaluated by fungal growth inhibition

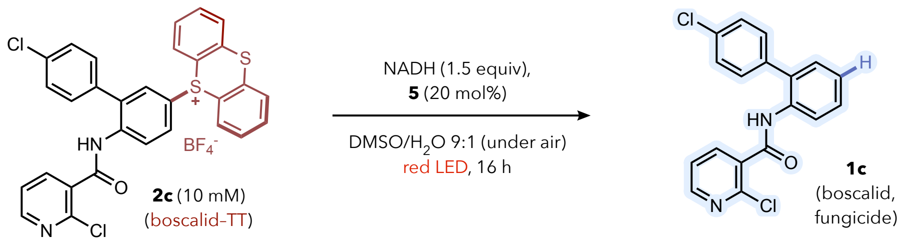

In order to evaluate the effectiveness of our photo-uncaging reaction to restore the antifungal properties of boscalid, we performed the reaction (and the corresponding control experiments) in vitro, and subsequently, we treated *B. cinerea* directly with the corresponding reaction crudes. The in vitro reactions were carried out following a procedure similar to the one described in Section 4.

Under air atmosphere, a 6 mL screw-cap glass vial equipped with a Teflon-coated magnetic stirring bar was charged with protected boscalid **2c** (6.5 mg, 0.010 mmol, 1.0 equiv), NADH (10.6 mg, 0.015 mmol, 1.5 equiv) and methylene blue (1.4 mg, 0.0020 mmol, 20 mol%). Everything was dissolved in a 9:1 mixture of DMSO/water (1.0 mL, 10 mM). Then, the vial was closed with the corresponding cap and loaded into the photoredox set up (Kessil lamp and Hepatochem PhotoRedOx Duo^{TM}, see Section 2 for details). Then, the vial was irradiated at 660 nm upon stirring over 16 h. After this time, irradiation was switched off and the mixture was directly loaded into blank antimicrobial paper discs as described below.

The corresponding control experiments were run omitting the corresponding component: either the photocatalyst, NADH or light.

Exposure of *B. cinerea* to the unmasking reaction mixtures

The antifungal effect of the boscalid–TT **2c** unmasking reaction crudes were analyzed by a disc diffusion assay. To do so, *Botrytis cinerea* was grown on Potato Dextrose Agar (PDA, Sigma-Aldrich) plates for 10 days at 20 ºC under light. For the disc diffusion assay, blank antimicrobial paper discs embedded with 20 µL (at 10 mM concentration) of each reaction mixture were added to the plates. 20 µL of 10 mM masked boscalid **2c** diluted in DMSO or 20 µL of DMSO were also added as a control. Discs embedded with crudes from control reactions (without light, without photocatalyst or without NADH) were also included as negative controls.

Once discs were air-dried, 10 mm mycelial plugs of the above-mentioned 10-day culture of *B. cinerea* were placed in the center of each plate, and embedded discs were placed around it. Plates were then incubated at 20 ºC, and growth inhibition was evaluated after 10 days of culture.

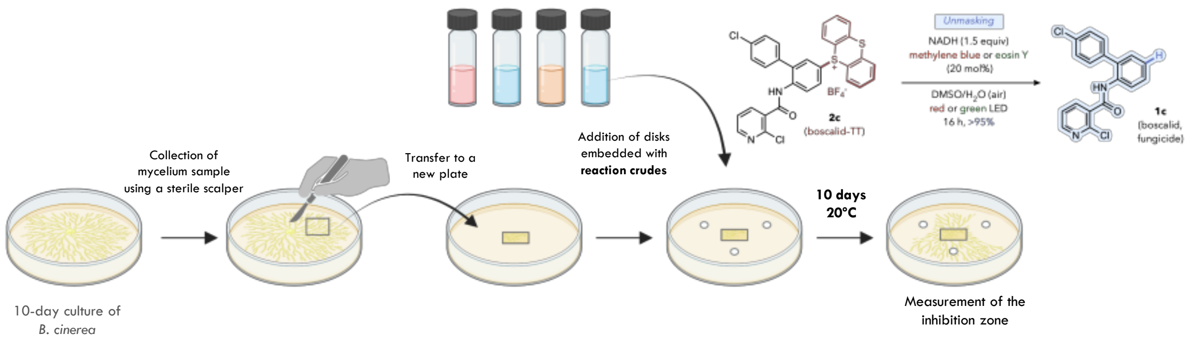

Figure S45. *Botrytis cinerea* disc diffusion assay workflow. 10 mm mycelial plugs of 10-day culture of *B. cinerea* and discs containing crudes from unmasking and control reactions (or DMSO or **2c** as a control) were placed on a fresh PDA culture plate. Growth inhibition was analyzed after 10 days.

Separately, we have also determined that red light does not inhibit the growth of *Botrytis cinerea*, as the mycelium develops normally after irradiating the plate for 24 hours.

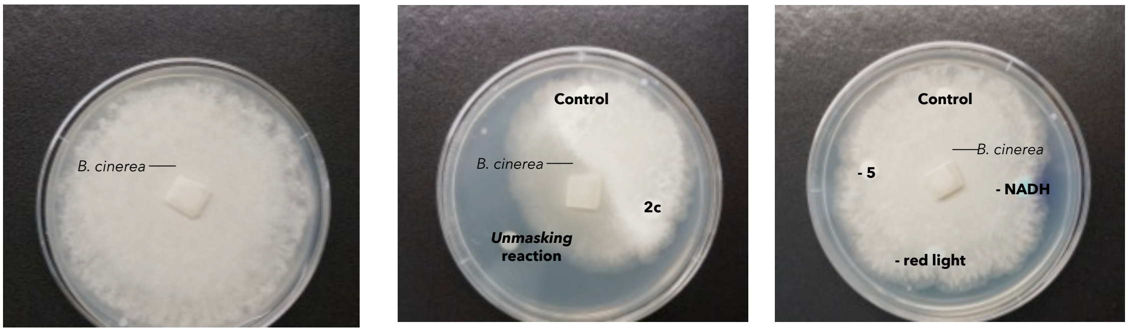

B. cinerea culture after 10 days

Boscalid-TT (right) vs *Unmasking* reaction (left)

Unmasking reaction (control experiments)

Figure S46. Growth inhibitory effect of boscalid–TT **2c** unmasking reaction in *Botrytis cinerea* after 10 days of culture. 10 mm mycelial plugs of 10-day culture of *B. cinerea* and discs containing crudes from unmasking and control reactions (without red light, without methylene blue **5** or without NADH) were placed on a fresh PDA culture plate. Discs containing DMSO or **2c** were also added as negative controls. Growth inhibition was analyzed after 10 days.

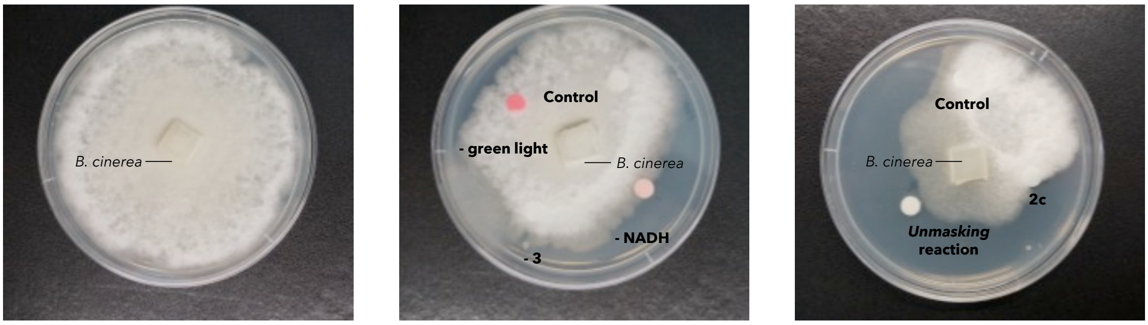

B. cinerea culture after 10 days

Boscalid-TT (right) vs *Unmasking* reaction (left)

Unmasking reaction (control experiments)

Figure S47. Growth inhibitory effect of boscalid–TT **2c** unmasking reaction in *Botrytis cinerea* after 10 days of culture. 10 mm mycelial plugs of 10-day culture of *B. cinerea* and discs containing crudes from unmasking and control reactions (without green light, without Eosin Y **3** or without NADH) were placed on a fresh PDA culture plate. Discs containing DMSO or **2c** were also added as negative controls. Growth inhibition was analyzed after 10 days.

The successful inhibition displayed by the uncaging reactions (both with the red-light and green-light based systems) confirms the success of the uncaging process, followed using a biological process. Importantly, control experiments without light, photocatalyst or reductant show almost no inhibition of fungal growth. This highlights that our photoredox uncaging conditions are fully compatible with this living organism, and that the release of uncaged boscalid is the only factor resulting in growth inhibition.

Exposure of *B. cinerea* to pyriproxyfen-TT (2a) and anisole-TT (2e) unmasking reaction mixtures

To fully verify that other components released or generated during the uncaging reaction of boscalid-TT (such as thianthrene) are not responsible for the observed toxicity in *Botrytis cinerea*, disc diffusion experiments were also performed with two additional protected *dummy* molecules (pyriproxyfen-TT **2a** and anisole-TT **2e**), in which the unprotected counterparts were not expected to exhibit any antifungal activity.

Disc diffusion assays were carried out as previously described. Briefly, blank antimicrobial paper discs embedded with 20 µL (at 10 mM concentration) of **2a** and **2e** unmasking reaction mixtures were added to Potato Dextrose Agar (PDA, Sigma-Aldrich) plates. Discs containing 20 µL of 10 mM boscalid-TT **2c** reaction mixture or 20 µL of DMSO were also added as positive and negative controls, respectively.

Once discs were air-dried, 10 mm mycelial plugs from 10-day-old *B. cinerea* cultures were placed in the center of each PDA plate, and embedded discs were placed around it. Plates were then incubated at 20 ºC, under light, and growth inhibition was evaluated after 10 days of culture.

Results obtained with each of the unmasking reactions

Release of bioactive 1c (boscalid)

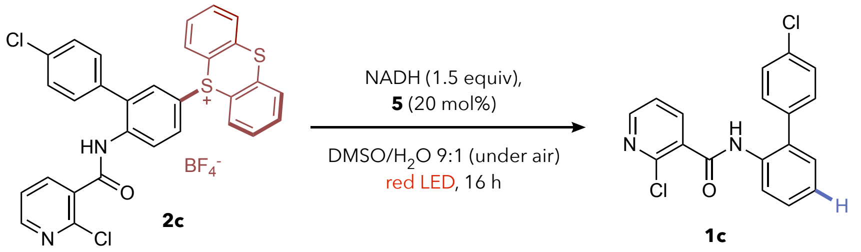

Release of inactive compounds 1a and 1e (dummy experiments)

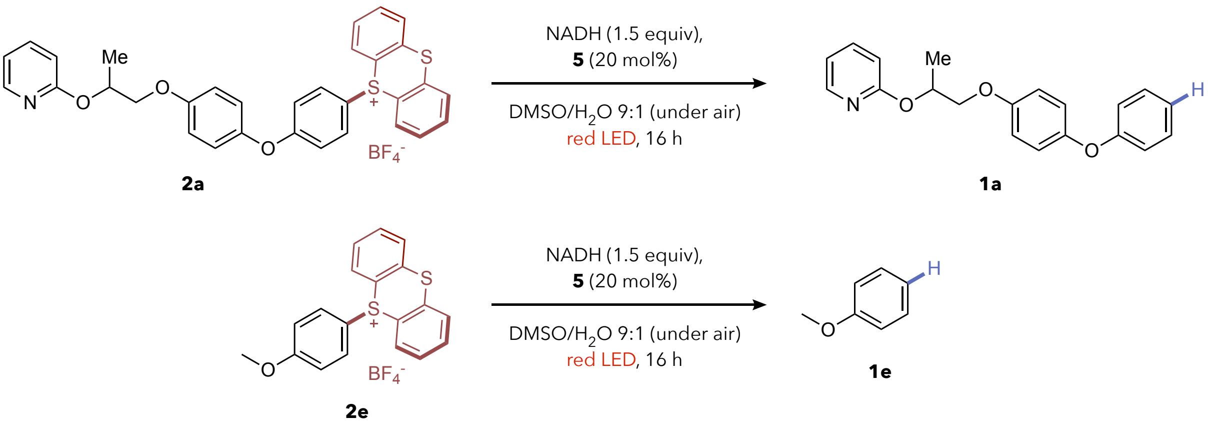

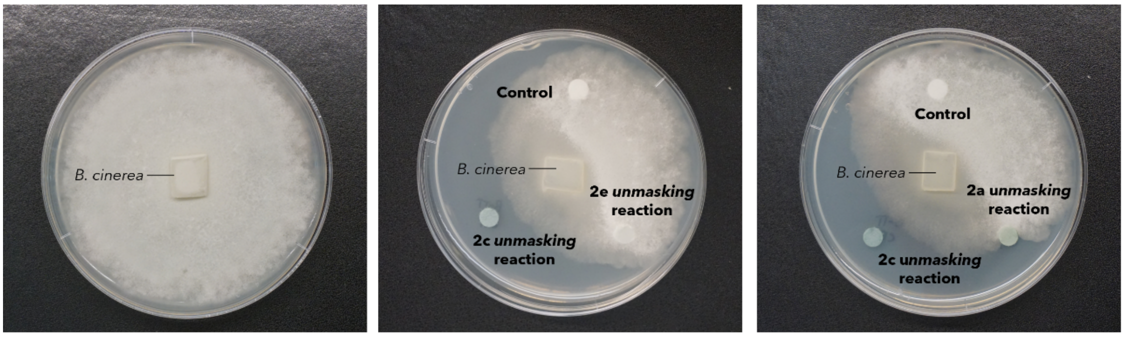

B. cinerea culture after 10 days | Boscalid-TT (**2c**) *unmasking* reaction (left) vs **2e** *unmasking* reaction (right) | Boscalid-TT (**2c**) *unmasking* reaction (left) vs **2a** *unmasking* reaction (right)

Figure S48. Growth inhibitory effect of boscalid–TT **2c** unmasking reaction in *Botrytis cinerea* after 10 days of culture. 10 mm mycelial plugs of 10-day culture of *B. cinerea* and discs containing crudes form **2a** and **2e** unmasking reactions were placed on fresh PDA culture plates. Discs containing DMSO or crudes from **2c** unmasking reaction were also added as negative and positive controls, respectively. Growth inhibition was assessed after 10 days.

As expected, none of the additionally tested unmasking reactions (with **2a** to release pyriproxyfen or with **2e** to release anisole) showed growth-inhibitory effects in *Botrytis cinerea*, whereas a clear growth inhibition was again observed in the boscalid–TT **2c** unmasking reaction, suggesting that thianthrene release is not responsible of the observed antifungal effects.

14. NMR spectra

Pyriproxyfen derived thianthrenium salt (2a)

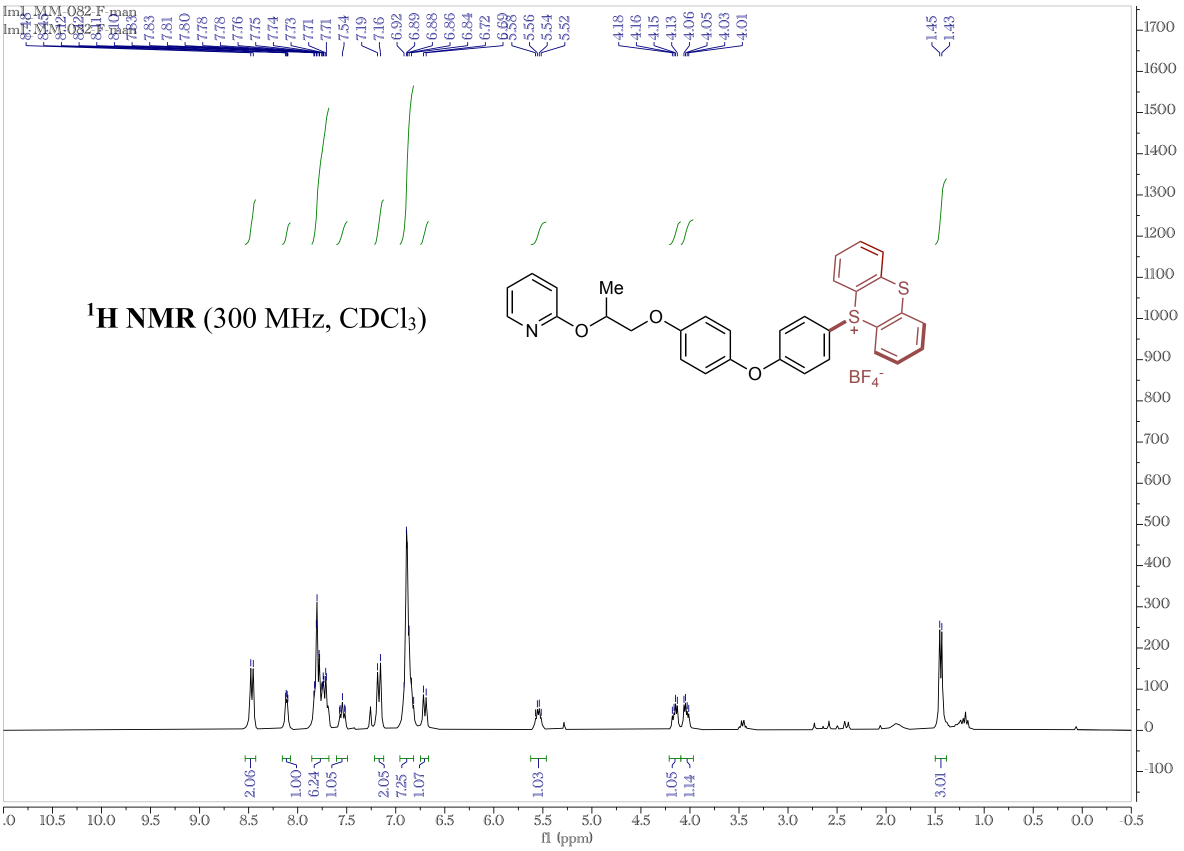

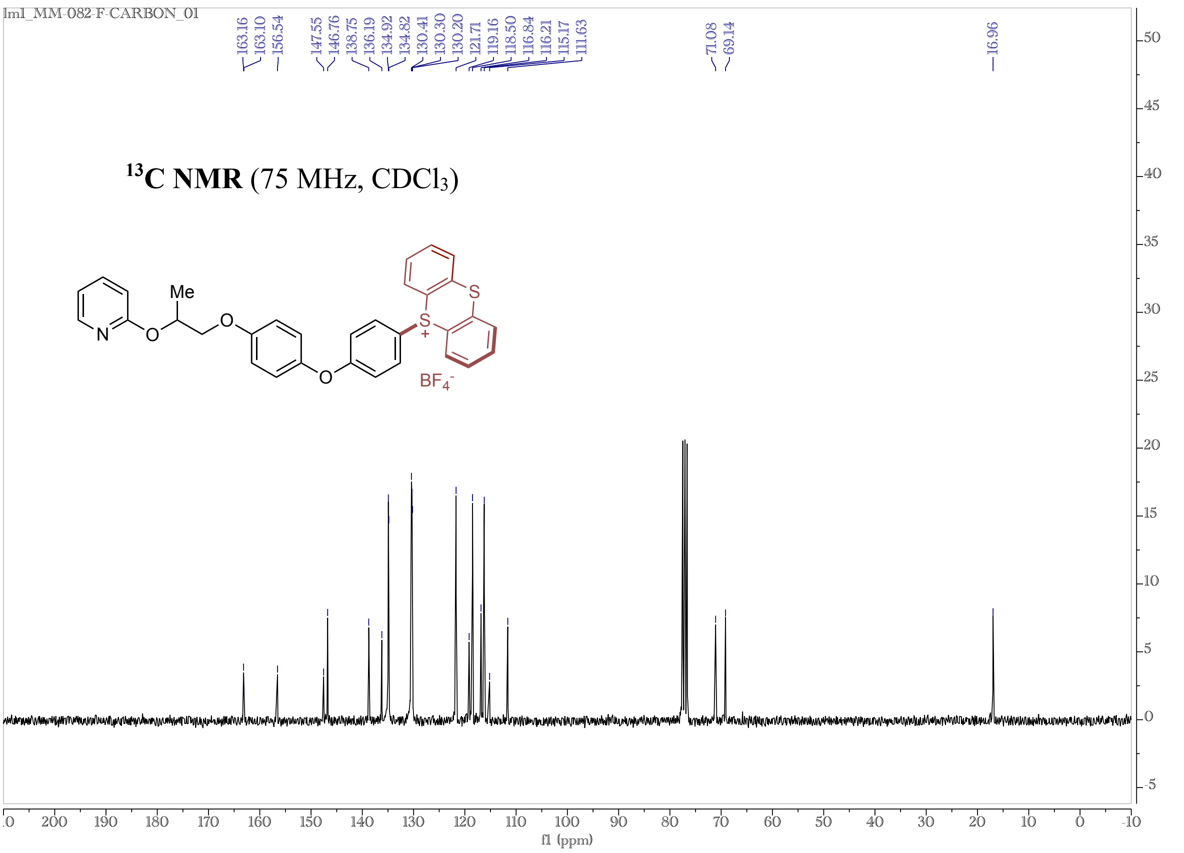

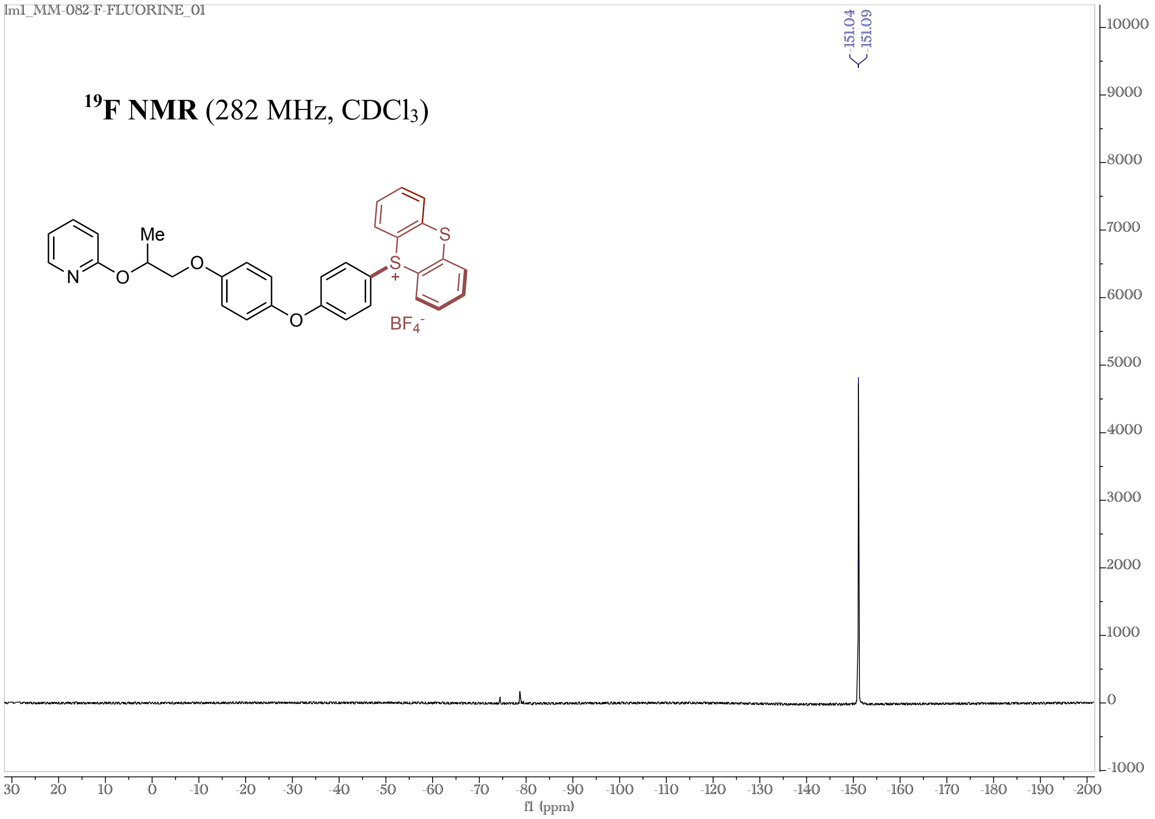

Unmasked pyriproxyfen (1a) ^{1}H NMR

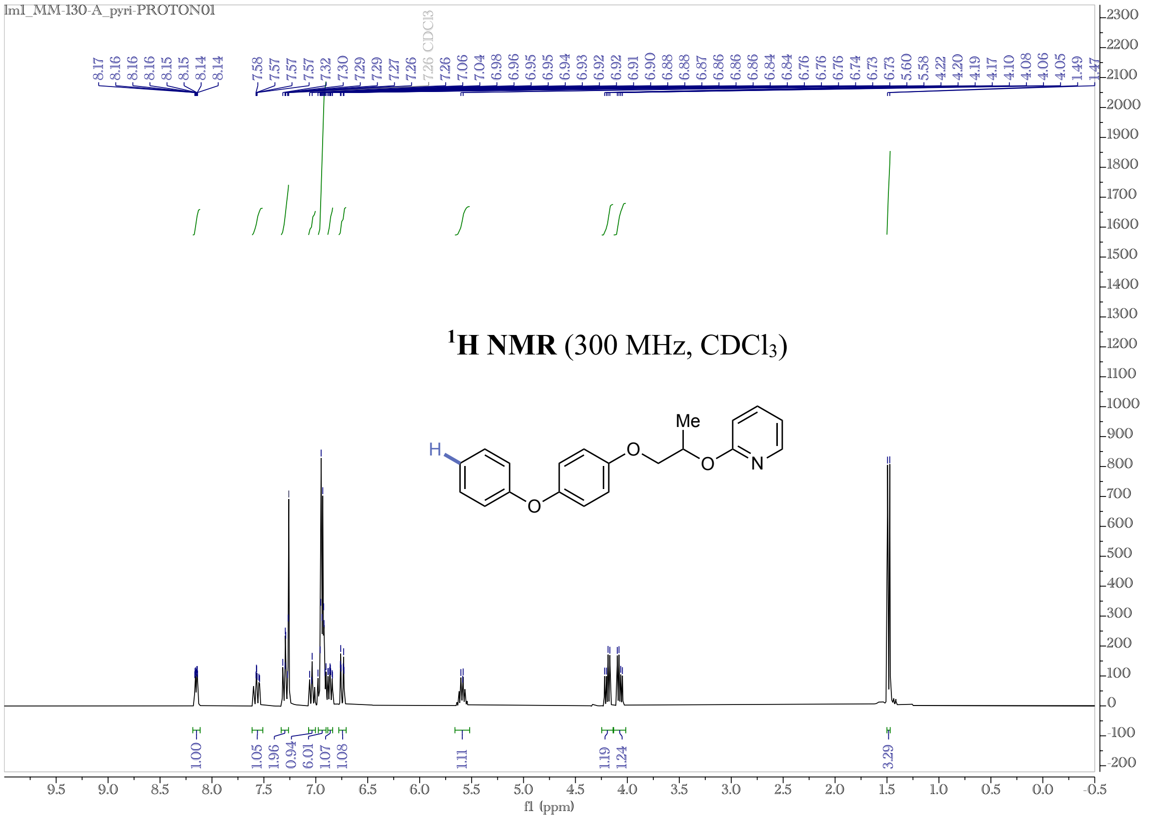

Napropamide derived thianthrenium salt (2b)

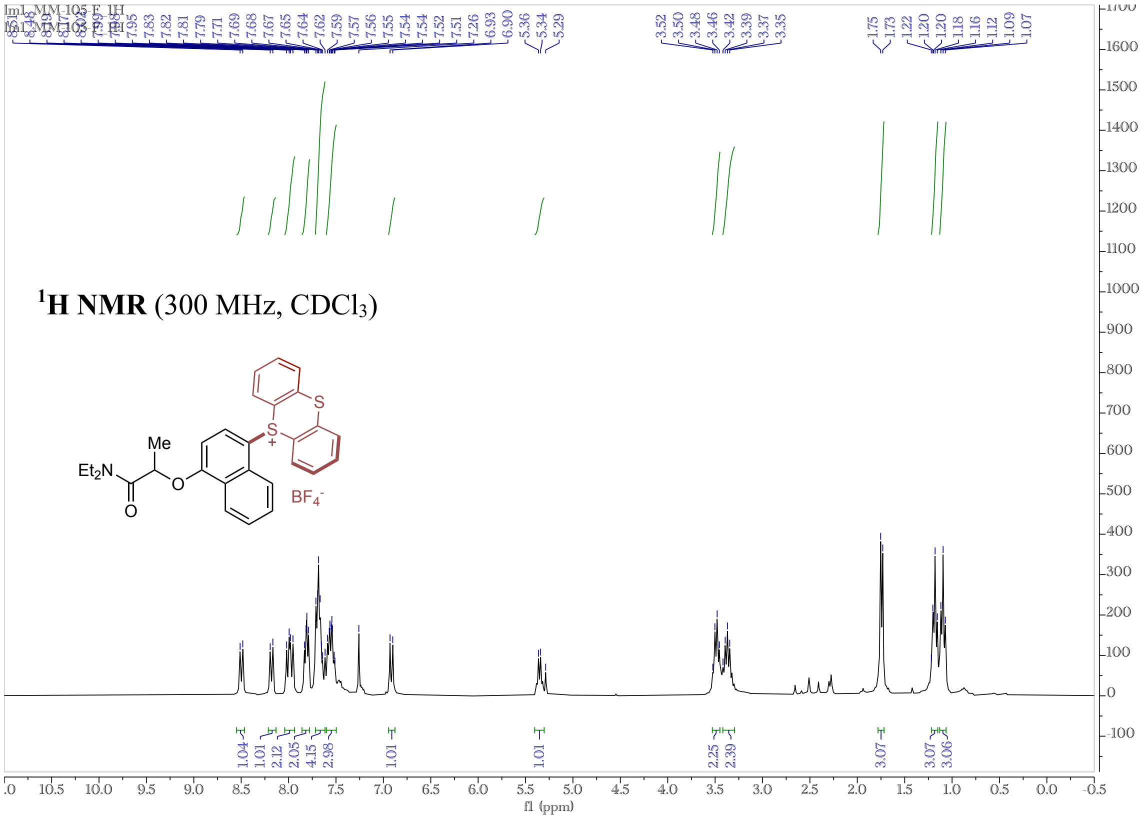

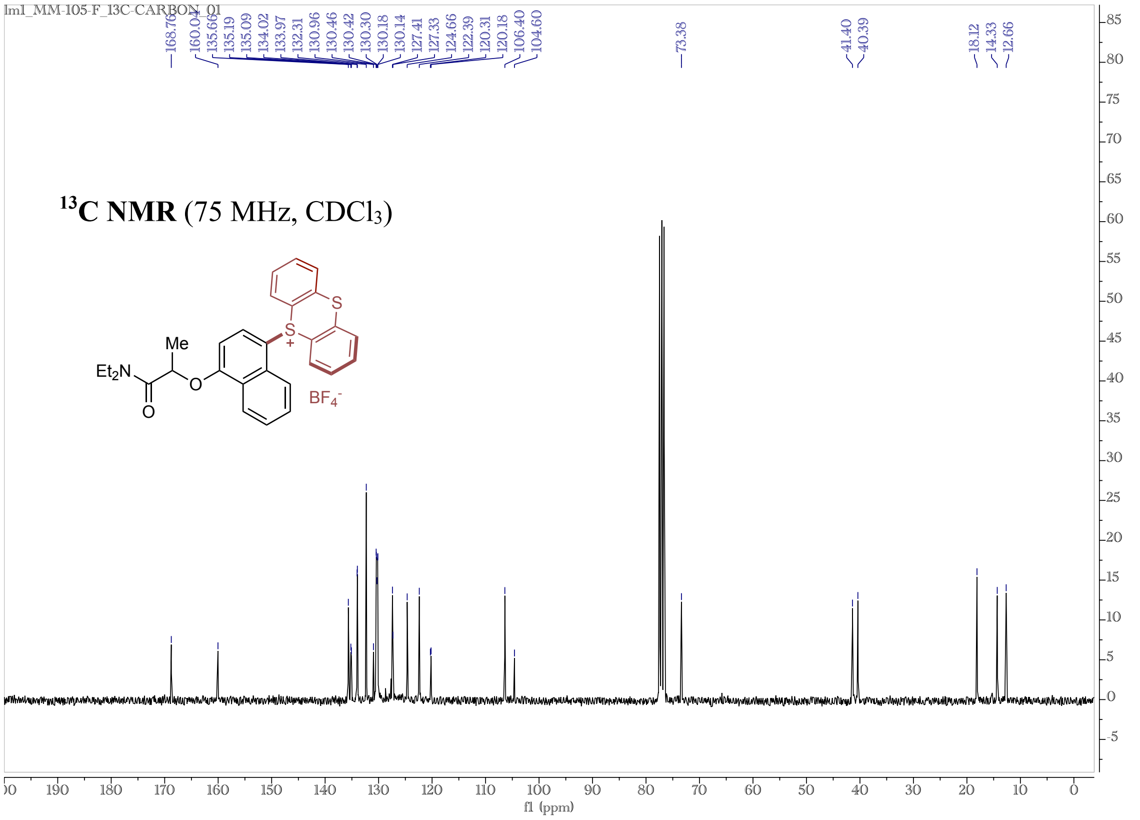

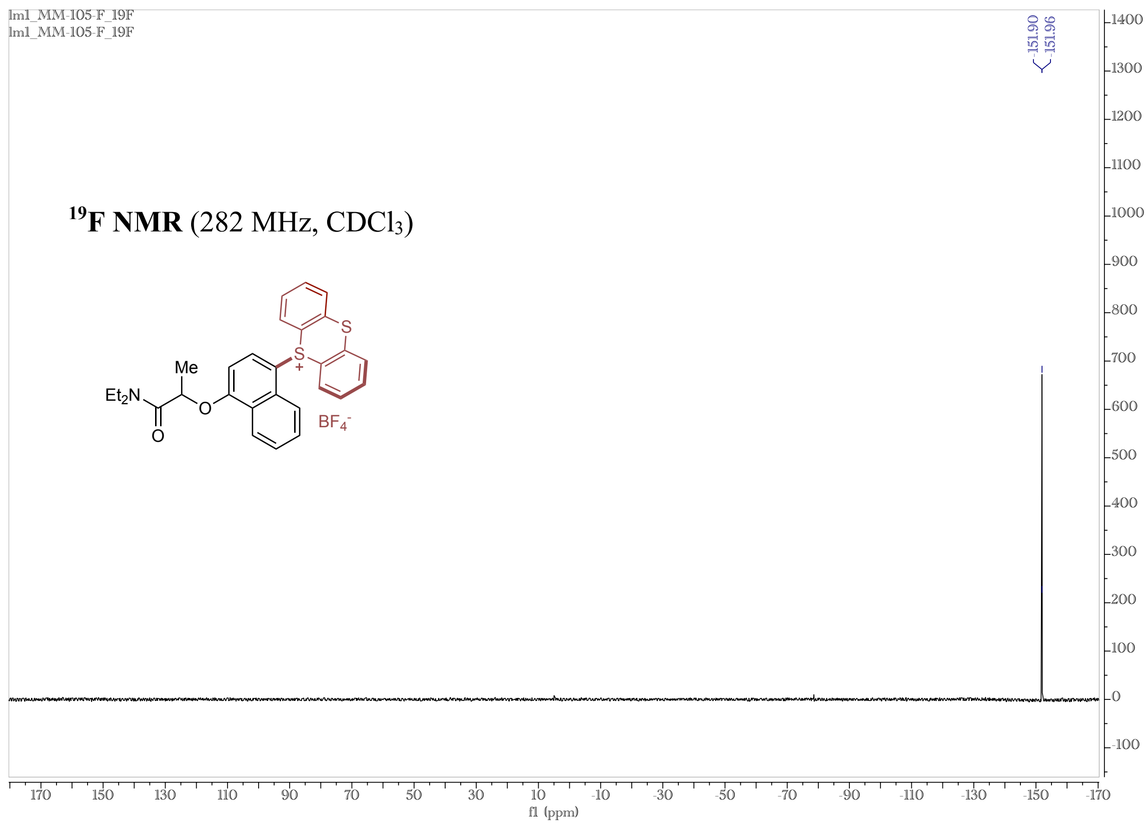

Unmasked napropamide (1b) ^{1}H NMR

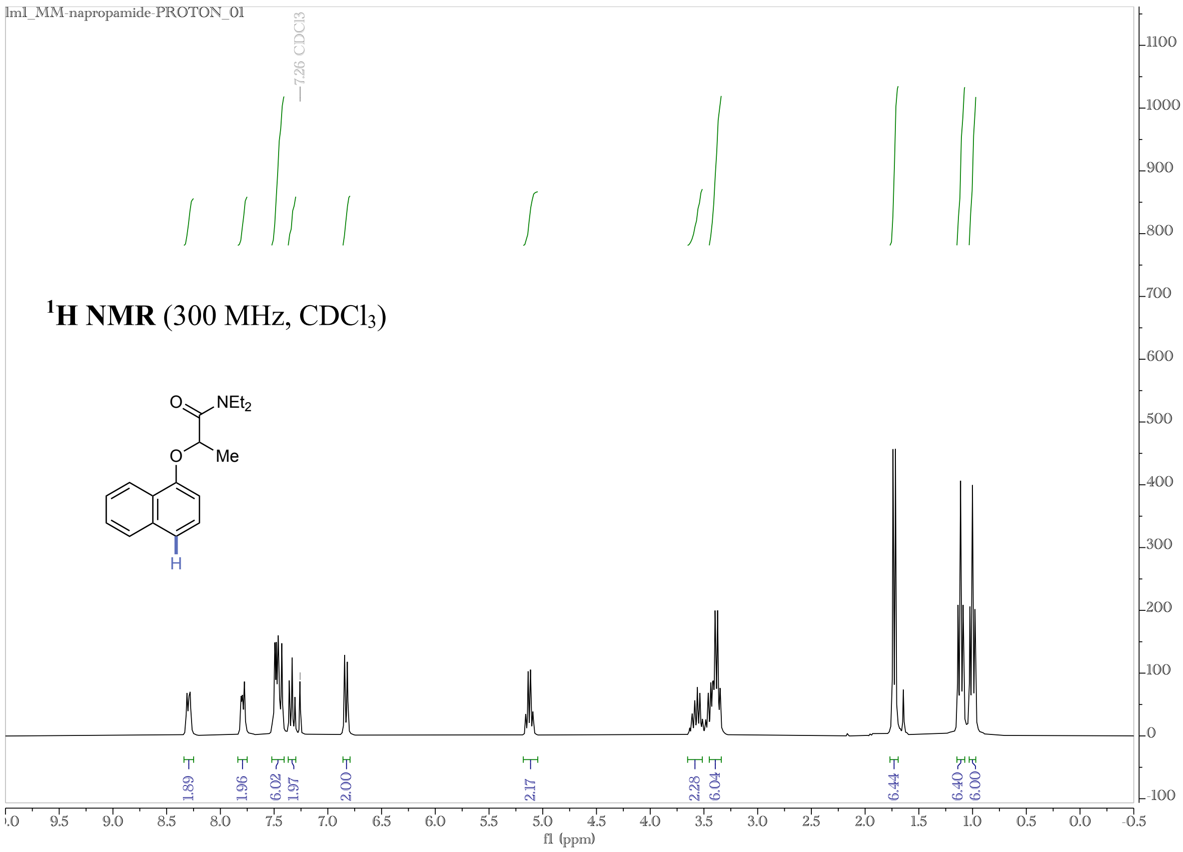

Boscalid derived thianthrenium salt (2c)

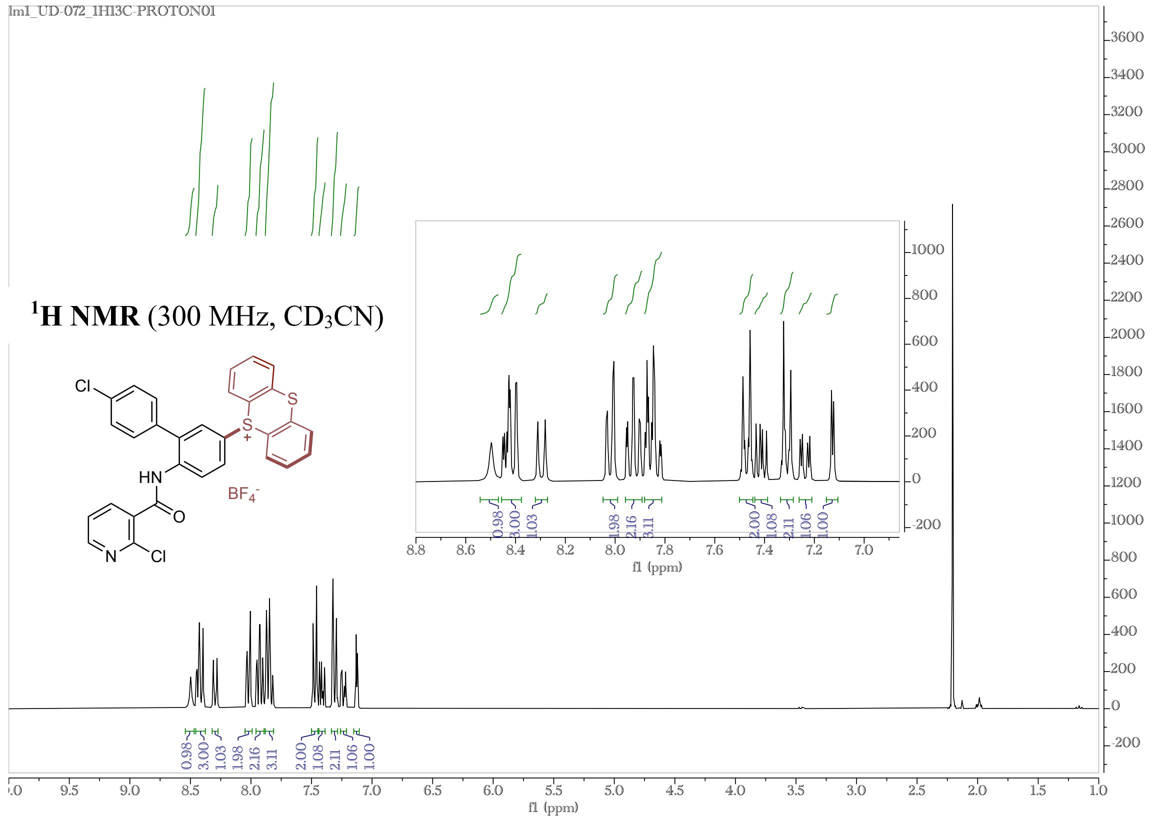

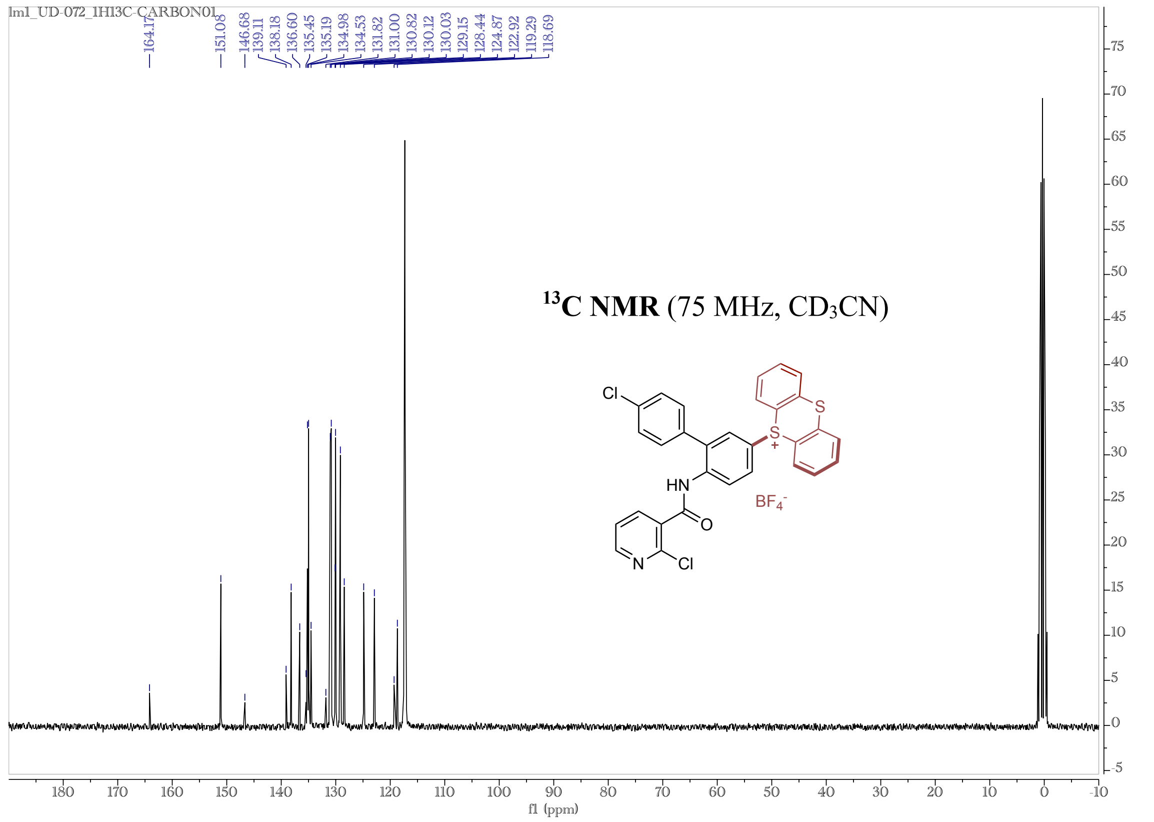

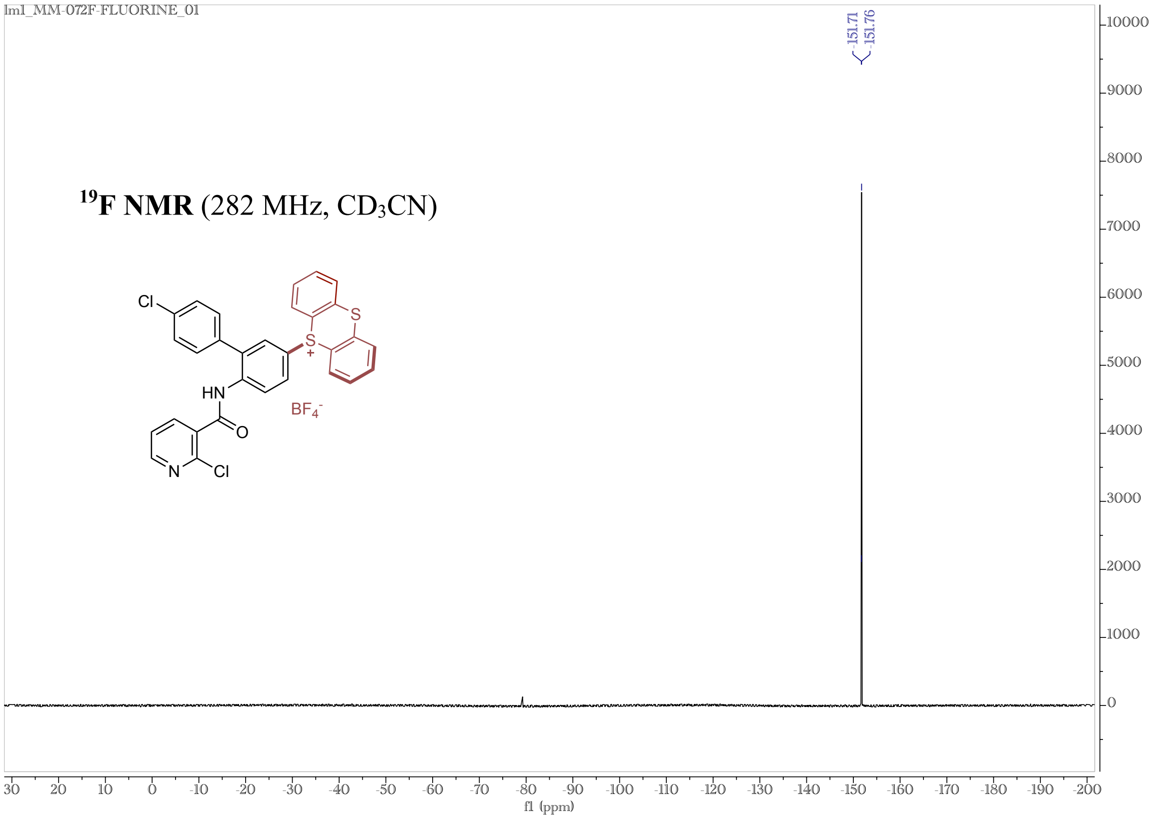

Unmasked boscalid (1c) ^{1}H NMR

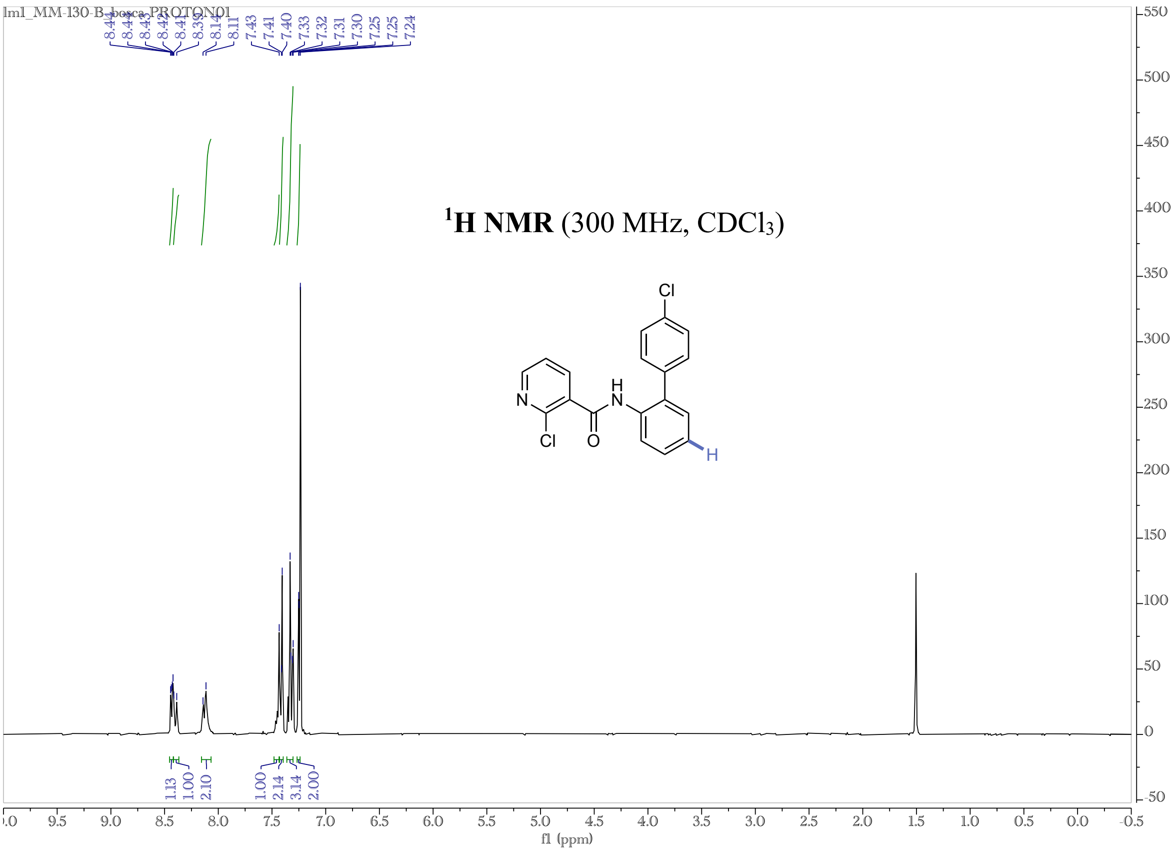

GW-9662 derived thianthrenium salt (2d)

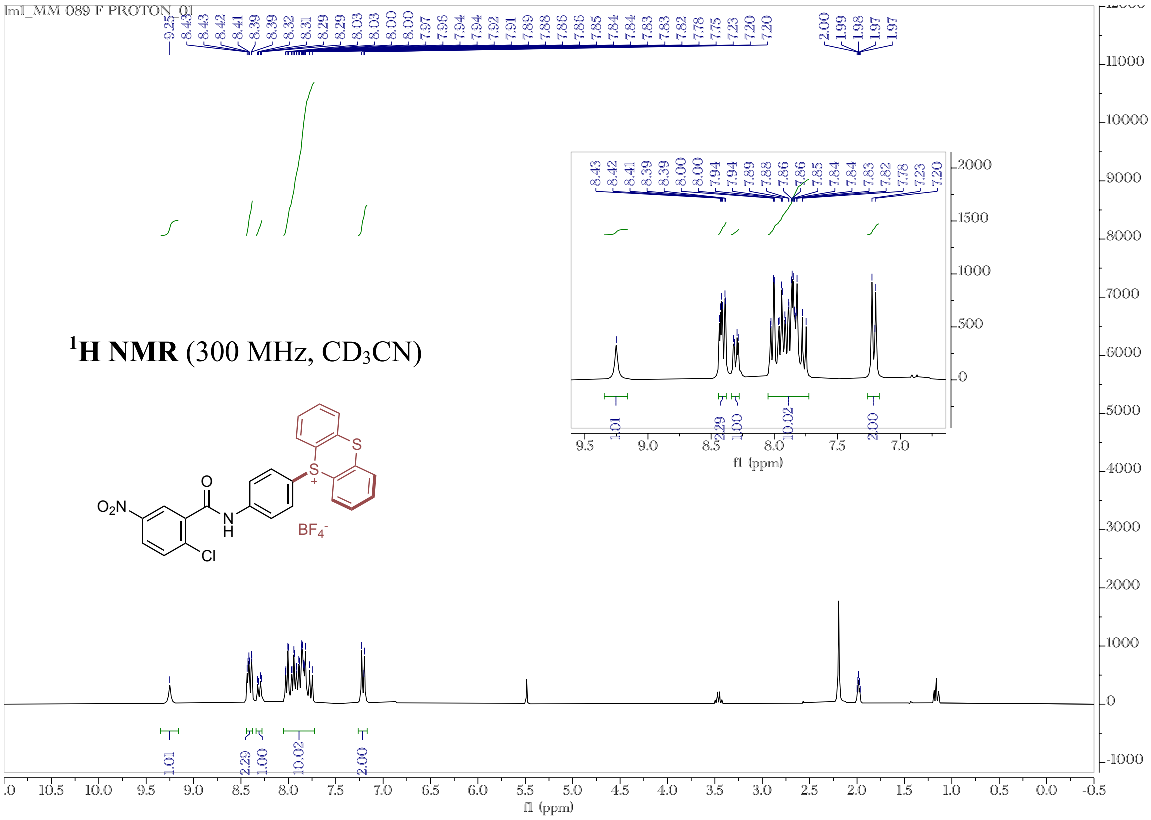

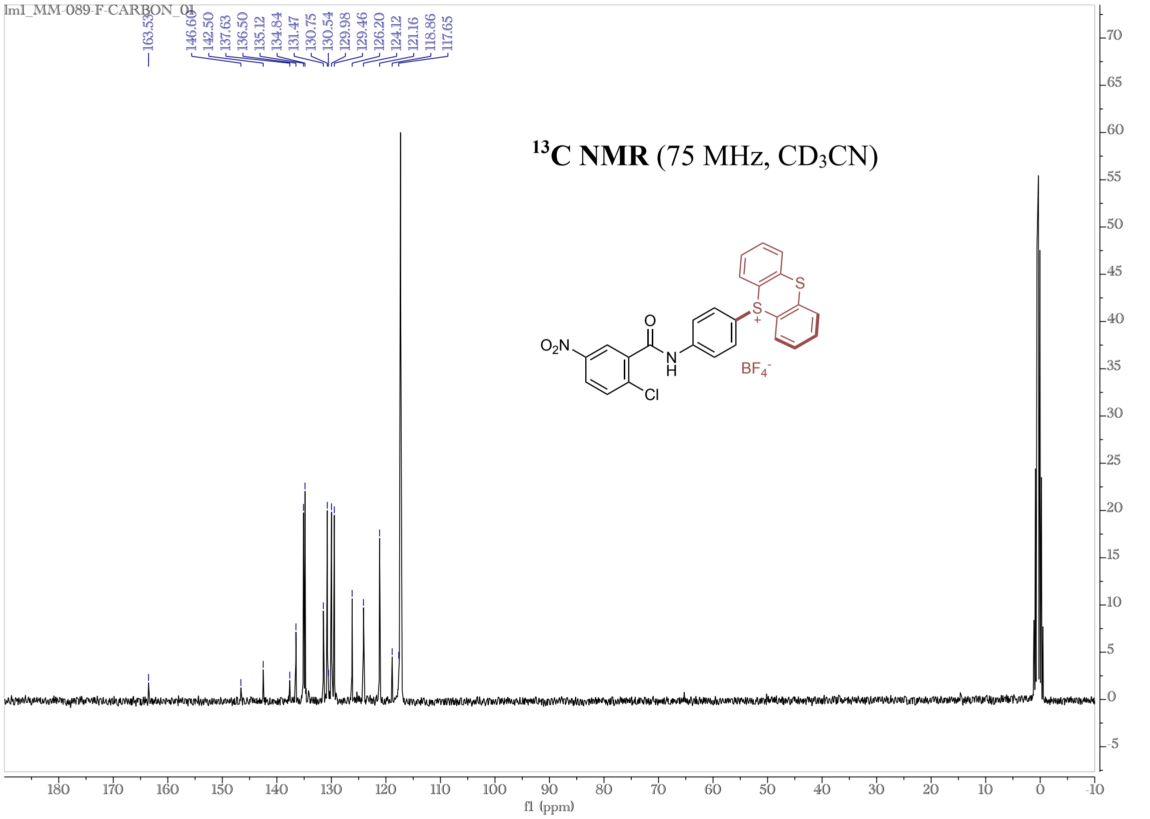

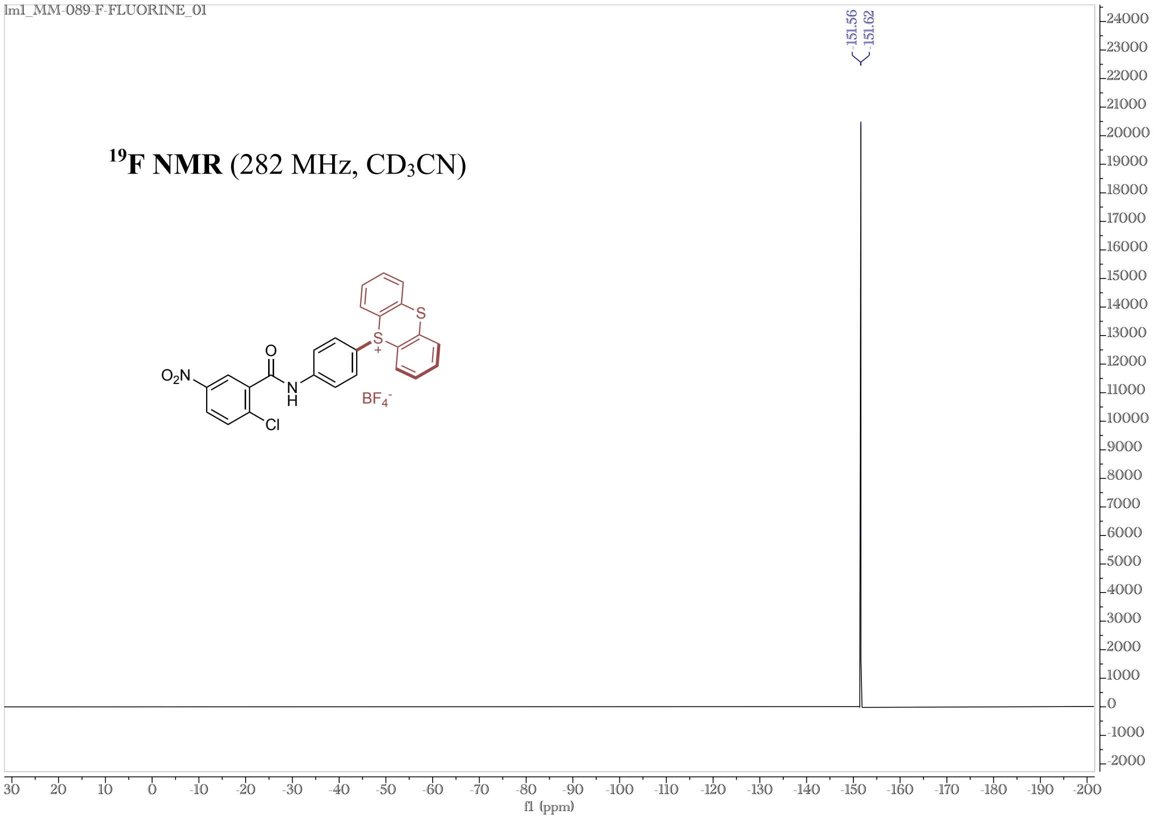

Unmasked GW-9662 (1d) ^{1}H NMR

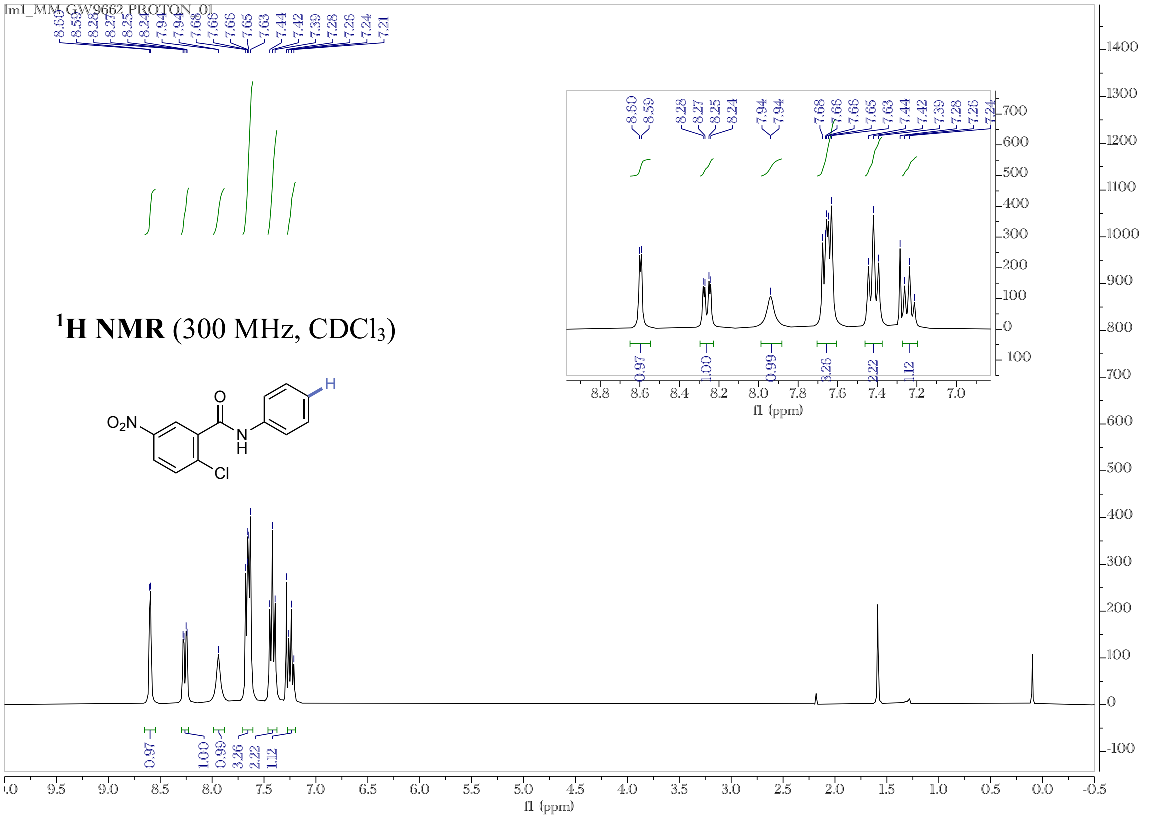

Anisole derived thianthrenium salt (2e)

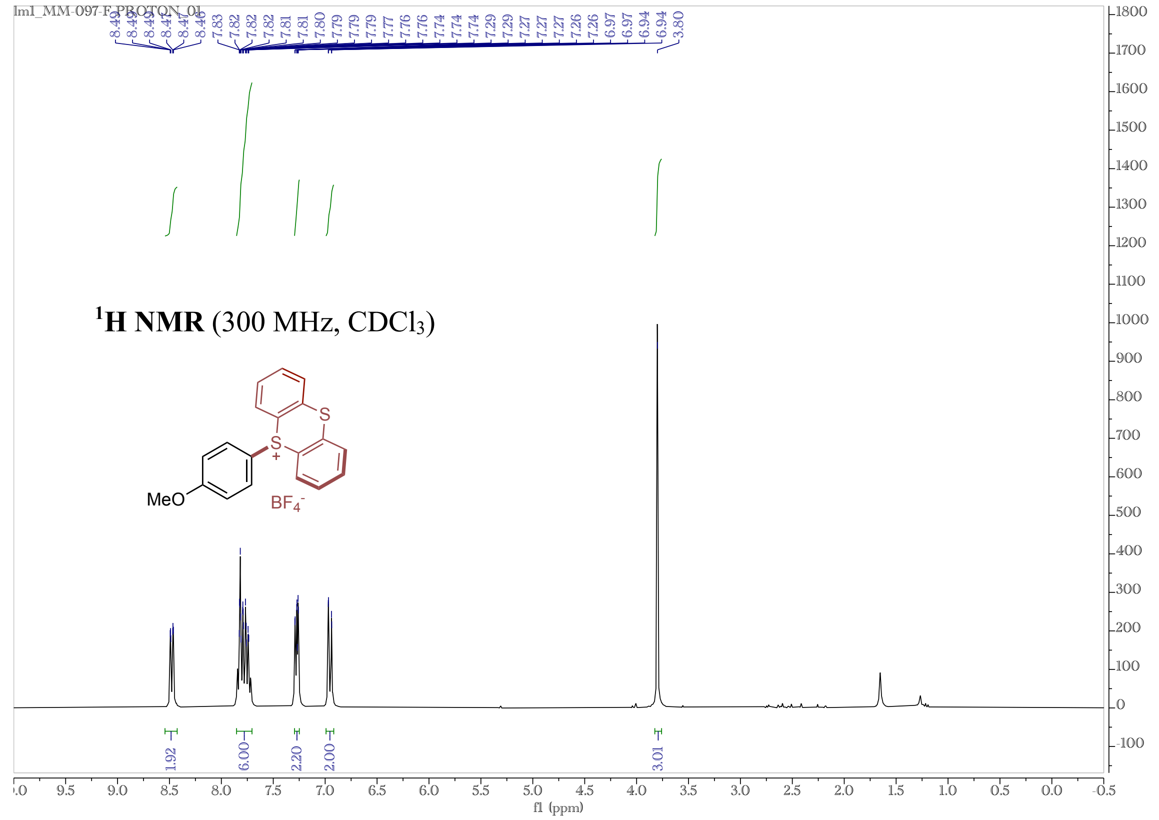

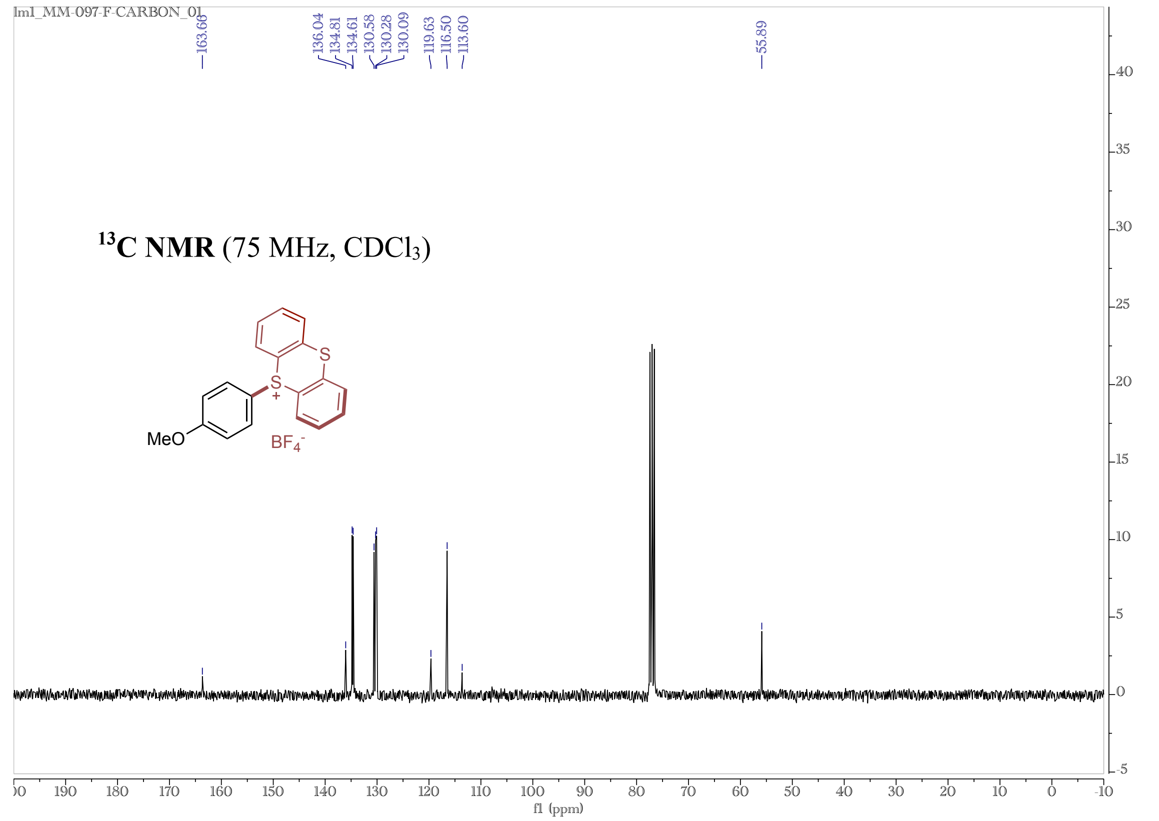

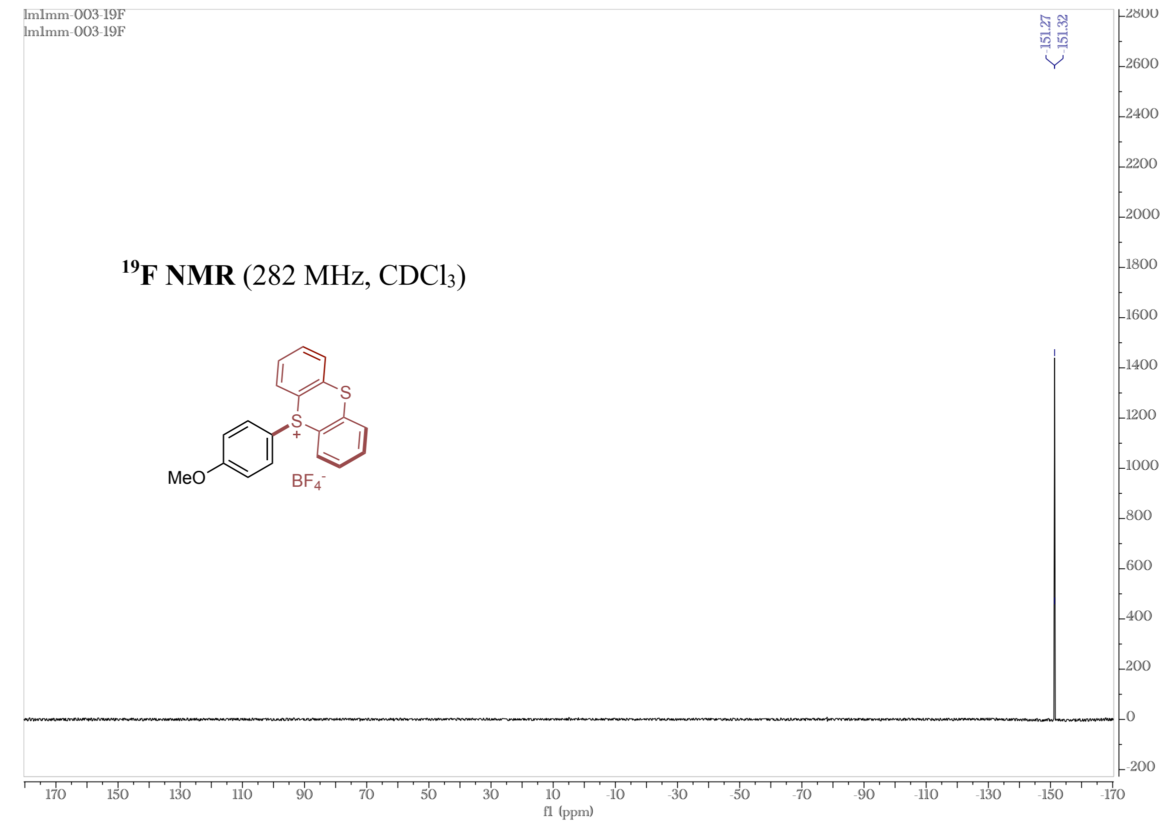

Unmasked anisole (1e) ^{1}H NMR

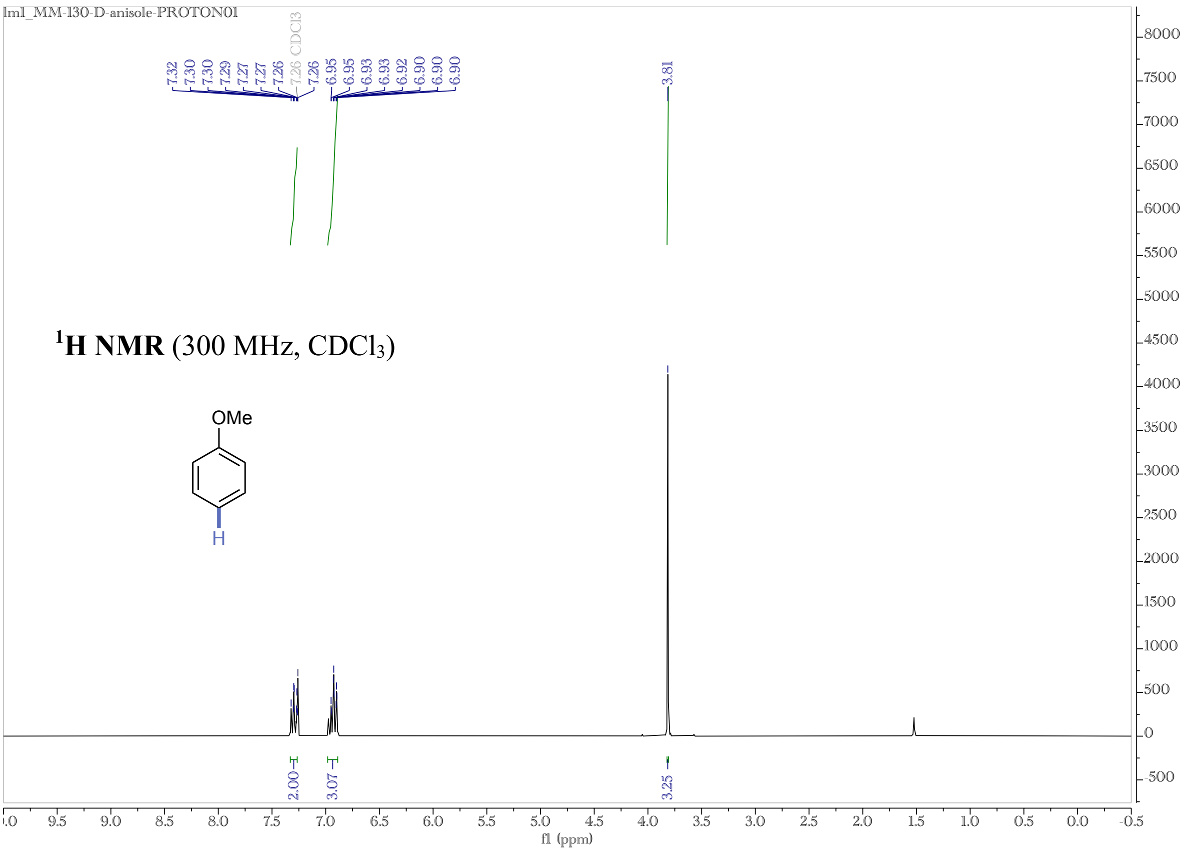

1-Bromo-4-phenoxybenzene derived thianthrenium salt (2f)

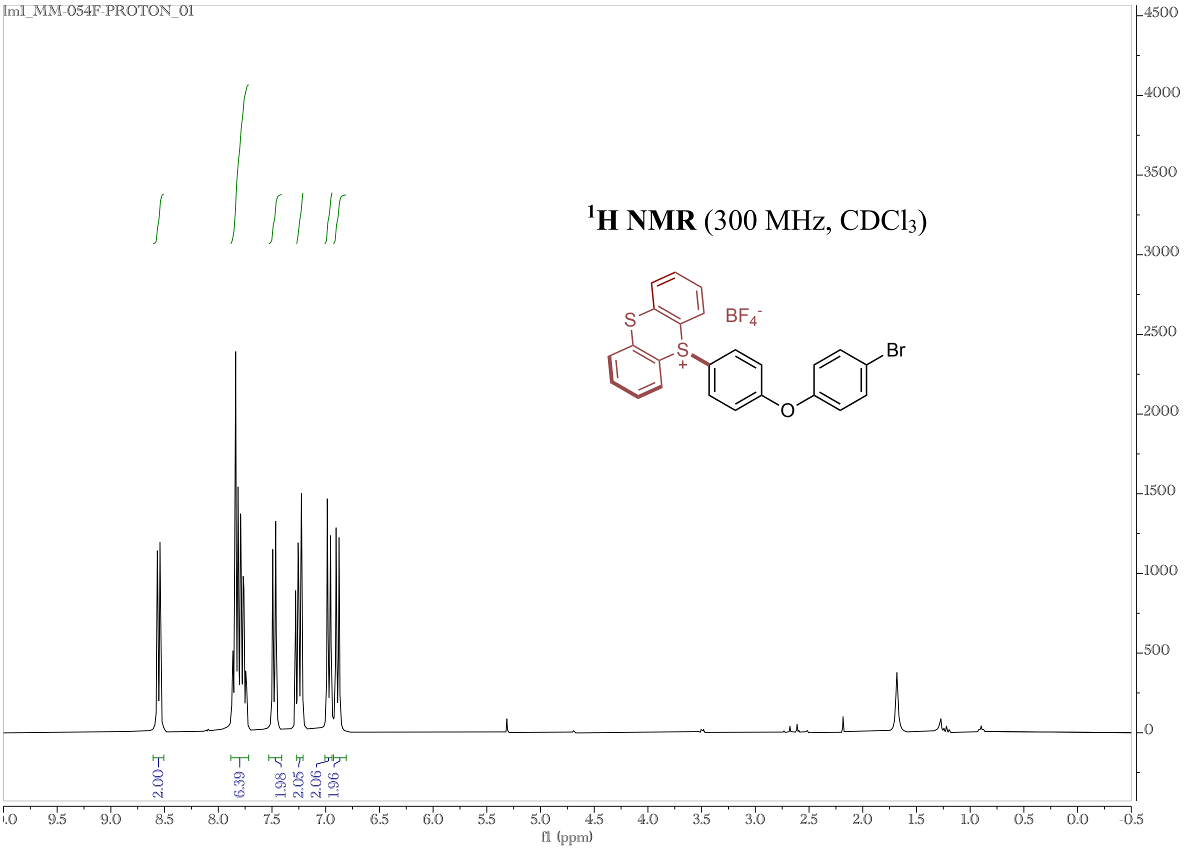

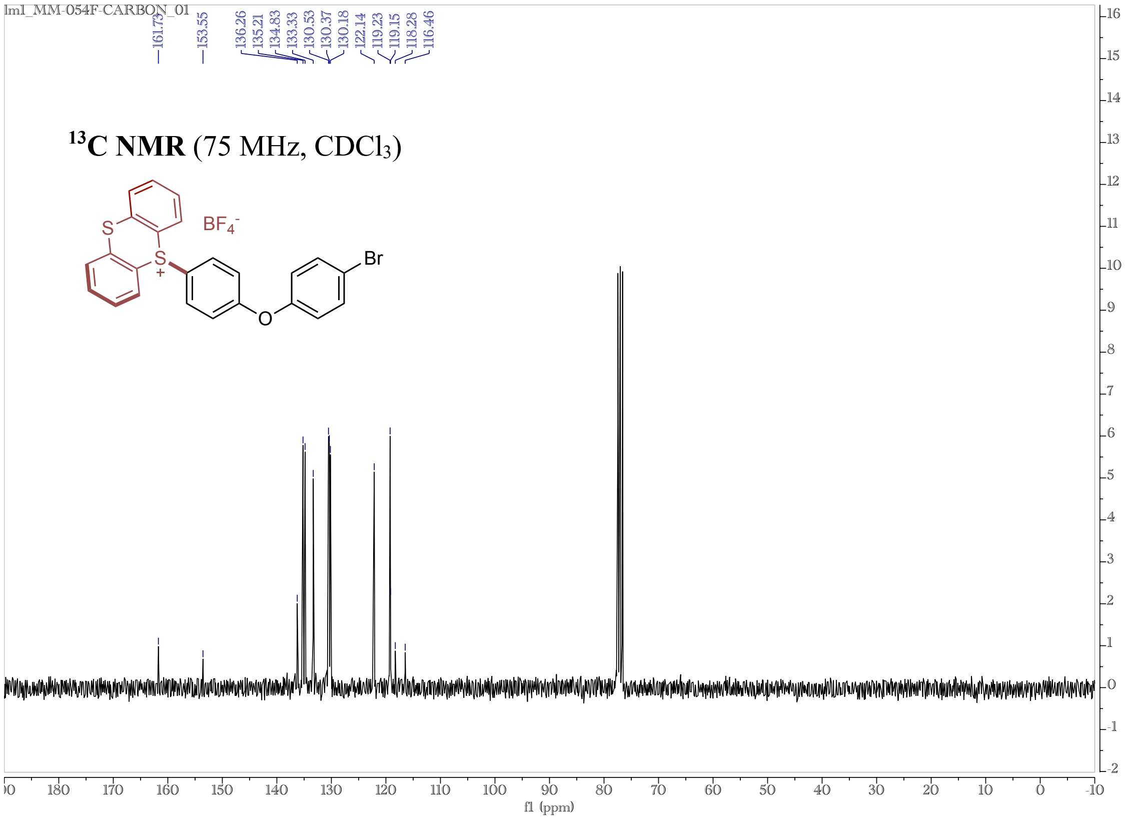

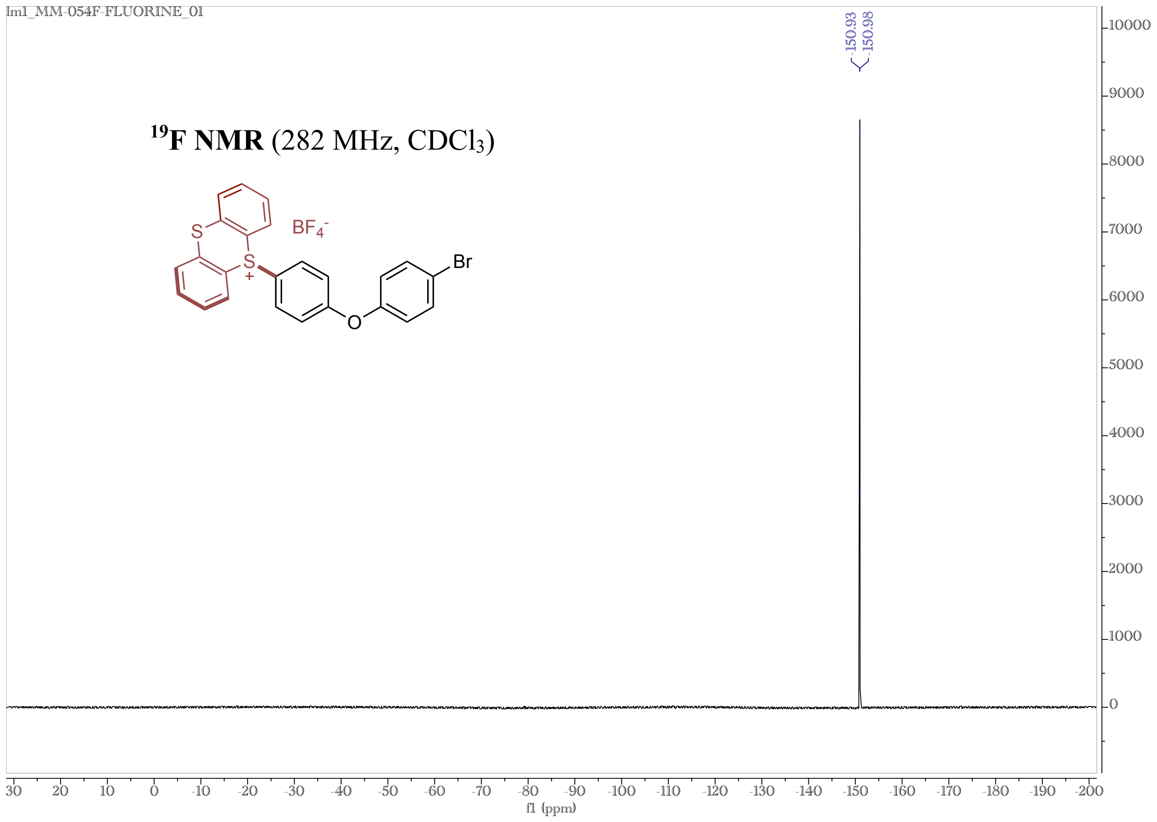

Unmasked 1-bromo-4-phenoxybenzene (1f) ^{1}H NMR

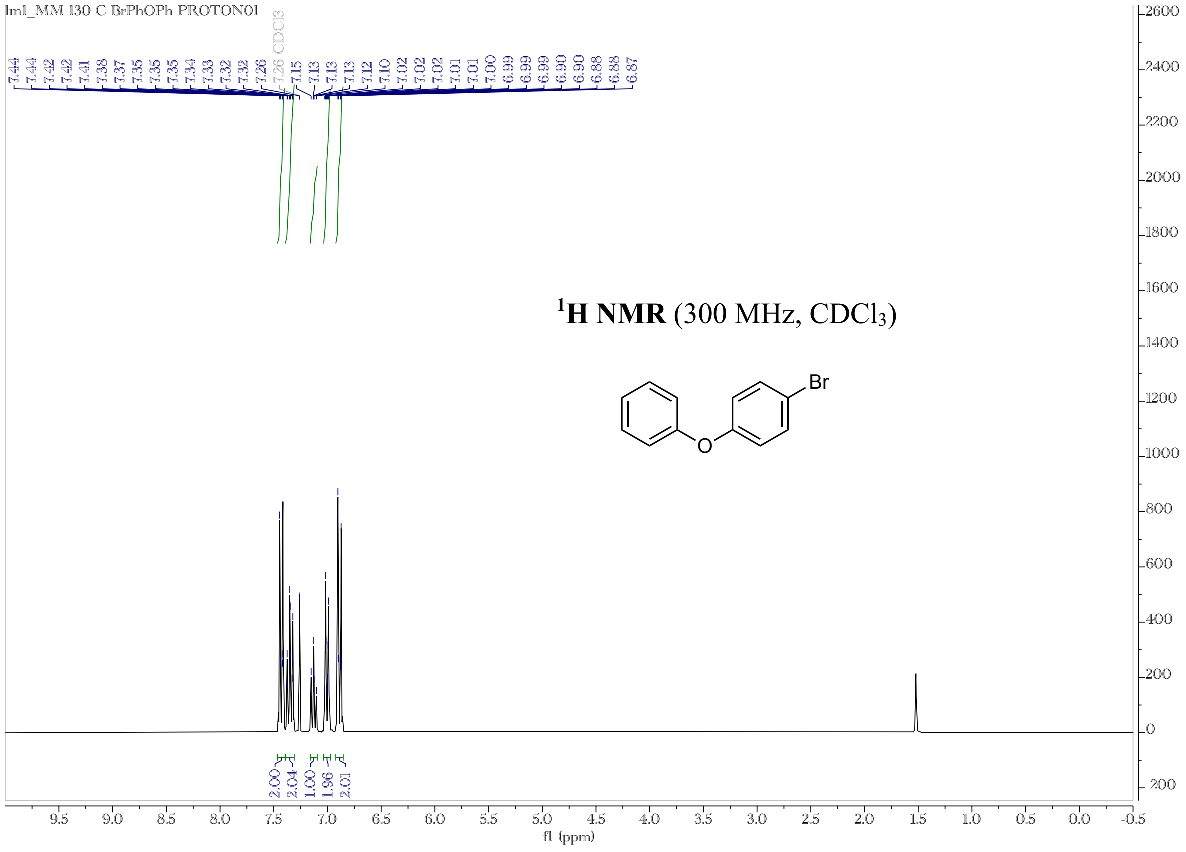

1,1,2,2-Tetraphenylethene derived thianthrenium salt [TPE–TT]OTf (**2g**)

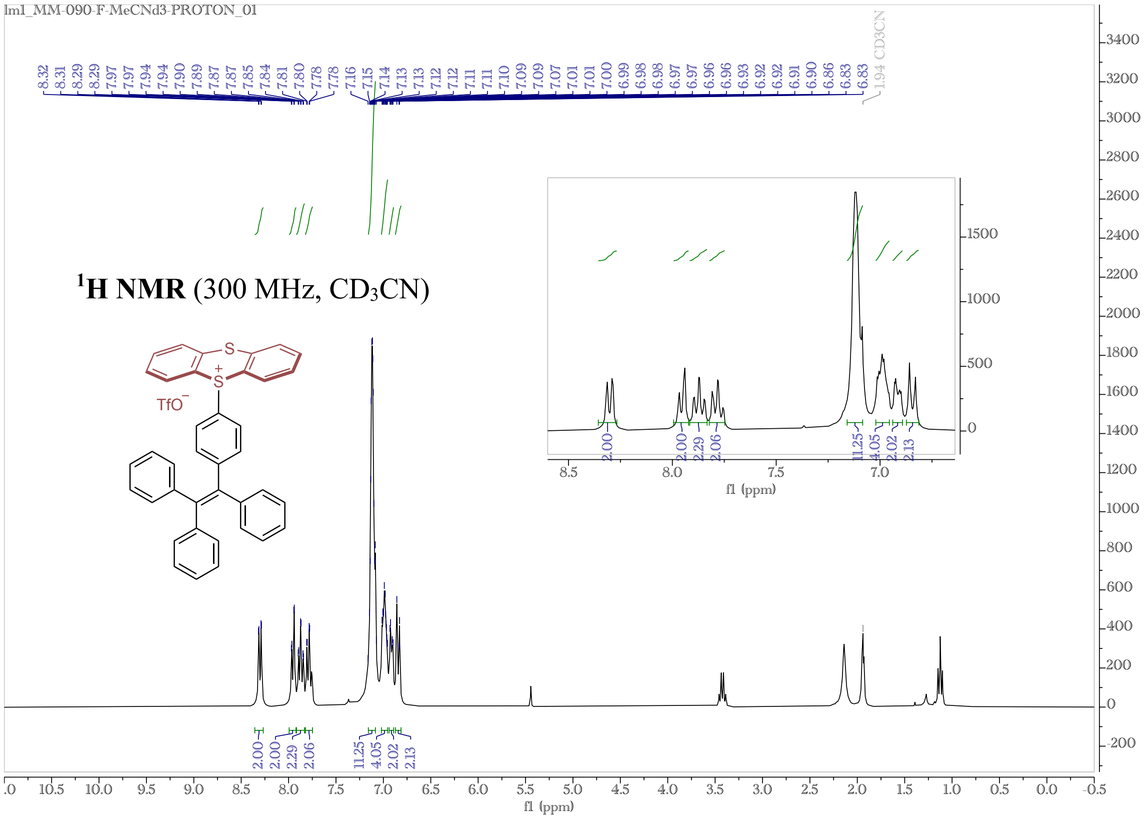

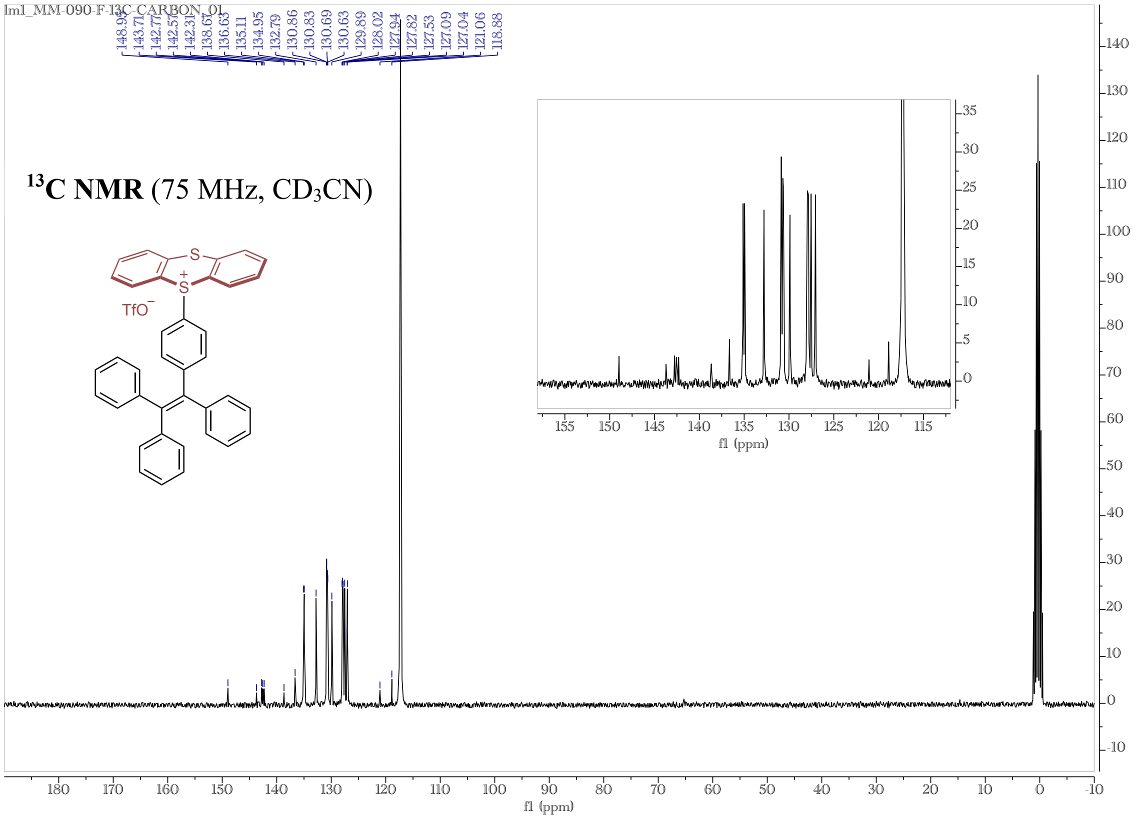

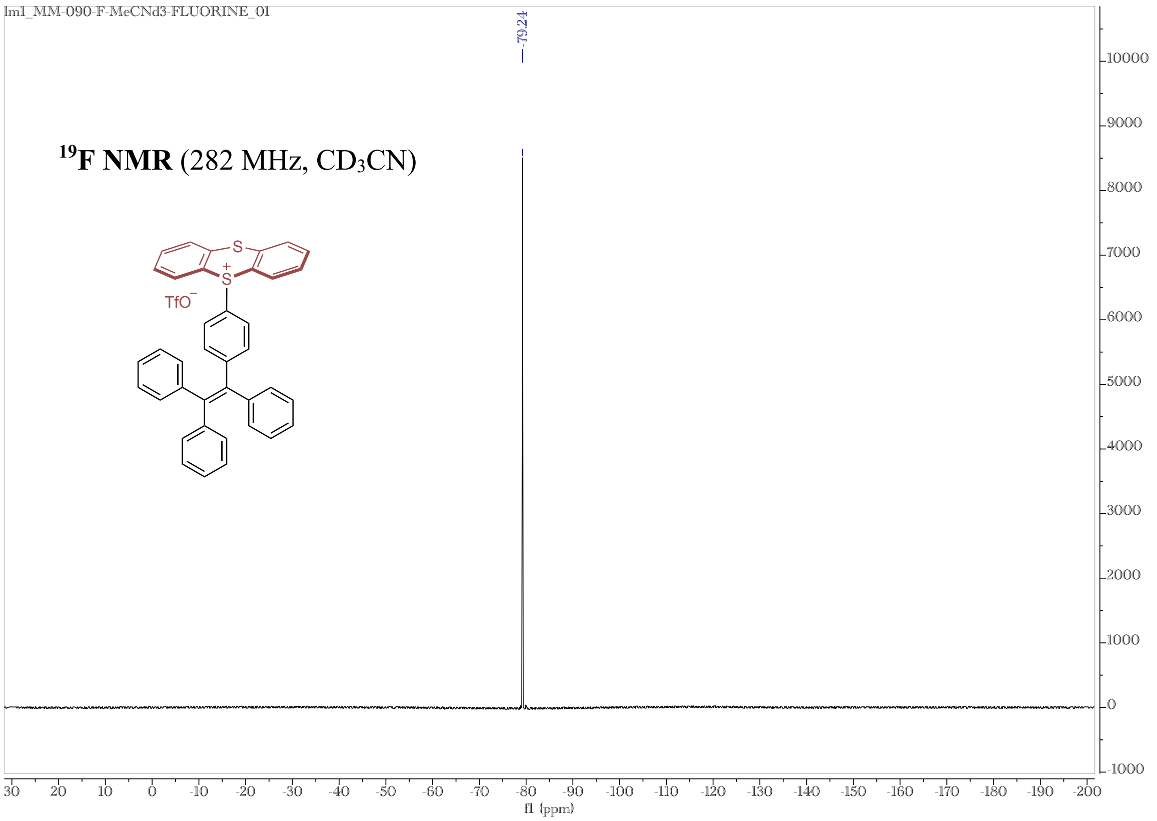

Unmasked 1,1,2,2-tetraphenylethylene (1g) ^{1}H NMR

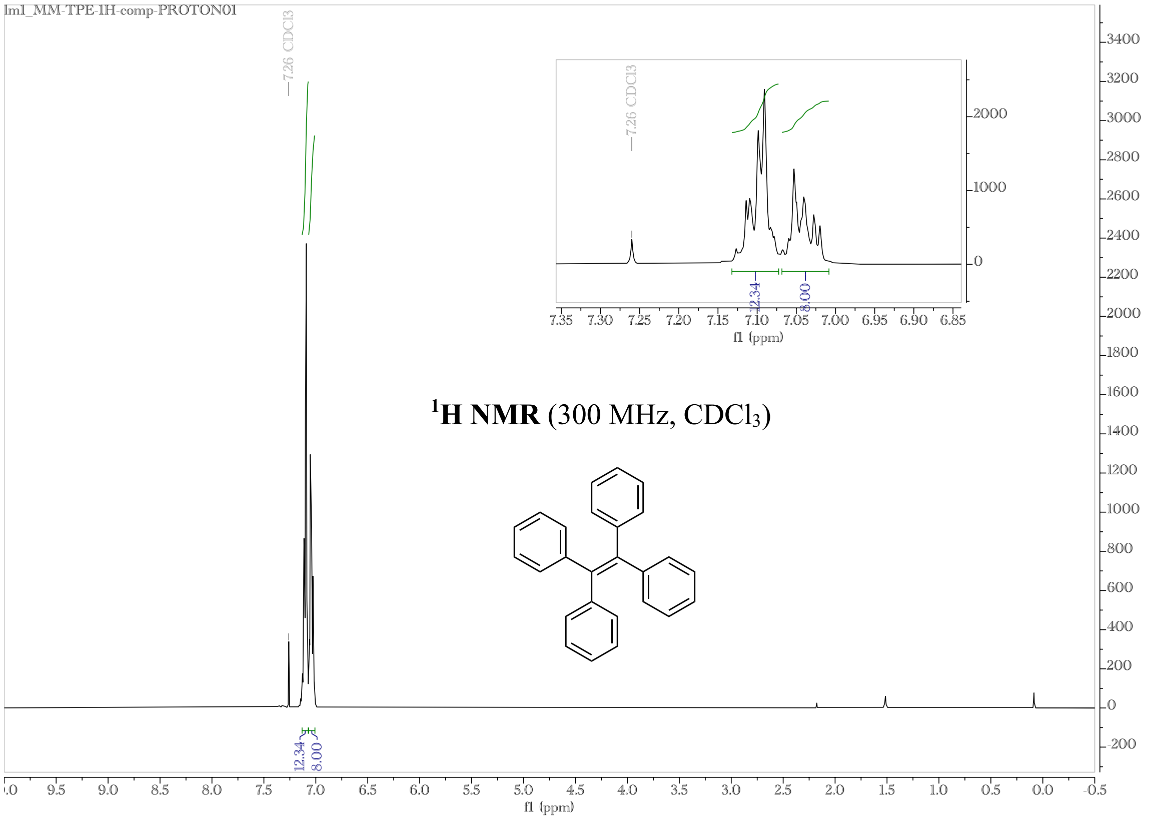

Lidocaine derived thianthrenium salt (SI-1)

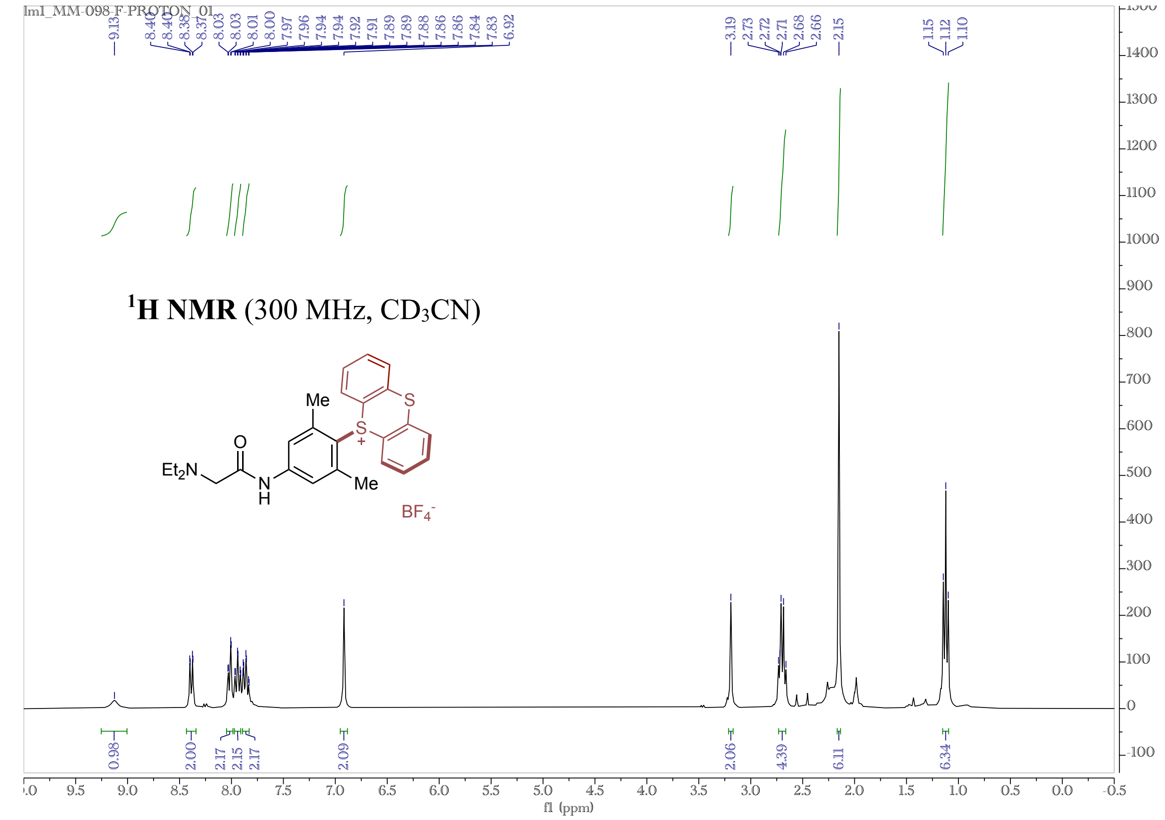

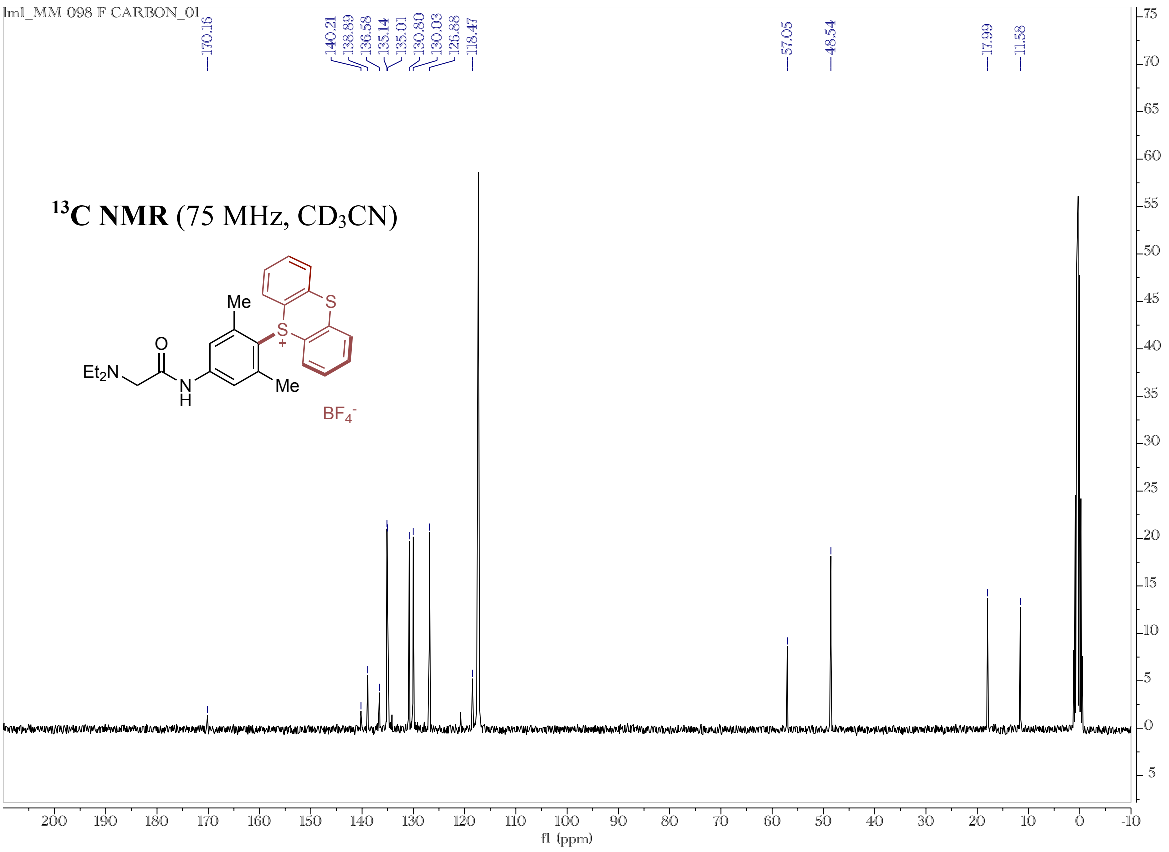

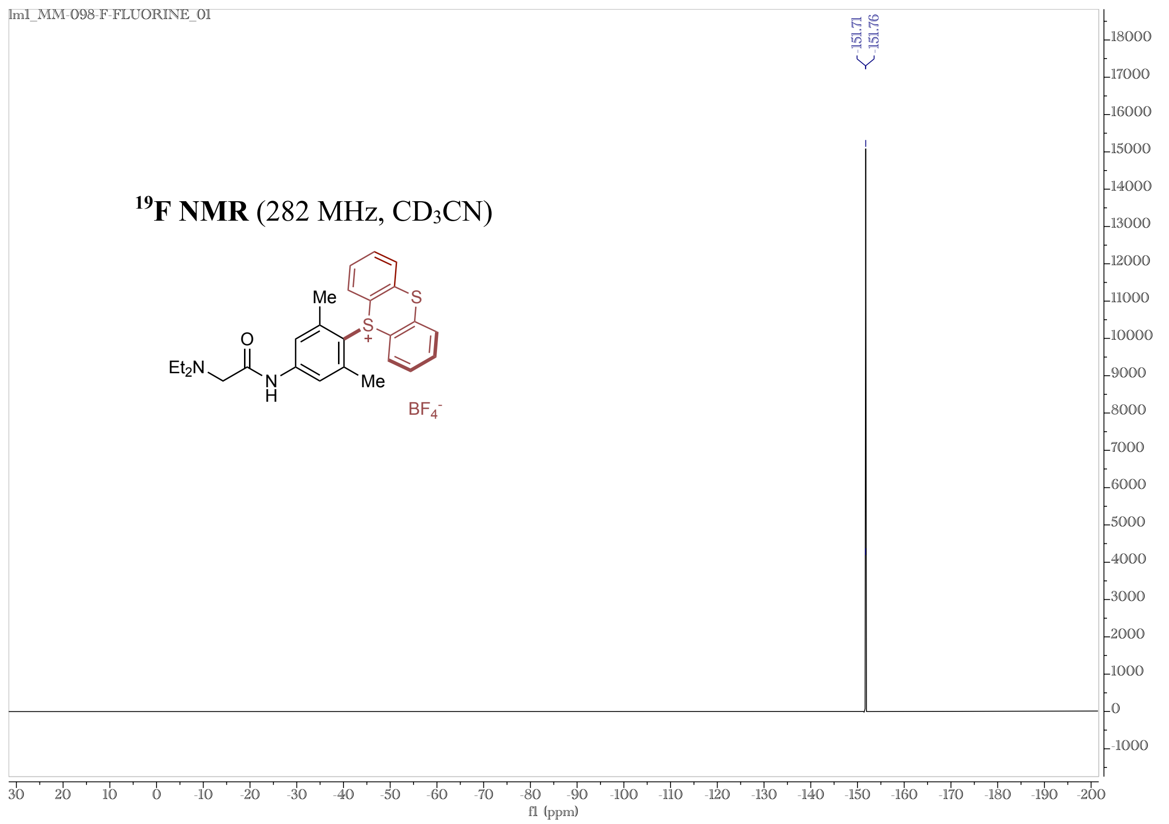

Unmasked lidocaine ^{1}H NMR

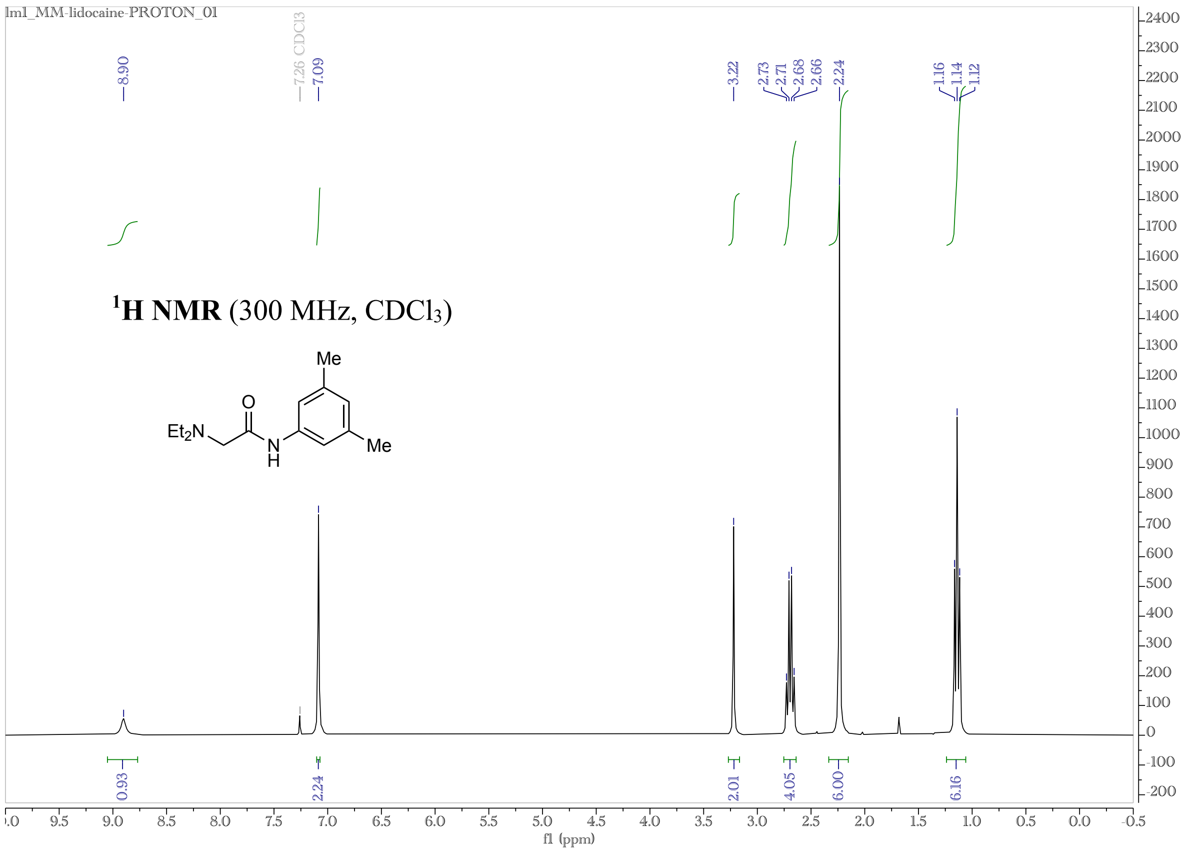

4-Anisole derived dibenzothiophenium salt (SI-2)

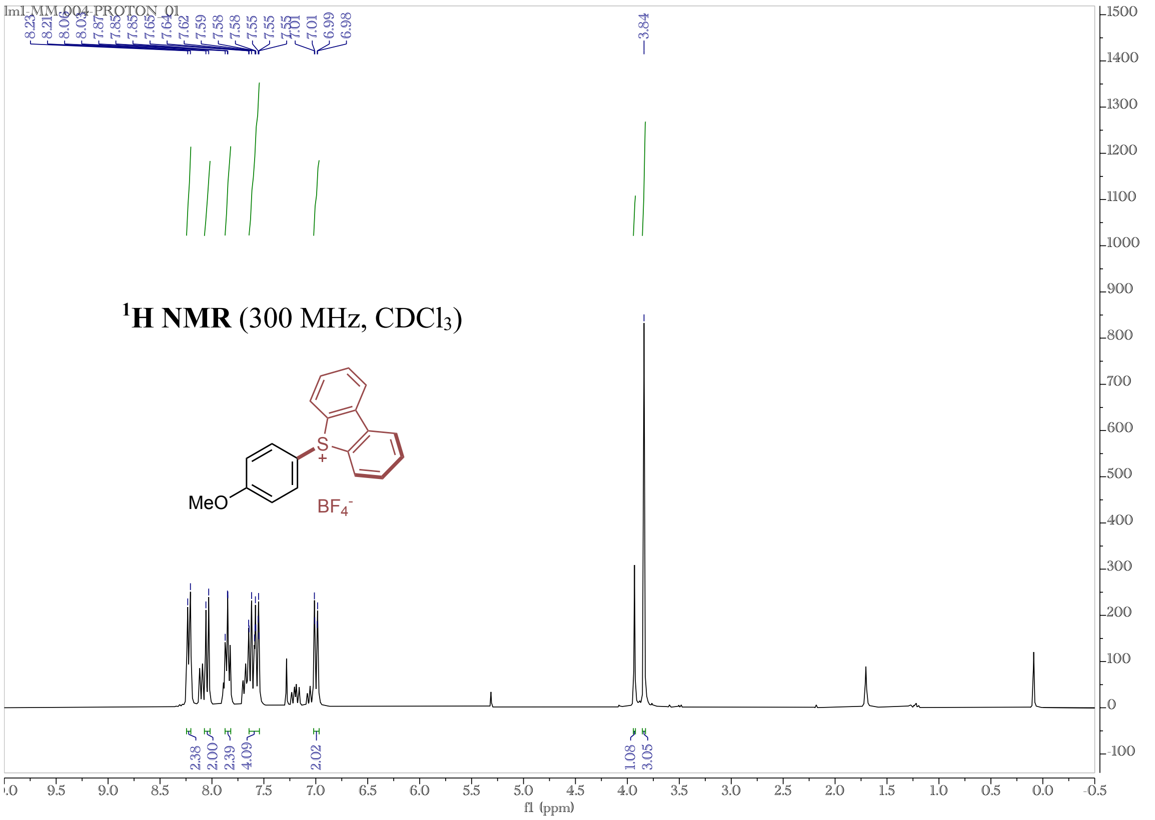

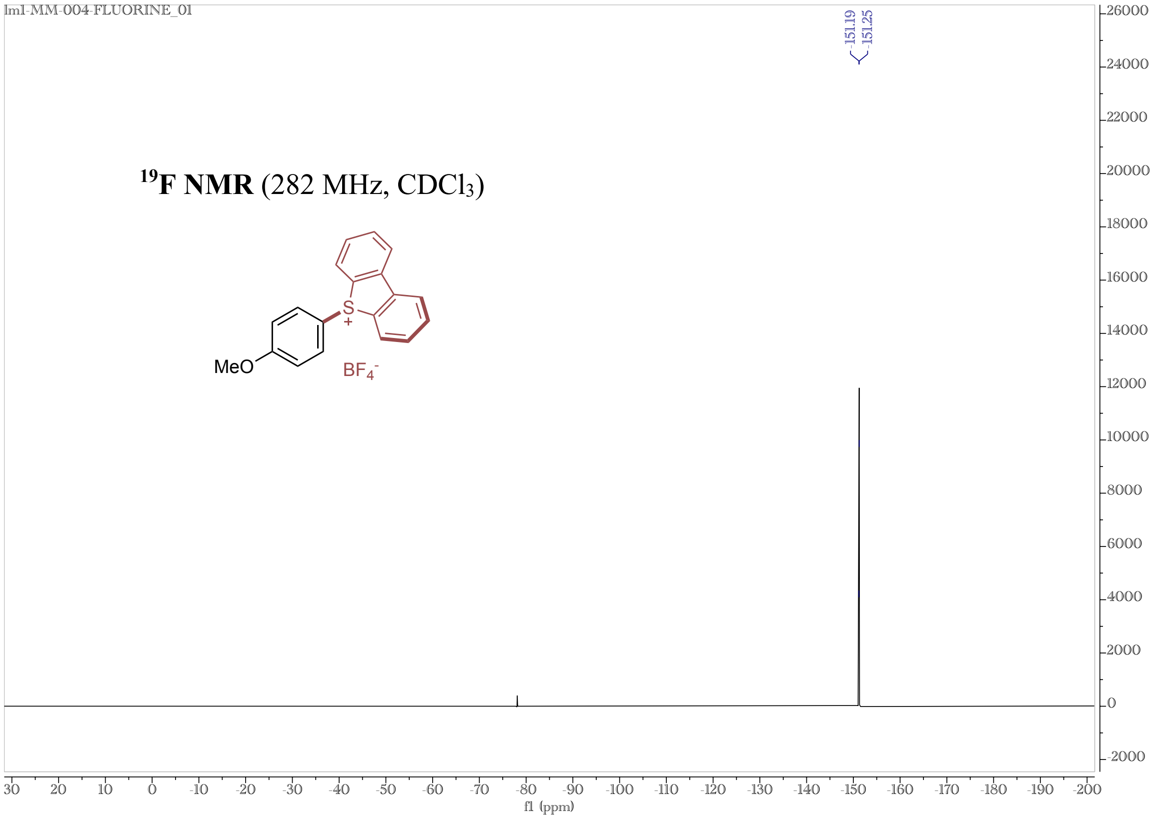

2-(4-(4-Bromophenoxy)phenyl)-1-methyl-1*H*-pyrrole (8)

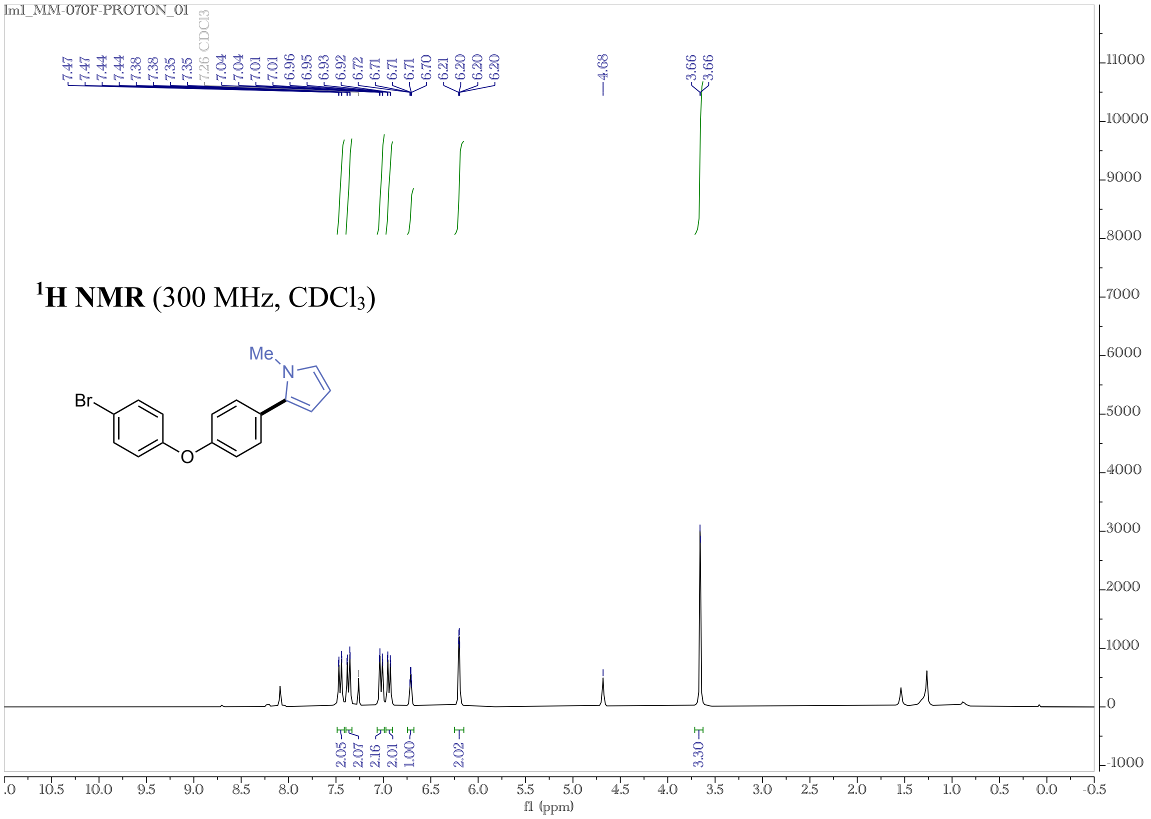

x

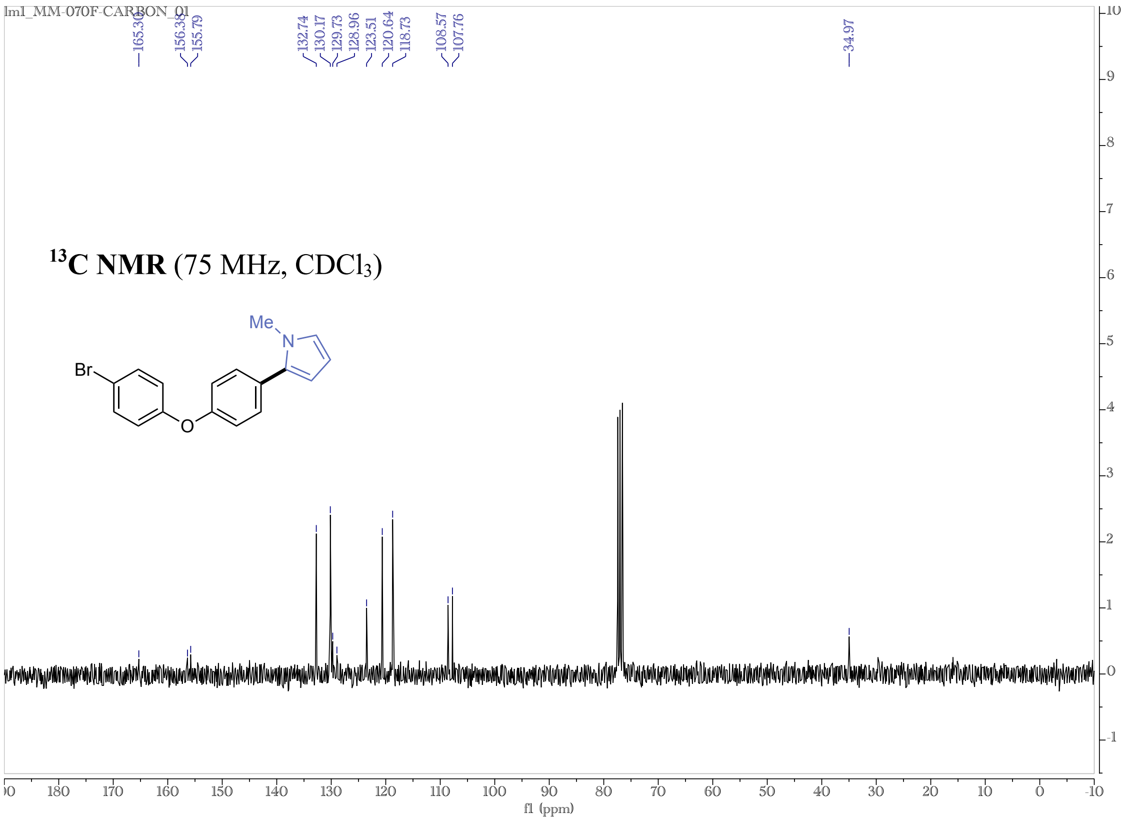

lm1_MM-070F-CARBON_01
165.36
156.38
155.79
132.74
130.17
129.73
128.96
123.51
120.64
118.73
108.57
107.76
34.97
¹³C NMR (75 MHz, CDCl₃)
Me
N
Br
O
f1 (ppm)

1-(4-(4-Bromophenoxy)phenyl)piperidine (10)

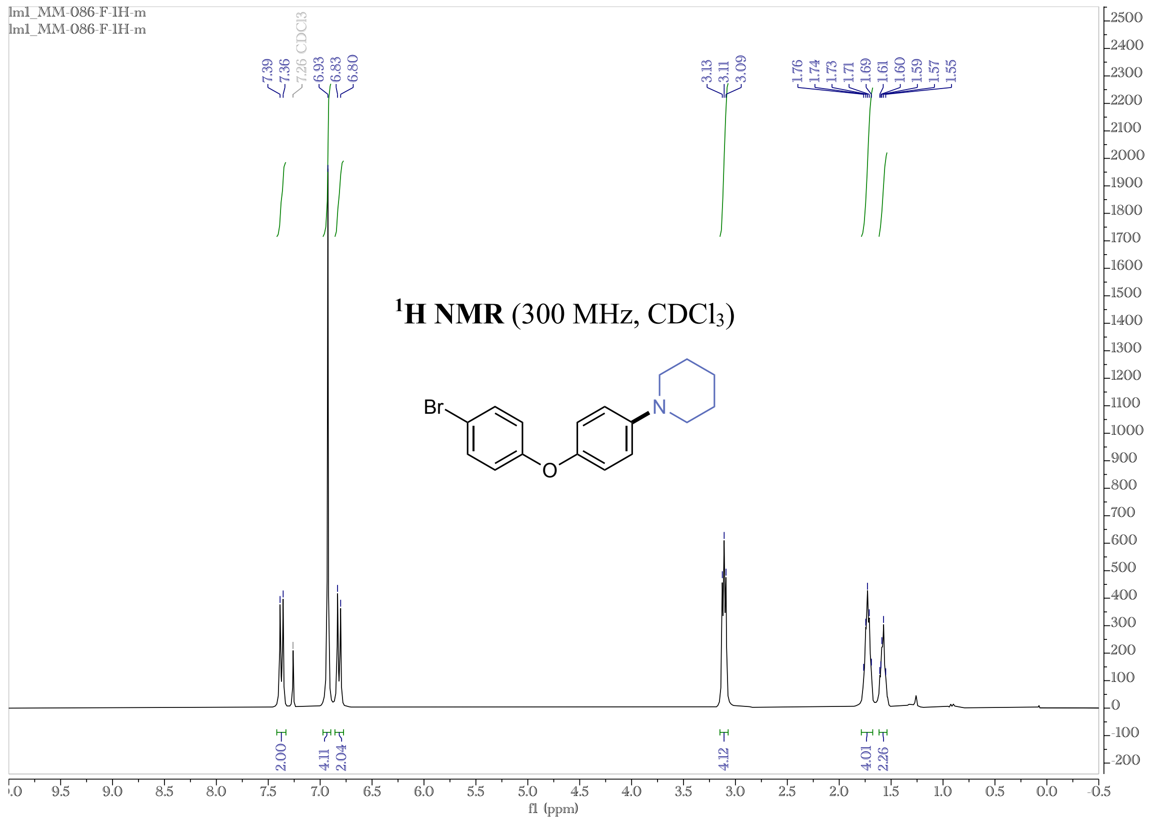

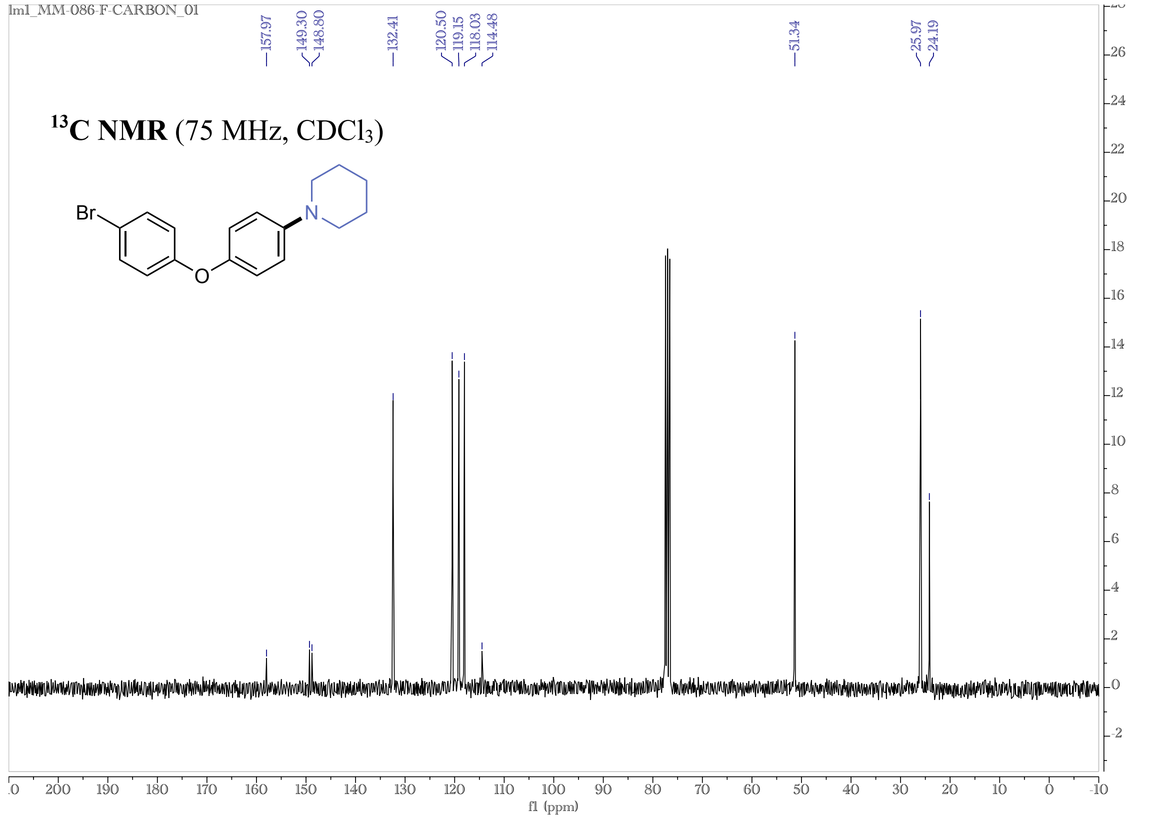

15. Supplementary references

1 Berger, F.; Plutschack, M. B.; Riegger, J.; Yu, W.; Speicher, S.; Ho, M.; Frank, N.; Ritter, T. Site-selective and versatile aromatic C−H functionalization by thianthrenation. *Nature* **2019**, *567*, 223–228.

2 Li, J.; Chen, J.; Sang, R.; Ham, W.-S.; Plutschack, M. B.; Berger, F.; Chabbra, S.; Schnegg, A.; Genicot, C.; Ritter, T. Photoredox catalysis with aryl thianthrenium salts enables site-selective late-stage fluorination. *Nat. Chem* **2020**, *12*, 56–62.

3 Mato, M.; Bruzzese, P. C.; Takahashi, F.; Leutzsch, M.; Reijerse, E. J.; Schnegg, A.; Cornella, J. Oxidative Addition of Aryl Electrophiles into a Red-Light-Active Bismuthinidene. *J. Am. Chem. Soc.* **2023**, *145*, 18742–18747.

4 Kafuta, K.; Korzun, A.; Böhm, M.; Golz, C.; Alcarazo, M. Synthesis, Structure and Reactivity of 5-(Aryl)dibenzothiophenium Triflates. *Angew. Chem. Int. Ed.* **2020**, *59*, 1950–1955.

5 Chen, X.-Y.; Li, Y.-N.; Wu, Y.; Bai, J.; Guo, Y.; Wang, P. Cu-Mediated Thianthrenation and Phenoxathiination of Arylborons. *J. Am. Chem. Soc.* **2023**, *145*, 10431–10440.

6 Tanioka, M.; Kuromiya, A.; Ueda, R.; Obata, T.; Muranaka, A.; Uchiyama, M.; Kamino, S. Bridged eosin Y: a visible and near-infrared photoredox catalyst. *Chem. Commun.* **2022**, *58*, 7825–7828.

7. Baldon, S.; Paut, J.; Anselmi, E.; Dagousset, G.; Tuccio, B.; Pelosi, G.; Cuadros, S.; Magnier, E.; Dell'Amico, L. Radical photochemical difluorosulfoximination of alkenes and propellanes. *Chem. Sci.* **2025**, *16*, 6957–6964.

8. Zhang, X.-F.; Zhang, J.; Liu, L. Fluorescence properties of twenty fluorescein derivatives: lifetime, quantum yield, absorption and emission spectra. *J. Fluoresc.* **2014**, *24*, 819–826.

9 Ni, S.; Halder, R.; Ahmadli, D.; Reijerse, E. J.; Cornella, J.; Ritter, T. C–heteroatom coupling with electron-rich aryls enabled by nickel catalysis and light. *Nat. Catal.* **2024**, *7*, 733–741.

10 D'Avino, C.; Gutiérrez, S.; Feldhaus, M. J.; Tomás-Gamasa, M.; Mascareñas, J. L. Intracellular Synthesis of Indoles Enabled by Visible-Light Photocatalysis. *J. Am. Chem. Soc.* **2024**, *146*, 2895–2900.
